# Supplementary material for: Point-of-care C-reactive protein measurement by community health workers safely reduces antimicrobial use among children with respiratory illness in rural Uganda: A stepped wedge cluster randomized trial
Source: PLoS Med. 2024 Aug 19;21(8):e1004416. doi: 10.1371/journal.pmed.1004416 (PMC11407643; doi:10.1371/journal.pmed.1004416)
Supplement: S1 Fig — Images were hand drawn by author Baguma Emmanuel. (PDF) [file pmed.1004416.s002.pdf]

Figure S1. STAR STUDY SICK CHILD JOB AID FOR INTERVENTION PERIODS

1a. ASK THE CARE GIVER / BULHAYA OYULYALETHA OMWANA

How Old Is The Child?
Omwana awithe
emyaka mingahi?

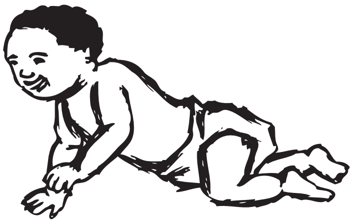

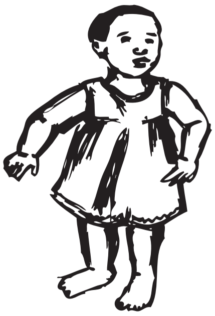

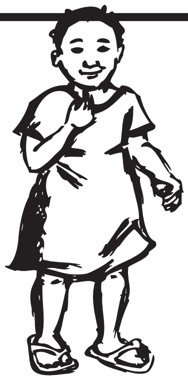

☐ 2-11 months / emighesera

☐ 1-2 years / emyaka

☐ 3-5 years / emyaka

1b. -IF LESS THAN 2 MONTHS, THEY ARE NOT ELIGIBLE FOR THE STAR STUDY

Abana abali ahisi syemighesera ibiri sibendi yiyunga okwamusomo ono

- IF NO CARE GIVER, THEY ARE NOT ELIGIBLE FOR THE STAR STUDY

Amathendyasa noyulyamuletha isyendi yiyunga okwamusomo ono

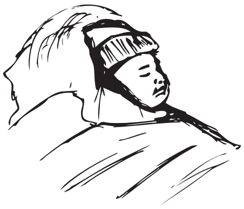

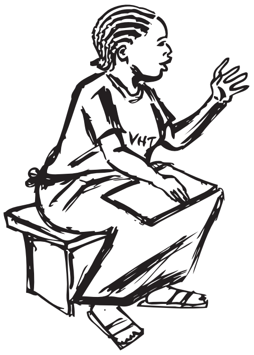

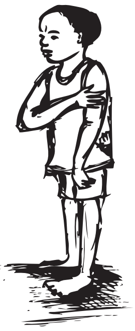

If either of the above things are true, switch to routine sick child job aid
If care giver present and child is 2 months or older, continue with star study Sick Child Job Aid

Ebiri ahaghulhu Byamabya Ibinahikire, Suba Okwandaghiriro Eyabulikiro Eyabayonzesya.

Amasa n'owakamulhabirira kandi Omwana inyanawithe amezi abiri nerilolha Eyighulhu Ghende Embere nendaghiriro Eyomusomo Owe Star Study

2a. ASK CHILD'S PROBLEMS / BULHAYA EBITSIBU EBYOMWANA AWITHE

Does the child have cough, diarrhea and fever
Omwana anemukoholha, erihurungana, kwitse erihuhanako omubiri?

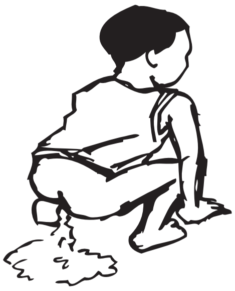

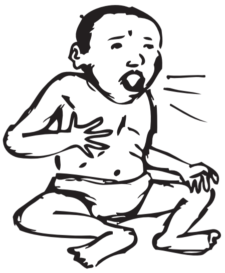

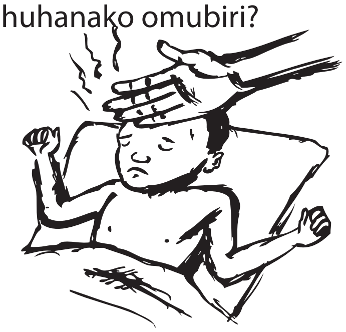

If diarrhea, cough or fever only, they are not eligible for the STAR study
➞switch to routine Sick Child Job Aid

omwana owalwere erihurungana lisa, erikoholha lisa kwitse erihuhanako omubiri lisa akabya isatholere eriyiyunga okwamusomo ono

MUST-UNC RESEARCH COLLABORATION

THE UNIVERSITY
of NORTH CAROLINA
at CHAPEL HILL

2b. ASK CHILD'S PROBLEMS / BULHAYA EBITSIBU EBYOMWANA AWITHE

If cough and fever for 7 days or less, approach care giver about consenting for the study.

Amasagwa inyawithe ekikuba nerihuhana ahabwe biro musanju kwihi bike kanaya noyulyamuletha okwamwatsi weriyiyunga okwa musomo.

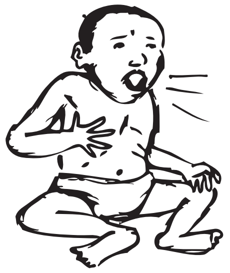

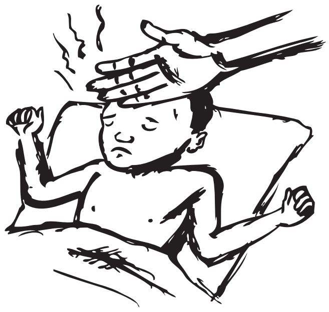

If they do not consent or if they have symptoms for more than 7 days
→ switch to routine Sick Child Job Aid

Amaghana kwitsi omwana amasangwa inawithe obuminyikalho erilhaba okwabiro musanju, ghenda okwa ndayiyiro eyabayonzesya

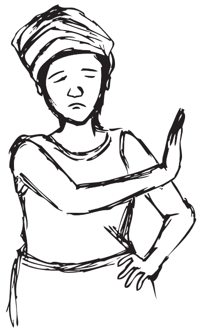

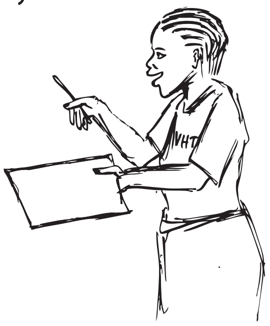

If consented start a data collection form AND perform an MRDT and CRP test

Amikiririya eriyiyunga okwamusomo handika ebihambire okwamwana neryo ghumupime omutsutsa ne kipimo ekye CRP

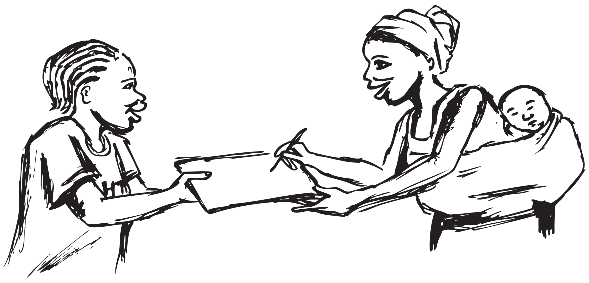

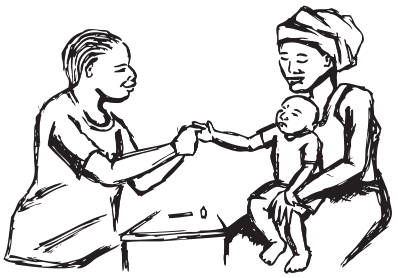

Check for fast breathing and temperature (also heart rate and oxygen saturation if the VHT has a pulse oximeter)

Kebera erihumulha lyo mwana no buhuhane (hayima neri swirya lyo mutima no muka omuyonzesya amasangwa inyana withe ebyeripimamu)

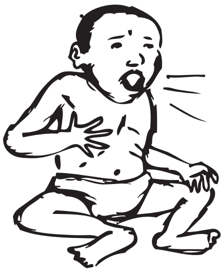

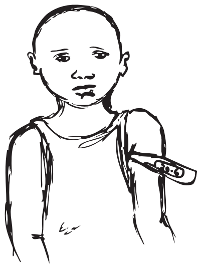

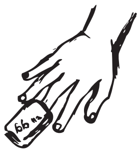

3. ASK AND LOOK FOR DANGER SIGNS AND REFER/ BULHAYA KANDI USAMALIRE OMWANA NGANEKO EBISOSEKANO EBYERITHOHA NERYO IWAMUTHUMA OKWITHAMBIRIRO

a. If vomiting everything, severe chest in-drawing, convulsions, not able to drink or breastfeed, very sleepy, uncounscious, difficult to wake → give a referral treatment(same as section 4a on the usual sick child job aid) and refer to health facility

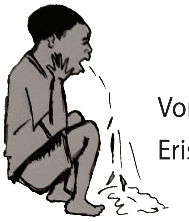

Vomits everything
Erisala Obulikalyo kosi

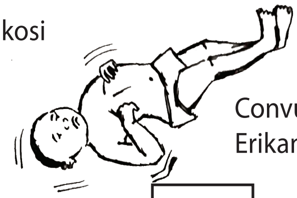

Convulsions
Erikangabalha

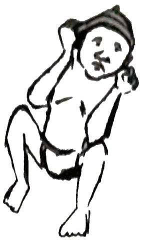

Chest in-drawing
Ekikuba ekyo mwana
Erisuba Omwakathi
kutsibu Akahumulha

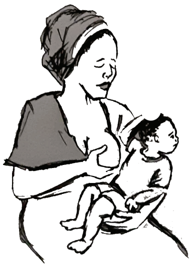

Not able
to breastfeed or Drink
mwana erighana eryonga
ıtsi Erinywa

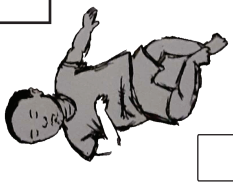

Very sleepy or uncounscious/
Difficult to wake.
Omwana erisiba Omwathulho
kutsi Erithoha

OTHER DANGER SIGNS(EBINDI EBIMINYIKALO EBYERI THOHA)

1. 02 sat <90 % (erihumulha ahisi okwa mirundi kyenda)

2. Very fast breathing(RR>70 breaths per minute for age 2-1 1 months

and RR>50 for age 1-5 years) Erihumulha lhuba lhuba emirundi eyilhabire okwa sanju okwa baana abali ahakathikathi ke mighesera ibiri erihika okwikumi nalighuma, kandi emirundi eyilhabire okwa makumi athanu okwa bana abali ahakathikathi yo mwaka mughuma eri hika okwi thanu)

3. Malnutrition(MUAC 11.5 cm or red) Eryuse

MUST-UNC RESEARCH COLLABORATION
THE UNIVERSITY of NORTH CAROLINA at CHAPEL HILL

4. IF NO DANGER SIGNS, TREAT AND ADVISE - HAMATHENDIBYA EBISOSEKANO EBYERITHOHA JANJABA KANDI GHUHABULHE

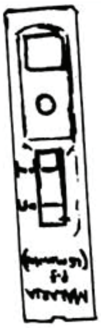

a. If MRDT Positive - Oral anti malarial ACT if >/= 4 months of age
Omuthutsa amabanikamo -amabya inyanawithe emighesera ikumi nerilholha eyighulu ghumuherer aye omubatsi

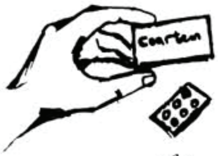

i. Give 1 dose twice daily for 3 days. Hereraya ekinini kighuma kabiri ekiro habwe biro bisathu
4 months to 3 years (emighesera ini erihika okwa myaka isathu)
1 tab from **YELLOW PACK** -ekinini kighuma erilwa omwakabokisi akekisande

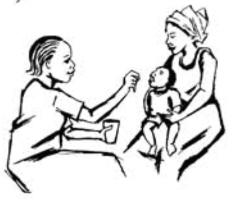

3-5 years 2 tabs from **BLUE PACK** / Emyaka isyathu eri hika okwithanu ebinini bibirti omwa ksbokisi akebulu

ii. Help care giver give first dose now -Ghuwathikaye owalyaletha omwana erihererya edozi eyokubanza hatya hatya

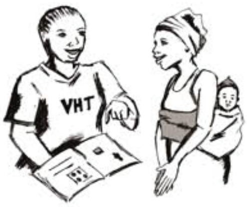

iii. Advise care giver on use of bednet - Ghuhabulhe Oyulyaletha Omwana Okwikolesya Akatimba kemibu

b. IF CRP >/= 40 (EITHER MRDT POSITIVE OR MRDT NEGATIVE) GIVE AMOXICILLIN - EKIBIMO KYAMAKANGANIA KITHI KIRI OMWA MAKUMI ANI NERILOLHA EYIGHULHU(OMUTSUTSA NOMU ANGABYAMO KUSTI ESYALIMO)GHUMUHE AMOXICILLIN

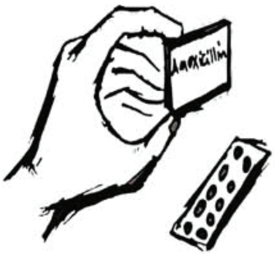

i. 2-11 months: 2 tabs from red pack - Emighesera ibiri erihika Okwi kumi na mughuma ghubahe ebijuma bibiri ebiri Omwa kapaketi Akomusasi

ii. 1-5 years: 3 tabs from green pack - Omwaka mughuma erihika okwi thanu, ghubahe ebijuma bisathu ebiri omwa kapaketi ake kibabi.

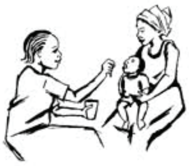

iii. Help care giver give first dose now Ghuwathikaye Owalyaletha Omwana Erihererya edozi eyokubanza hathya hathya

c. If CRP is <40(either MRDT Positve or MRDT Negative) give advise for all children treated at home(same as section 5) only

Ekipimo Kyamakangania Kithi Kiri Omwa Makumi Ani Nerilolha ahisi Omutsutsa Nomu Angabyamo Kusti Esyalimo) ghuhereraye erihabulha okwabana abakajanjabirawa Eka (Ngoku kinakanganibwe omwa kaseseka akakathanu) Kisa

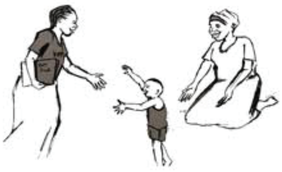

i. Make a plan for follow up of the child in 7 days Ghukole endegheka eyerikwamira Omwana ahanyuma yebiro musanju

NOTE: all children age 2 to 4 months with fever should be reffered regardless of mRDT result as per iCCM-abana abali ahakathiklathi ke mighesera ibiri erihika okwini,batholere iba lebekwa okwa sipatara omutsutsa amabanika mo kutse nomwanga thendibanikamo
