# Supplementary material for: Point-of-care C-reactive protein measurement by community health workers safely reduces antimicrobial use among children with respiratory illness in rural Uganda: A stepped wedge cluster randomized trial
Source: PLoS Med. 2024 Aug 19;21(8):e1004416. doi: 10.1371/journal.pmed.1004416 (PMC11407643; doi:10.1371/journal.pmed.1004416)
Supplement: S2 Fig — The study began enrollment on November 1, 2021 and ended enrollment on May 12, 2022 with each period lasting approximately 1 month. Yellow blocks represent control periods; blue blocks are intervention periods. The number shown within the blocks is the total number of participants enrolled for each sequence and period. The number of CHW in each sequence is shown in parentheses next to the name of the villages. (DOCX) [file pmed.1004416.s003.docx]

**Figure S2. Number of children enrolled per sequence and period**. The study began enrollment on November 1, 2021 and ended enrollment on May 12, 2022 with each period lasting approximately 1 month. Yellow blocks represent control periods; blue blocks are intervention periods. The number shown within the blocks is the total number of participants enrolled for each sequence and period. The number of CHW in each sequence is shown in parentheses next to the name of the villages.

|  |  | Period (Months) | | | | | |  |  |
| --- | --- | --- | --- | --- | --- | --- | --- | --- | --- |
| Sequence | Villages (Number of CHW) | 1 | 2 | 3 | 4 | 5 | 6 | **Total** | Switch Date |
| 1 | Ndughutu West, Kibirizi, Bunyangoni (10) | 50 | 87 | 67 | 77 | 16 | 20 | **317** | 1 Dec 2021 |
| 2 | Muramba I, Nyakabugha, Ruboni (15) | 36 | 39 | 35 | 62 | 24 | 28 | **224** | 11 Jan 2022 |
| 3 | Kanyaminigho, Kirongo, Nyangonge (13) | 54 | 66 | 31 | 55 | 31 | 24 | **261** | 8 Feb 2022 |
| 4 | Ihani, Katooke II, Murimbo (14) | 44 | 67 | 52 | 33 | 44 | 25 | **265** | 8 March 2022 |
| 5 | Bugoye, Rwakingi 1B, Kisamba II (13) | 38 | 24 | 26 | 16 | 11 | 38 | **153** | 11 April 2022 |
| **Total** |  | **222** | **283** | **211** | **243** | **126** | **135** | **1220** |  |
