# Supplementary material for: Point-of-care C-reactive protein measurement by community health workers safely reduces antimicrobial use among children with respiratory illness in rural Uganda: A stepped wedge cluster randomized trial
Source: PLoS Med. 2024 Aug 19;21(8):e1004416. doi: 10.1371/journal.pmed.1004416 (PMC11407643; doi:10.1371/journal.pmed.1004416)
Supplement: S3 Fig — The number of children enrolled by each CHW grouped by village and treatment sequence. CHW were randomly assigned numbers 1 through 5 for this figure. (DOCX) [file pmed.1004416.s004.docx]

**Figure S3. Study enrollment by participating community health worker (CHW).** The number of children enrolled by each CHW grouped by village and treatment sequence. CHW were randomly assigned numbers 1 through 5 for this figure.

**
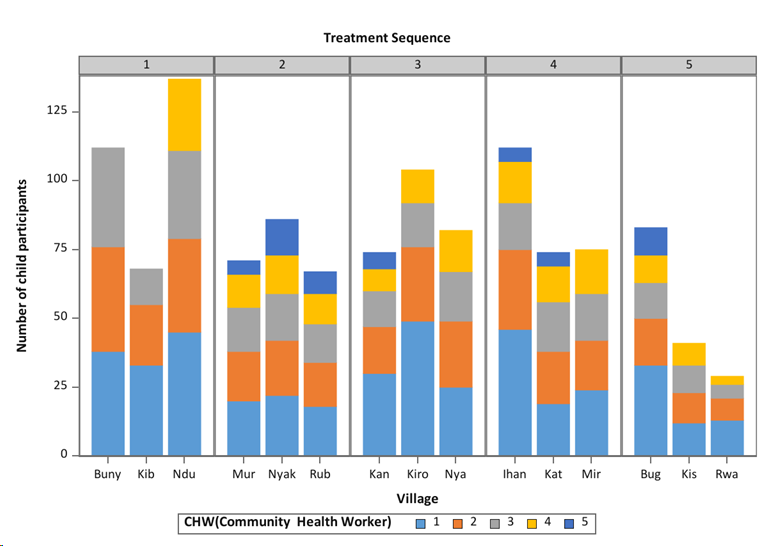
**
