# Supplementary material for: Point-of-care C-reactive protein measurement by community health workers safely reduces antimicrobial use among children with respiratory illness in rural Uganda: A stepped wedge cluster randomized trial
Source: PLoS Med. 2024 Aug 19;21(8):e1004416. doi: 10.1371/journal.pmed.1004416 (PMC11407643; doi:10.1371/journal.pmed.1004416)
Supplement: S4 Fig — Yellow blocks represent control periods; blue blocks are intervention periods. (DOCX) [file pmed.1004416.s005.docx]

**Figure S4.** **Percentage of children who were given or prescribed antibiotics by treatment sequence and period.** Yellow blocks represent control periods; blue blocks are intervention periods.

|  |  | Period | | | | | |  |
| --- | --- | --- | --- | --- | --- | --- | --- | --- |
| Sequence | Village (Clusters) | 1 | 2 | 3 | 4 | 5 | 6 | Treatment Switch Date |
| 1 | Ndugutu West, Kibirizi, Bunyangoni | 45/50  90% | 57/87  65.5% | 46/67  68.7% | 52/77  67.5% | 13/16  81.3% | 15/20  75% | 1 Dec 2021 |
| 2 | Muramba I, Nyakabugha, Ruboni | 32/36  89.9% | 31/39  79.5% | 28/35  80% | 42/62  67.7% | 23/24  95.8% | 22/28  78.6% | 11 Jan 2022 |
| 3 | Kanyaminigo, Kirongo, Nyangonge | 49/54  90.7% | 59/66  89.4% | 29/31  93.5% | 27/55  49.1% | 20/31  64.5% | 19/24  79.2% | 8 Feb 2022 |
| 4 | Ihani, Katooke II, Mirimbo | 43/44  97.7% | 61/67  91.0% | 47/52  90.4% | 32/33  97.0% | 36/44  81.8% | 17/25  68.0% | 8 March 2022 |
| 5 | Bugoye, Rwakingi, Kisamba II | 36/38  94.7% | 24/24  100% | 24/26  92.3% | 16/16  100% | 11/11  100% | 31/38  81.6% | 11 April 2022 |
