# Supplementary material for: Point-of-care C-reactive protein measurement by community health workers safely reduces antimicrobial use among children with respiratory illness in rural Uganda: A stepped wedge cluster randomized trial
Source: PLoS Med. 2024 Aug 19;21(8):e1004416. doi: 10.1371/journal.pmed.1004416 (PMC11407643; doi:10.1371/journal.pmed.1004416)
Supplement: S5 Fig — Yellow blocks represent control periods; blue blocks are intervention periods. (DOCX) [file pmed.1004416.s006.docx]

**Figure S5.** **Percentage of children who were given or prescribed antibiotics by village.** Yellow blocks represent control periods; blue blocks are intervention periods.

|  |  | Period | | | | | |  |
| --- | --- | --- | --- | --- | --- | --- | --- | --- |
| Sequence | Village (Clusters) | 1 | 2 | 3 | 4 | 5 | 6 | Treatment Switch Date |
| 1 | Ndugutu West | 14/16  87.5% | 31/45  68.9% | 21/31  67.7% | 20/29  69.0% | 6/7  85.7% | 7/9  77.8% | 1 Dec 2021 |
|  | Kibirizi | 11/11  100% | 10/12  83.3% | 13/16  81.3% | 18/22  81.8% | 3/4  75% | 3/3  100% |  |
|  | Bunyangoni | 20/23  87.0% | 16/30  53.3% | 12/20  60% | 14/26  53.9% | 4/5  80% | 5/8  62.5% |  |
| 2 | Muramba I | 13/15  86.7% | 8/12  66.7% | 8/11  72.7% | 15/17  88.2% | 8/8  100% | 8/8  100% | 11 Jan 2022 |
|  | Nyakabugha | 10/10  100% | 13/15  86.7% | 17/21  81.0% | 17/22  77.3% | 9/9  100% | 8/9  88.9% |  |
|  | Ruboni | 9/11  81.8% | 10/12  83.3% | 3/3  100% | 10/23  43.5% | 6/7  85.7% | 6/11  54.6% |  |
| 3 | Kanyaminigo | 17/17  100% | 19/20  95% | 6/7  85.7% | 8/10  80% | 8/11  72.7% | 8/10  80% | 8 Feb 2022 |
|  | Kirongo | 24/28  85.7% | 25/31  80.7% | 6/7  85.7% | 7/18  38.9% | 8/12  66.7% | 7/8  87.5% |  |
|  | Nyangonge | 8/9  88.9% | 15/15  100% | 17/17  100% | 12/27  44.4% | 4/8  50% | 4/6  66.7% |  |
| 4 | Ihani | 21/21  100% | 33/34  97.1% | 15/15  100% | 18/18  100% | 18/19  94.7% | 5/6  83.3% | 8 March 2022 |
|  | Katooke II | 9/10  90% | 11/16  68.8% | 19/24  79.2% | 6/7  85.7% | 6/10  60% | 7/8  87.5% |  |
|  | Mirimbo | 13/13  100% | 17/17  100% | 13/13  100% | 8/8  100% | 12/15  80% | 5/11  45.5% |  |
| 5 | Bugoye | 21/23 91.3% | 13/13  100% | 16/18  88.8% | 11/11  100% | 5/5  100% | 10/13  76.9% | 11 April 2022 |
|  | Rwakingi | 8/8  100% | 6/6  100% | 1/1  100% | 2/2  100% | 1/1  100% | 8/11  72.7% |  |
|  | Kisamba II | 7/7  100% | 5/5  100% | 7/7  100% | 5/5  100% | 5/5  100% | 13/14  92.8% |  |
