# Supplementary material for: Point-of-care C-reactive protein measurement by community health workers safely reduces antimicrobial use among children with respiratory illness in rural Uganda: A stepped wedge cluster randomized trial
Source: PLoS Med. 2024 Aug 19;21(8):e1004416. doi: 10.1371/journal.pmed.1004416 (PMC11407643; doi:10.1371/journal.pmed.1004416)
Supplement: S1 Table — One village was randomly selected from each stratum for each sequence. Proportions of antibiotic use shown are unadjusted. (DOCX) [file pmed.1004416.s007.docx]

**Table S1. Scheme for stratification of participating villages**. One village was randomly selected from each stratum for each sequence. Proportions of antibiotic use shown are unadjusted.

| **Stratum** | **Characteristics** | | | **Villages** | **Antibiotic Use**  **(Control Group)** | **Antibiotic Use**  **(Intervention Group)** |
| --- | --- | --- | --- | --- | --- | --- |
|  | **Altitude** | **Distance from BHC** | **Size^1^** |  |  |  |
| **A** | Low | Proximal | Large (>115) | Bugoye, Ihani, Kanyaminigo, Muramba I, Ndugutu West | 230/245  (93.9%) | 181/234  (77.4%) |
| **B** | Low | Mid-distance | Medium  (90-140) | Rwakingi 1B, Nyakabugha, Kibirizi, Katooke II, Kirongo | 152/177  (85.9%) | 141/185  (76.2%) |
| **C** | High | Distal | Small  (<120) | Ruboni, Kisamba II, Nyangonge, Bunyangoni, Mirimbo | 157/165  (95.2%) | 126/214  (58.9%) |

^1^ Number of eligible children seen per year based on the past year’s data

BHC: Bugoye Health Center III (red triangle on map in Figure 1)
