# Supplementary material for: Point-of-care C-reactive protein measurement by community health workers safely reduces antimicrobial use among children with respiratory illness in rural Uganda: A stepped wedge cluster randomized trial
Source: PLoS Med. 2024 Aug 19;21(8):e1004416. doi: 10.1371/journal.pmed.1004416 (PMC11407643; doi:10.1371/journal.pmed.1004416)
Supplement: S2 Table — Continuous variables are shown as medians with interquartile ranges, whereas categorical variables are shown as n (%). n = 65 except where specified. (DOCX) [file pmed.1004416.s008.docx]

**Table S2. Demographic and occupational characteristics of the CHW who participated in the STAR Study.** Continuous variables are shown as medians with interquartile ranges, whereas categorical variables are shown as n (%). n=65 except where specified.

| Characteristic |  |
| --- | --- |
| Age (years) | 40 (35-47) |
| Experience as CHW (years) | 10 (4-14) |
| *Missing* | 5 |
| Children Seen per Month | 10 (7-16) |
| *Missing* | 5 |
| Sex |  |
| Male | 25 (38.5) |
| Female | 40 (61.5) |
| Marital Status |  |
| Single | 3 (4.6) |
| Married | 60 (92.3) |
| Widowed | 2 (3.1) |
| Education Level |  |
| Primary School | 9 (13.9) |
| Secondary School | 54 (83.1) |
| University | 2 (3.1) |
| Primary Occupation |  |
| Subsistence Farmer | 55 (84.6) |
| Business Owner | 1 (1.5) |
| Teacher | 1 (1.5) |
| Tailor | 2 (3.1) |
| Other | 6 (9.2) |
