# Supplementary material for: Point-of-care C-reactive protein measurement by community health workers safely reduces antimicrobial use among children with respiratory illness in rural Uganda: A stepped wedge cluster randomized trial
Source: PLoS Med. 2024 Aug 19;21(8):e1004416. doi: 10.1371/journal.pmed.1004416 (PMC11407643; doi:10.1371/journal.pmed.1004416)
Supplement: S3 Table — (DOCX) [file pmed.1004416.s009.docx]

**Table S3. Percentage of participants who were given or prescribed antimalarial treatment by mRDT result and treatment condition.**

| **Overall** | | |
| --- | --- | --- |
|  | **mRDT -**  (n=590) | **mRDT +**  (n=628) |
| Antimalarial given | 12 (2.0%) | 593 (94.4%) |
| Antimalarial NOT given | 578 (98.0%) | 35 (5.6%) |
| **Control** | | |
|  | **mRDT -**  (n=277) | **mRDT +**  (n=310) |
| Antimalarial given | 3 (1.1%) | 303 (97.7%) |
| Antimalarial NOT given | 274 (98.9%) | 7 (2.3%) |
| **Intervention** | | |
|  | **mRDT -**  (n=313) | **mRDT +**  (n=318) |
| Antimalarial given | 9 (2.9%) | 290 (91.2%) |
| Antimalarial NOT given | 304 (97.1%) | 28 (8.8%) |
