# Supplementary material for: Point-of-care C-reactive protein measurement by community health workers safely reduces antimicrobial use among children with respiratory illness in rural Uganda: A stepped wedge cluster randomized trial
Source: PLoS Med. 2024 Aug 19;21(8):e1004416. doi: 10.1371/journal.pmed.1004416 (PMC11407643; doi:10.1371/journal.pmed.1004416)
Supplement: S4 Table — (DOCX) [file pmed.1004416.s010.docx]

**Table S4.** **Generalized estimating equations (GEE)^1^ estimates and standard errors^2^ for logistic regression of antibiotic use**

|  | Adjusted for period | | | Adjusted for period and village strata | | |
| --- | --- | --- | --- | --- | --- | --- |
| ***Marginal logistic regression model*** | | | | | | |
| Variable | Estimate | SE-BC1 | P- Value | Estimate | SE-BC1 | P- Value |
| Intercept | 2.56 | 0.27 | <.001 | 3.10 | 0.29 | <.001 |
| Period 2 | -0.28 | 0.33 | 0.432 | -0.28 | 0.35 | 0.449 |
| Period 3 | 0.05 | 0.36 | 0.891 | 0.03 | 0.37 | 0.947 |
| Period 4 | -0.20 | 0.28 | 0.491 | -0.20 | 0.31 | 0.550 |
| Period 5 | 0.56 | 0.40 | 0.199 | 0.56 | 0.43 | 0.238 |
| Period 6 | 0.37 | 0.34 | 0.328 | 0.37 | 0.38 | 0.350 |
| Village Strata B | - | - | - | -0.94 | 0.45 | 0.330 |
| Village Strata C | - | - | - | -1.72 | 0.31 | 0.023 |
| Intervention, δ | -1.703 | 0.395 | 0.003 | -1.725 | 0.417 | 0.006 |
| ***Within-village correlations*** | | | | | | |
|  | Estimate | SE-BC2 | P-Value | Estimate | SE-BC2 | P- Value |
| Within-period | 0.053 | 0.026 | 0.067 | 0.045 | 0.024 | 0.087 |
| Between-periods | 0.039 | 0.018 | 0.046 | 0.040 | 0.018 | 0.047 |

^1^ Results were obtained using the SAS macro GEEMAEE for paired estimating equations with bias-corrected variance estimator with the option “MAEE” (matrix-adjusted estimating equations of Preisser et al., 2008) that corrects for the finite-sample bias of ICC estimates; this analysis assumes a nested exchangeable correlation structure.

**^2^** Bias-corrected standard errors are based on the procedures of Kauermann and Carroll (2001) and Mancl and DeRouen (2001).
