# Supplementary material for: Point-of-care C-reactive protein measurement by community health workers safely reduces antimicrobial use among children with respiratory illness in rural Uganda: A stepped wedge cluster randomized trial
Source: PLoS Med. 2024 Aug 19;21(8):e1004416. doi: 10.1371/journal.pmed.1004416 (PMC11407643; doi:10.1371/journal.pmed.1004416)
Supplement: S5 Table — (DOCX) [file pmed.1004416.s011.docx]

**Table S5.** **Frequency of secondary outcomes and estimated odds ratios for association with the intervention.**

| **Outcome** | **Observed Proportions** | | **Intervention Odds Ratio (95% CI)** | **p-value** |
| --- | --- | --- | --- | --- |
|  | *Control*  *(n=587)* | *Intervention*  *(n=633)* |  |  |
| *Pre-specified* |  |  |  |  |
| Clinical failure | 23/585 (3.9%) | 11/630 (1.8%) | 0.41 (0.09, 1.83)^1^ | 0.300 |
| Persistence of fever | 9/585 (1.5%) | 6/630 (1.0%) | 0.75 (0.08, 7.62)^1^ | 1.000 |
| Development of danger signs | 10/585 (1.7%) | 3/630 (0.5%) | 0.32 (0.02, 5.12)^1^ | 0.635 |
| Hospitalization | 8/581 (1.4%) | 5/630 (0.8%) | 0.39 (0.03, 4.83)^1^ | 0.687 |
| Lack of improvement perceived by caregiver | 12/584 (2.1%) | 22/627 (3.5%) | 1.49 (0.37, 6.52)^1^ | 0.754 |
| *Post-hoc* |  |  |  |  |
| Alternate clinical failure | 25/585 (4.3%) | 26/630 (4.1%) | 0.79 (0.25, 2.54)^1^ | 0.849 |
| Need for further outpatient evaluation | 32/585 (5.5%) | 33/630 (5.2%) | 1.50 (0.54, 4.26)^1^ | 0.533 |
| With traditional healer | 3/583 (0.5%) | 5/629 (0.8%) | 3.80 (0.15, 211.50)^1^ | 0.726 |
| At a drug shop | 8/585 (1.4%) | 9/630 (1.4%) | 0.41 (0.05, 3.36)^1^ | 0.562 |
| At a health facility | 23/585 (3.9%) | 25/630 (4.0%) | 2.53 (0.75, 9.06)^1^ | 0.156 |
| Persistence of any symptom other than fever | 11/585 (1.9%) | 14/630 (2.2%) | 0.71 (0.14, 3.63)^1^ | 0.890 |
| Tachypnea at follow-up visit | 70/414 (16.9%) | 78/460 (17.0%) | 1.23 (0.71, 2.15)^2^ | 0.427 |

^1^ Rare outcome, so used an exact inference for stratified logistic regression with linear period effects and villages as strata.

^2^ Using a time-adjusted logistic regression model that includes linear period effects, assuming a nested exchangeable working correlation structure and are estimated with GEE/MAEE. The 95% CI limits are exponents of where *SE* is the finite-sample bias-corrected estimator of Carroll and Kauermann (2001).
