# Supplementary material for: Point-of-care C-reactive protein measurement by community health workers safely reduces antimicrobial use among children with respiratory illness in rural Uganda: A stepped wedge cluster randomized trial
Source: PLoS Med. 2024 Aug 19;21(8):e1004416. doi: 10.1371/journal.pmed.1004416 (PMC11407643; doi:10.1371/journal.pmed.1004416)
Supplement: S1 Appendix — (DOCX) [file pmed.1004416.s012.docx]

Appendix S1. Supplemental Methods

“**Point-of-care C-reactive protein measurement by community health workers safely reduces antimicrobial use among children with respiratory illness in rural Uganda: a stepped wedge cluster randomized trial.**”

By Ciccone et al.

**Stepped Wedge Clustered Randomized Trial Design**

The STAR study was a cluster randomized trial of 15 villages using a repeated cross-sectional stepped wedge design over six periods (months) where different eligible children are enrolled during each month (**Figure 2** in main text). Cluster randomization minimizes treatment contamination between individuals and facilitates administrative convenience. A stepped wedge design was chosen for STAR to facilitate cluster (village) recruitment and because it was a logistically feasible design by switching three villages monthly from control to intervention condition in a staggered fashion. The cross-sectional aspect of the design means that while individual children are in the trial for a short time (7-day follow-up assessment), new children are continually enrolled as they present to the clinics with fever and respiratory symptoms.

**Pre-planned Population-averaged Logistic Regression Model for Primary Outcome**

The primary outcome, antibiotic use (antibiotic prescribing) at the initial child visit, was dichotomous (yes/no). The statistical analysis for it was based on generalized estimating equations (GEE). Specifically, a marginal logistic regression model was posited to describe how the average response (proportion of children with antibiotic use) changes across the subsets of the population defined by the intervention and control cluster-periods. Specifically, let μ_ijk_ be the mean response (or probability of using antibiotics) in the i-th village and j-th period/month (j=1,…, 6) for the k-th child such that

logit(μ_ijk_) = β_j_ + X_1ij_ δ + X_2ij_β_7_ + X_3ij_β_8_, j = 1,…,6

where β_j_, j = 1,…,6, is the effect of the j-th period, X_1ij_ is the treatment indicator of cluster i in period j (X_1ij_=1 if village i receives intervention in month j and 0 otherwise), X_2ij_=1 if village i is in randomization stratum 2 versus X_2ij_=0 otherwise; and X_3ij_=1 if village i is in stratum 3 versus X_3ij_=0 (with stratum 1 as the reference). In this model, δ is the log odds ratio of the ‘’average” intervention effect relative to control that does not depend on the village’s time-on-treatment; given that the intervention is expected to reduce antibiotic use, we anticipated δ<0, and correspondingly, the odds ratio of the intervention effect on antibiotic use for children in the intervention versus the control treatment condition as exp(δ)<1. Note that in this model representation there is not a true intercept but rather the period effect parameters are (β_1_, β_2_, β_3_, β_4_, β_5_, β_6_), which correspond to the log odds of the outcome in the control treatment condition at the respective time periods.

The particular implementation of GEE adjusted for clustering within villages by estimating a within-village, nested exchangeable correlation structure that specified a within-period correlation and a between-period correlation for outcomes from participant pairs in the same village (Li et. al. 2018). These two correlation parameters were estimated with matrix-adjusted estimating equations (MAEE) [1,2] as the correlation estimation step within the iteratively reweighted least squares estimation algorithm of GEE; the use of MAEE provides finite-sample bias corrections of correlation parameters and analysis was performed using the SAS macro GEEMAEE [2]. The odds ratio for the intervention effect and its 95% confidence interval was computed using t-scores with 6 degrees of freedom (the total number of villages or clusters minus the number of parameters estimated in the logistic model). In addition, bias-corrected variance estimators for both mean (Kauermann and Carroll [3]; denoted BC1), and correlation model parameter estimates (Mancl and Derouen [4]; denoted BC2), were reported because the usual uncorrected sandwich variance estimators are known to underestimate the variance when the number of clusters is small, as in the STAR study. The BC2 standard errors provide a greater correction than BC1. While the GEEMAEE macro outputs BC1 and BC2 (as well as uncorrected standard errors) for both mean and correlation estimates, BC1 was recommended for the mean model and BC2 was recommended for the correlation model based on simulation studies. These studies found that sandwich variance estimators for correlation parameters required a greater degree of bias correction than those for marginal mean parameters [1,5–7].

**Power for primary outcome**

Prior to the trial start, the general GEE power calculation method of Rochon [8] for population-averaged models was used to estimate power to detect an intervention log odds ratio δ of a certain size for the primary outcome antibiotic use at first clinic visit. We applied Rochon’s power method for complete and balanced stepped wedge designs for the statistical model in the previous section (but without adjustment for strata, i.e., β_7_ = β_8_ =0) following Li et al. [5]. The STAR study design assumed 15 clusters (villages) transitioning from control to intervention condition in five waves of 3 clusters each and 6 total periods of follow-up as shown in **Figure S2,** except that we assumed an equal number of children not only in each sequence-period but also in each cluster-period. Assuming 12 children recruited per village-month (total sample size of 1080 across the 15 × 6=90 cluster-periods), the study had 87% power (shown in italics in the table) to detect an odds ratio of 0.375 with two-sided α=0.05 GEE Wald tests assuming a zero-slope temporal trend, within-period correlation of 0.10 and between-period correlation of 0.05. Note that, even though a zero temporal trend was assumed, the power calculation adjusts for the estimation of period effects in the marginal mean model. The power calculation assumed an odds ratio of 0.375 for the intervention effect based on an antibiotic use rate of 80% under the control condition and 60% under the intervention condition. The table also shows balanced designs with 13 and 14 participants per cluster-period, respectively. As expected, power increases with increasing total sample size; the design with a total sample size of 1080 was chosen.

The GEE power method of Rochon [8] has been implemented for stepped wedge and other cluster randomized trials designs in a SAS macro CRTFASTGEEPWR [9], which has several options including the capacity to compute power for varying cluster-period sizes. SAS code for power is provided following the table below. The SAS macro CRTFASTGEEPWR, which is at <http://www.bios.unc.edu/~preisser/personal/crtfastgeepwr/>, requires for a logistic regression model that the user convert the intervention odds ratio and the odds of the outcome in the control condition to the log scale. For the STAR study, δ = log(0.375) = -0.98083, p0/(1-p0) = .8/.2=4, and β_j_=log(4)=1.38629 where p0 is the outcome prevalence in the control condition.

It is noteworthy that the SAS macro CRTFASTGEEPWR has the option to calculate power for the intervention effect assuming either *I-p* degrees of freedom, where *I* is the number of clusters and p is the number of regression parameters in the margin mean model, or *I-2* [10,11] with the latter resulting in a non-trivially greater power calculation in cluster randomized trials with a small to moderate *I*. Our power calculation used the macro default value of *I-p,* corresponding to 15 – 7 = 8 degrees of freedom where 7 is the number of regression parameters in the model assuming no strata. Even though the statistical analysis of the STAR data was based on the model that included strata and, therefore, used confidence intervals based on the t-distribution with 15-9=6 degrees of freedom, our power calculation was not necessarily anti-conservative given our choice for the more conservative use of *I-p* over *I-2* as the degrees of freedom.

**References**

1. Preisser JS, Lu B, Qaqish BF. Finite sample adjustments in estimating equations and covariance estimators for intracluster correlations. Stat Med. 2008 Nov 29;27(27):5764–85.

2. Zhang Y, Preisser JS, Li F, Turner EL, Toles M, Rathouz PJ. GEEMAEE: A SAS macro for the analysis of correlated outcomes based on GEE and finite-sample adjustments with application to cluster randomized trials. Comput Methods Programs Biomed. 2023 Mar;230:107362.

3. Kauermann G, Carroll RJ. A Note on the Efficiency of Sandwich Covariance Matrix Estimation. Journal of the American Statistical Association. 2001;96(456):1387–96.

4. Mancl LA, DeRouen TA. A covariance estimator for GEE with improved small-sample properties. Biometrics. 2001 Mar;57(1):126–34.

5. Li F, Turner EL, Preisser JS. Sample size determination for GEE analyses of stepped wedge cluster randomized trials. Biom. 2018 Dec;74(4):1450–8.

6. Teerenstra S, Lu B, Preisser JS, van Achterberg T, Borm GF. Sample size considerations for GEE analyses of three-level cluster randomized trials. Biometrics. 2010 Dec;66(4):1230–7.

7. Zhang Y, Preisser JS, Turner EL, Rathouz PJ, Toles M, Li F. A general method for calculating power for GEE analysis of complete and incomplete stepped wedge cluster randomized trials. Stat Methods Med Res. 2023 Jan;32(1):71–87.

8. Rochon J. Application of GEE procedures for sample size calculations in repeated measures experiments. Stat Med. 1998 Jul 30;17(14):1643–58.

9. Zhang Y, Preisser JS, Li F, Turner EL, Rathouz PJ. CRTFASTGEEPWR: A SAS Macro for Power of Generalized Estimating Equations Analysis of Multi-Period Cluster Randomized Trials with Application to Stepped Wedge Designs. Journal of Statistical Software. 2024 Mar 27;108:1–27.

10. Li F. Design and analysis considerations for cohort stepped wedge cluster randomized trials with a decay correlation structure. Stat Med. 2020 Feb 20;39(4):438–55.

11. Ford WP, Westgate PM. Maintaining the validity of inference in small-sample stepped wedge cluster randomized trials with binary outcomes when using generalized estimating equations. Stat Med. 2020 Sep 20;39(21):2779–92.

**Table.** Pre-planned power for the STAR stepped wedge design with 15 villages (clusters), an intervention odds ratio of 0.375 (corresponding to prevalences of 0.80 and 0.60 in the control and intervention treatment conditions, respectively), a within-period intracluster correlation of 0.10 and a between-period intracluster correlation of 0.05. The scenario with a total sample size of 1080 was chosen among three sample sizes considered.

| Common Cluster-period Size (Total Sample Size) | 12 (1080) | 13 (1170) | 14 (1260) |
| --- | --- | --- | --- |
| Power based on the formula of Li et al. (2018) | ***87*** | 89 | 90 |

**SAS Code for power using macro CRTFASTGEEPWR version 2.04**

* outcome is binary: antibiotics use at initial visit;

* convert control prevalence p0 to the logit scale;

* convert intervention odds ratio to the log scale;

**data** preplanned_numbers;

p0 = **0.80**; /* control outcome proportion */

intercept = log(p0/(**1** - p0));

p1 = **0.60**; /* intervention outcome proportion */

OR_intvn_planned = p1*(**1**-p0)/((**1**-p1)*p0);

delta_planned = log(OR_intvn_planned);

**run**;

**proc** **print** data = preplanned_numbers;

title "Preplanned power: Input regression parameters for logistic model";

**run**;

*Include the SAS macro for fast GEE power;

%include "c:/CRTFASTGEEPWR.v2.04.sas";

*******************************************************************;

** Total n=1080, difference in proportions of 0.20, NE ICC=(0.10,0.05) **;

** p0=0.80, p1=0.60, OR=0.375, delta=log(OR) = -0.98083, intercept= 1.38629*;

** Type I error is alpha = 0.05, equal period effects in no-intercept model*; *******************************************************************;

%***CRTFASTGEEPWR***(alpha=**0.05**, m =%str(J(**5**,**1**,**3**)), corr_type = NE,

alpha1 = **0.10**, alpha2 = **0.05**, intervention_effect_type = AVE,

delta = -**0.98083**, period_effect_type=CAT,

beta_period_effects =%str(J(**6**,**1**,**1.38629**)),

dist = BINARY, phi=**1**, CP_size_matrix = %str(J(**5**,**6**,**12**)),

DesignPattern = ({**0** **1** **1** **1** **1** **1**,

**0** **0** **1** **1** **1** **1**,

**0** **0** **0** **1** **1** **1**,

**0** **0** **0** **0** **1** **1**,

**0** **0** **0** **0** **0** **1**}));
