# Supplementary material for: Point-of-care C-reactive protein measurement by community health workers safely reduces antimicrobial use among children with respiratory illness in rural Uganda: A stepped wedge cluster randomized trial
Source: PLoS Med. 2024 Aug 19;21(8):e1004416. doi: 10.1371/journal.pmed.1004416 (PMC11407643; doi:10.1371/journal.pmed.1004416)
Supplement: S2 Appendix — (PDF) [file pmed.1004416.s013.pdf]

Appendix S2. Supplemental Regulatory Materials

1. Timeline of ethics approvals, trial registration, and participant recruitment
2. Study protocols
3. Ethics board approval letters

Timeline of Regulatory Approvals, Trial Registration, and Participant Recruitment

The referenced protocol versions and ethics approvals are included following the timeline.

- October 9, 2019: Initial study approval by University of North Carolina Institutional Review Board (UNC IRB). The study was approved as a modification to an existing UNC IRB application and initially planned with a pre-post study design.
- June 11, 2020: Initial approval from Mbarara University of Science and Technology Research Ethics Committee (MUST REC). The study was approved as an amendment to an existing MUST REC application and initially planned with a pre-post study design.
- March 25, 2021: Initial study approval by Ugandan National Council on Science and Technology. Study was approved as a renewal and modification of an existing application through July 2024.
- April 9, 2021: Modification approval by UNC IRB – study design change to stepped wedge cluster randomized trial. See Protocol v2.0.
- May 26, 2021: Amendment approval by MUST REC - study design change to stepped wedge cluster randomized trial. See Protocol v2.0.
- November 1, 2021: Start of participant recruitment.
- March 14, 2022: Submission of Trial Registration on ClinicalTrials.gov.
- March 15, 2022: Release of Trial Registration on ClinicalTrials.gov by UNC Protocol Registration and Results System (PRS) Administrator.
- March 21, 2022: UNC IRB renewal with modification - increased sample size. Enrollment proceeded faster than anticipated, so this amendment was to increase the approved sample size. No changes to study design or implementation. See Protocol v3.0.
- March 23, 2022: Registration and Publication of Trial on ClinicalTrials.gov
- April 19, 2022: Amendment approval by MUST REC – increased sample size. Enrollment proceeded faster than anticipated, so this amendment was to increase the approved sample size. No changes to study design or implementation. See Protocol v3.0.

MUST-UNC RESEARCH COLLABORATION

PROTOCOL TITLE: Rapid tests for the evaluation of children with malaria-negative, febrile respiratory illness in rural Uganda.

PROTOCOL VERSION: Version 2.0

PROTOCOL DATE: 1 March 2021

INSTITUTIONS: University of North Carolina at Chapel Hill (UNC)

Mbarara University of Science and Technology (MUST)

FUNDING MECHANISM: University of North Carolina at Chapel Hill
Thrasher Research Fund

PERIOD: 1 May 2019 – 1 May 2022

PRINCIPAL INVESTIGATOR(S)

Edgar Mulogo, PhD, MPH
Associate Professor
Dept. of Community Health
PO Box 1410
Mbarara, Uganda
(+256) 772-433-508
emulogo@must.ac.ug

Emily J Ciccone, MD, MHS
Infectious Diseases Fellow
Division of Infectious Diseases
CB 7030, 130 Mason Farm Rd
Chapel Hill, NC 27599
(+001) 919-966-2537
emily.ciccone@unchealth.unc.edu

Ross M Boyce MD, MSc
Assistant Professor
Division of Infectious Diseases
CB 7030, 130 Mason Farm Rd
Chapel Hill, NC 27599
(+001) 919-966-2537
roboyce@med.unc.edu

Table of Contents

LIST OF ABBREVIATIONS .... - 3 -
PROTOCOL SUMMARY .... - 4 -
1.0 STUDY OBJECTIVE .... - 5 -
2.0 BACKGROUND & SIGNIFICANCE .... - 5 -
3.0 STUDY DESIGN .... - 8 -
4.0 SELECTION AND ENROLLMENT OF PARTICIPANTS .... - 9 -
5.0 STUDY TREATMENT OR INTERVENTION .... - 13 -
6.0 CLINICAL AND LABORATORY INVESTIGATIONS .... - 16 -
7.0 RISK ASSESSMENT & MITIGATION .... - 18 -
8.0 ADVERSE EVENT REPORTING .... - 19 -
9.0 STATISTICAL CONSIDERATIONS .... - 20 -
10.0 STUDY MANAGEMENT .... - 24 -
11.0 CAPACITY BUILDING .... - 26 -
12.0 DISSEMINATION OF RESULTS .... - 27 -
13.0 REFERENCES .... - 28 -

LIST OF ABBREVIATIONS

ARI	Acute respiratory illness
º C	Degrees Celsius
CRP	C-reactive protein
DBS	Dried blood spot
iCCM	Integrated community case management
IMCI	Integrated management of childhood illnesses
ml	Milliliter
mRDT	Malaria rapid diagnostic test
MUST	Mbarara University of Science and Technology
NP	Nasopharyngeal
OPD	Outpatient Department
PCR	Polymerase chain reaction
PCT	Procalcitonin
RDT	Rapid diagnostic test
RSV	Respiratory syncytial virus
UNC	University of North Carolina at Chapel Hill
UNCST	Uganda National Council on Science and Technology
VHW	Village health worker (i.e. community health worker)
WHO	World Health Organization

PROTOCOL SUMMARY

**Protocol Title**	Rapid tests for the evaluation of children with malaria-negative, febrile respiratory illness in rural Uganda.
**Study Design**	Phases 1, 2, and Part 1 of Phase 3: Prospective observational cohort studies<br>Part 2 of Phase 3: Stepped-wedge cluster randomized trial
**Study Population**	Phase 1: Children (age 1 to 10 years) in Kasese Municipality<br>Phase 2: Children ≤ 5 years in Bugoye and Maliba sub-counties of Kasese district<br>Phase 3:<br>- Part 1: Children age 6 months to 5 years in Bugoye sub-county of Kasese district<br>- Part 2: Children ≤ 5 years in Bugoye sub-county
**Number of subjects**	Phase 1: 250 children plus 25 healthy controls<br>Phase 2: 300 children<br>Phase 3: 150 children in Part 1, approximately 900 children and 75 village health workers in Part 2
**Study sites**	Phase 1: Kasese Municipality, Kasese District of Uganda<br>Phase 2: Bugoye and Maliba sub-counties, Kasese District of Uganda<br>Phase 3: Bugoye sub-county, Kasese District of Uganda
**Clinical Samples**	Venous blood, capillary blood by fingerstick, urine, and nasopharyngeal swabs
**Estimated Start of Enrollment**	October 2019
**Estimated Time to Completion**	30 months
**Protocol Duration**	36 months
**Stratification**	Phase 3, Part 2: villages will be stratified according to altitude, distance from Bugoye Health Centre III, and size
**Regimen or Intervention**	Phase 3, Part 2: diagnostic algorithm involving CRP testing used by VHTs to determine need for antibiotics
**Sub-Studies**	Qualitative study of the village health workers who participate in Phase 3, Part 2

1.0 STUDY OBJECTIVE

1.1 Hypothesis

- Our central hypothesis is that commercially-available, rapid, point-of-care diagnostic tests can be used to discriminate bacterial and viral etiologies of febrile acute respiratory illness (ARI), safely reduce unnecessary antibiotic prescriptions, and inform disposition decisions.

1.2 Primary Objectives

- The primary objective of the proposed project is to demonstrate the feasibility and preliminary effectiveness of employing rapid, point-of-care diagnostic tests to improve management of febrile respiratory illness.
- Phase 1 will evaluate the tests at a rural health center with regards to defining the epidemiology of non-malarial febrile ARI and guide antibiotic treatment.
- Phase 2 will evaluate the use of a rapid lactate test by village health workers (VHWs) for determining disposition of children with febrile ARI.
- Phase 3 will test several different rapid tests for C-reactive protein (CRP) against a lab-based gold standard CRP test and then evaluate the use of the best performing rapid CRP test by VHWs for determining need for antibiotic prescription among children with febrile ARI

1.3 Specific Aims

1.3.1 **Aim 1:** Define the epidemiology of non-malaria febrile respiratory illness among children in southwestern Uganda by determining the relative distribution of bacterial and viral causes of ARI.

1A. Quantify the proportion of febrile ARI cases for which an etiology can be identified using commercially-available rapid diagnostic tests for respiratory syncytial virus, influenza, and *Streptococcus pneumoniae*.

1B. Further describe the epidemiology of malaria-negative febrile ARI and host immune response to viral and bacterial etiologies using advanced molecular techniques, specifically next-generation sequencing-based shotgun metagenomics and host gene expression analyses.

1.3.2. **Aim 2:** Among children presenting with febrile ARI to KHC, assess for associations between viral testing, clinical biomarkers, and the following outcomes of interest: duration of symptoms, antibiotic failure defined as need for initiation or change in antibiotic therapy, repeat evaluation at a health facility, acquisition/administration of antibiotics from another source, hospitalization, and death. Using these results, design new or improve existing algorithms for case management of febrile ARI.

2A: Determine the impact of rapid testing on clinic throughput by comparing the number of patients seen and visit duration before and during the study.

1.3.3. **Aim 3.** Evaluate the performance of Integrated Community Case Management (iCCM) algorithms for the identification of lactic acidosis (lactate > 5mmol/L) in children <5 years of age evaluated by VHWs in the Bugoye and Maliba sub-counties.

1.3.4. **Aim 4.** Demonstrate the feasibility and preliminary effectiveness of employing a rapid, point-of-care CRP test to guide antibiotic treatment decisions by VHW for pediatric febrile ARI in rural Uganda.

- 4A: Compare the accuracy, feasibility of use, and cost of three commercially-available, point-of-care, CRP RDTs that can be performed using blood acquired by finger or heel stick.
- 4B: Though a stepped-wedge cluster randomized trial, evaluate the feasibility, preliminary effectiveness, and safety of a CRP RDT by VHW for guiding antibiotic treatment decisions among children with febrile ARI as compared to current iCCM protocols.
- 4C: Compare the frequency with which antibiotics are prescribed for children with malaria-negative, febrile ARI between CHW using a CRP RDT and those using current iCCM protocols.
- 4D: Assess perceptions of antibiotic use and addition of CRP test to iCCM protocols among VHW in Bugoye sub-county.

2.0 BACKGROUND AND SIGNIFICANCE

Global antimicrobial resistance (AMR) is increasing rapidly, posing a major threat to child health (1). A recent review of neonatal bloodstream infections found that >90% of *Klebsiella* and *Escherichia coli* isolates from Africa were resistant to ampicillin and >40% were resistant to gentamicin, both first-line antibiotics for many pediatric conditions (1). One significant driver of AMR is the inappropriate use of antibiotics (2). The World Health Organization (WHO) has prioritized optimization of antimicrobial use as a strategic objective in its Global Action Plan on Antimicrobial Resistance. The development and implementation of effective, rapid, and low-cost diagnostic tools to guide prescribing practices is a key component of achieving this goal (2).

Fever is one of the most common reasons for outpatient evaluation among pediatric patients in Uganda. The wide availability of malaria rapid diagnostic tests (mRDTs), which allow for accurate and timely diagnosis or exclusion of malaria, has increased awareness that a majority of pediatric fever episodes are due to other infectious diseases (3). These malaria-negative children are very frequently treated with antibiotics, yet limited information exists about these non-malaria causes of fever, especially in rural sub-Saharan Africa (4). The data that do exist, however, suggest that a large proportion of fever episodes are caused by self-limited viral infections (5–7). While the fraction of children presenting with febrile illness who ultimately require hospital referral or antibiotics is small, the substantial morbidity and mortality in this population means proper identification of this high-risk group is critical.

Acute respiratory illness (ARI), in particular, is the second leading cause of mortality worldwide in children under five and can be caused by a myriad of bacterial, viral, and fungal pathogens (8). It is also a frequent reason for antibiotic use (9–11). At peripheral health centers and in the home, where many patients are initially evaluated (12), evaluation is often done by non-physician providers, such as lay community health workers, with limited clinical training. Furthermore, diagnosis and management of this condition is often based on clinical symptoms alone. This approach is supported by the WHO's Integrated Management of Childhood Illnesses (IMCI) guidelines, which recommend antibiotic treatment based only on symptoms of cough and fast breathing (13). While there is no definitive gold standard for diagnosing bacterial pneumonia, it is known that clinical symptoms are not specific to a causative organism. In particular, respiratory rate, a key component of the evaluation for pneumonia, is notoriously difficult to accurately

measure and is not predictive of bacterial or radiologically-defined pneumonia. In addition, multiple studies have demonstrated that VHWs often do not appropriately refer sicker patients. This dependence on syndromic diagnosis likely leads to both (a) an overuse of antibiotics, which can cause adverse effects and drive antimicrobial resistance, and (b) an under-recognition of children at high-risk for bacterial infection (10,14,15).

Therefore, there is an urgent need to determine the relative distribution of the causes of non-malaria febrile respiratory illness and improve its case management in rural, resource-limited settings. The development of a method to accurately differentiate bacterial and viral causes is crucial to identify both patients in which antibiotic treatment can be safely withheld and those requiring expedited treatment and/or referral. There is currently limited use of available point-of-care tests for clinical biomarkers, including lactate, C-reactive protein, and procalcitonin, that can help make this distinction and determine the appropriate patient disposition. Elevated lactate has been shown to be associated with mortality among children hospitalized for pneumonia in Uganda (16). In addition, recent studies in Tanzania have demonstrated the potential of leveraging clinical decision algorithms (CDAs) that include point-of-care testing for CRP to reduce antibiotic use without increasing adverse outcomes among febrile children (17,18). However, these and other CDAs based on IMCI guidelines, including Integrated Community Case Management (iCCM), have not yet incorporated testing for specific respiratory pathogens (17–19). In addition, determining need for referral to a higher level facility is based on clinical signs alone or if any testing is included, it is only consists of an mRDT and hemoglobin level (17). To evaluate the potential utility of adding pathogen-specific assays and more extensive clinical biomarker testing to existing algorithms, more information is needed about the epidemiology of ARI and associated biomarker levels.

In our recent prospective study of patients presenting with fever to Kasese Health Center III (KHC) in western Uganda, only 18-32% were mRDT-positive (depending on the season), and 62% of mRDT-negative children presenting with fever reported cough (20). This suggests that non-malarial ARI represents a substantial proportion of visits for fever. In addition, as an exploratory aim, a subset of 73 patients in this study underwent procalcitonin testing, and only 16.4% were positive (>1 ng/mL) suggesting a bacterial etiology. However, all 73 of these patients received antibiotic treatment. Therefore, there is almost certainly a significant proportion of children in with febrile ARI in this region who have an undiagnosed viral infection in whom antibiotics could be avoided.

The overarching scientific goal of this study is to demonstrate the feasibility and preliminary effectiveness of rapid, point-of-care diagnostic tests that can be employed (1) at a rural health center for defining the epidemiology and guiding antibiotic treatment and (2) by village health workers (VHWs) for determining disposition of febrile respiratory illness in pediatric patients. Our hypothesis is that these commercially-available rapid tests can be used to discriminate bacterial and viral etiologies of febrile ARI, safely reduce unnecessary antibiotic prescriptions, and inform disposition decisions.

3.0 STUDY DESIGN

The work described herein seeks to evaluate rapid diagnostic tests (RDTs) to inform management of febrile ARI in two distinct contexts where many ill children are first evaluated – a Level III health center and in the patient's home by a VHW. To do this, the project will consist of two Phases, each consisting of a separate prospective, observational cohort study.

First, we plan conduct a prospective study of pediatric patients presenting to Kasese Health Center III with fever and respiratory symptoms (**Phase 1**). For those that test negative for malaria by mRDT, we will perform rapid testing for common causes of ARI as well as clinical biomarkers that effectively differentiate between viral and bacterial etiology of illness. As this study is observational in nature, no changes to clinical management will be made based on the results of rapid testing done for ARI. Patients will be followed up seven days after initial evaluation to determine outcomes related to their treatment and clinical course. During this phase, we plan to collect additional clinical samples for storage and future advanced molecular testing for etiologic pathogens and host responses from the study participants and a small group of healthy controls. Analysis of samples from healthy participants is necessary to differentiate colonization from true infection in the nasal mucosa and to serve as controls for the host gene expression data. Together, this information will be used to design algorithms for management of malaria-negative ARI that incorporate rapid testing and are feasible in settings with limited laboratory capacity.

In order to also assess the feasibility of equipping VHWs with rapid tests to improve the management of pediatric patients, we plan to conduct a separate prospective cohort study (**Phase 2**). For those children seen by VHWs in Bugoye and Maliba sub-counties of the Kasese District with fever and respiratory symptoms, we will perform a point-of-care lactate measurement. If markedly elevated (>5mmol/L), referral will be recommended given the risk of mortality associated with this degree of lactic acidosis. We will also record if the routine iCCM protocol would have independently recommended referral. Similar to Phase 1, children will be re-assessed at seven days after initial evaluation to determine outcomes related to their clinical course, disposition, and treatment.

Finally, in **Phase 3** of the study, we will focus in particular on the C-reactive protein (CRP) test. We will first compare three commercially-available rapid tests for CRP and one combination malaria/CRP test to a gold-standard lab-based CRP assay among children with fever presenting to Bugoye Health Center (**Part 1**). We will evaluate the tests' accuracy, cost, and ease-of-use. The best-performing test will then be added to routine iCCM protocols for VHWs in Bugoye sub-county to inform antibiotic treatment decisions, similarly to Phase 2, to create a study algorithm for evaluation of children with fever and respiratory symptoms. We will then conduct a stepped-wedge, cluster randomized trial to evaluate the safety of using the study algorithm and its impact on the frequency of antibiotic prescriptions as compared to the routine iCCM protocols (**Part 2**). Children will be re-assessed at seven days after initial evaluation as in Phases 1 and 2. As a sub-study of Part 2, we will conduct surveys and semi-structured interviews with participating VHWs to assess perceptions and feasibility of incorporating point-of-care diagnostics into clinical protocols.

4.0 SELECTION AND ENROLLMENT OF PARTICIPANTS

4.1 Inclusion Criteria

- Phase 1: Age 1-10 years, presentation to Kasese Health Center Outpatient Department (OPD) with fever and respiratory symptoms (documented (temperature > 38°C) or subjective fever in the last seven days, and fast breathing (respiratory rate > 30), cough, and/or hypoxia (oxygen saturation < 90%)).
  - We will also enroll a small group of healthy controls, specifically children age 1-10 years who are not ill (i.e. do not have fever or respiratory symptoms) and are presenting to the KHC OPD either for immunizations or minor traumatic injury.

- Phase 2: Age ≤ 5 years, evaluated by VHT in Bugoye or Maliba for febrile ARI (documented (temperature > 38°C) or subjective fever in the last seven days, and fast breathing (respiratory rate > 30) or cough)

- Phase 3:
  - Part 1: Age 6 months-5 years, presentation to Bugoye Health Center Outpatient Department with fever
  - Part 2: Age 2 months-5 years, evaluated by VHT in Bugoye for febrile ARI (documented (temperature > 38°C) or subjective fever in the last seven days, and fast breathing (respiratory rate > 30) or cough)
    - VHT sub-study: Participation as a VHW in the stepped wedge trial in Part 2

4.2 Exclusion Criteria

- Phase 1: Age <1 or ≥10 years at time of presentation, mRDT-positive

- Phase 2: Age >5 years at time of presentation

- Phase 3: Age >5 years at time of presentation

- For all phases, lack of guardian present to provide consent will also serve as an exclusion criterium.

4.3 Enrollment Procedures

Prior to the start of each phase, study staff will meet with local health partners (clinical staff and administration at KHC for Phase 1, the VHTs for Phase 2 and 3, and clinical staff and administration for Phase 3, Part 1) and community leaders about the aims and methods of the study. There will be an opportunity to ask questions, and together an appropriate timeline for each phase of the study will be determined. Prior to enrollment, all study and laboratory staff will undergo training conducted by experienced MUST and UNC personnel on the required technical skills, ethical conduct of research, and documentation standards. Topics of instruction will include (1) the consent process, (2) the use of the point-of-care tests, (3) follow-up procedures, and (4) record

keeping. The principal and co-investigator will be present during enrollment of the first study participants to audit the study procedure process.

Phase 1

On the specified date, study staff will meet with clinicians to begin study enrollment. All children 1-10 years of age presenting to KHC with febrile ARI as defined above will be screened (**Figure 1**). Patients will first undergo mRDT testing by finger or heel stick as per routine evaluation of children presenting with fever. If positive, the child will not be enrolled in the study and will be treated by clinicians according to local standard of care. If negative, the study coordinator will provide information on study objectives, protocol, and risks and benefits.

Potential healthy control participants will be identified by clinicians and study staff at time of presentation to the OPD or routine immunization clinic. They will be screened using a brief questionnaire asking about any current fever or respiratory symptoms that is included as part of the Initial Assessment Form (**Annex 1**). If found to be asymptomatic, they will be considered eligible for the study and the study coordinator will provide information on study objectives, protocol, and risks and benefits.

For all participants, informed consent will be sought from a parent or guardian. Children aged 8-10 years will also be asked to provide assent to study participation. Those individuals opting not to participate will undergo evaluation and treatment as per clinic protocols.

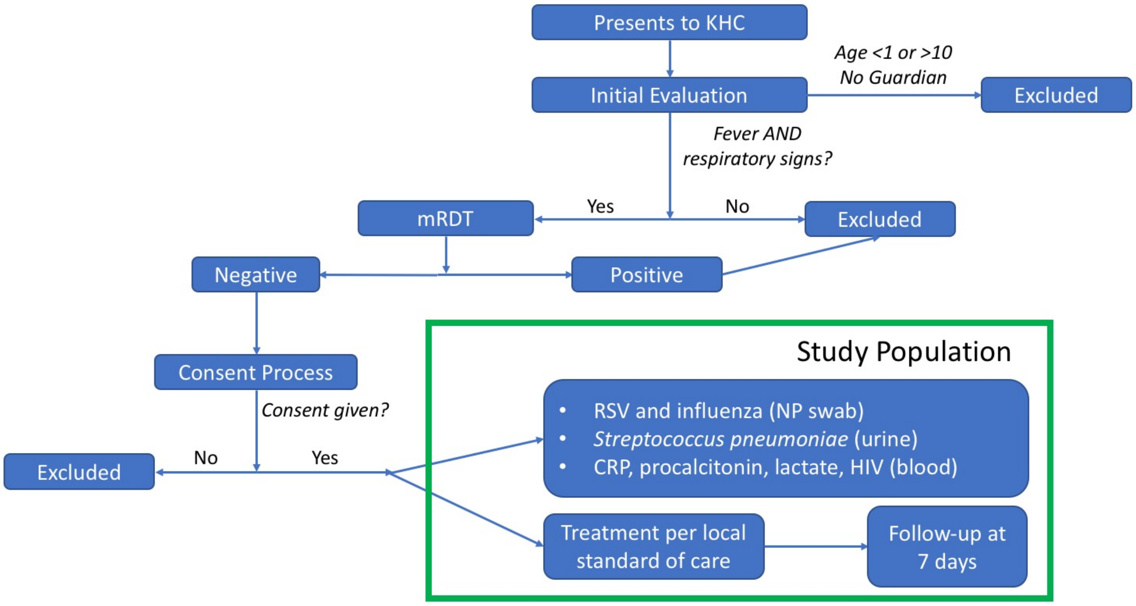

Figure 1. Anticipated Study Flow for Phase 1

Phase 2

Prior to the start of enrollment, two VHWs from each of five villages will undergo a one-week practical training at the local health facility to learn how to use the lactate meter and interpret the results. On the specified study start date, the VHWs will begin screening all children who they evaluate with febrile ARI. The VHWs will provide information on study objectives, protocol, and risks and benefits. Informed consent will be sought from a parent or guardian. Those individuals opting not to participate will undergo evaluation and treatment as per routine iCCM protocols.

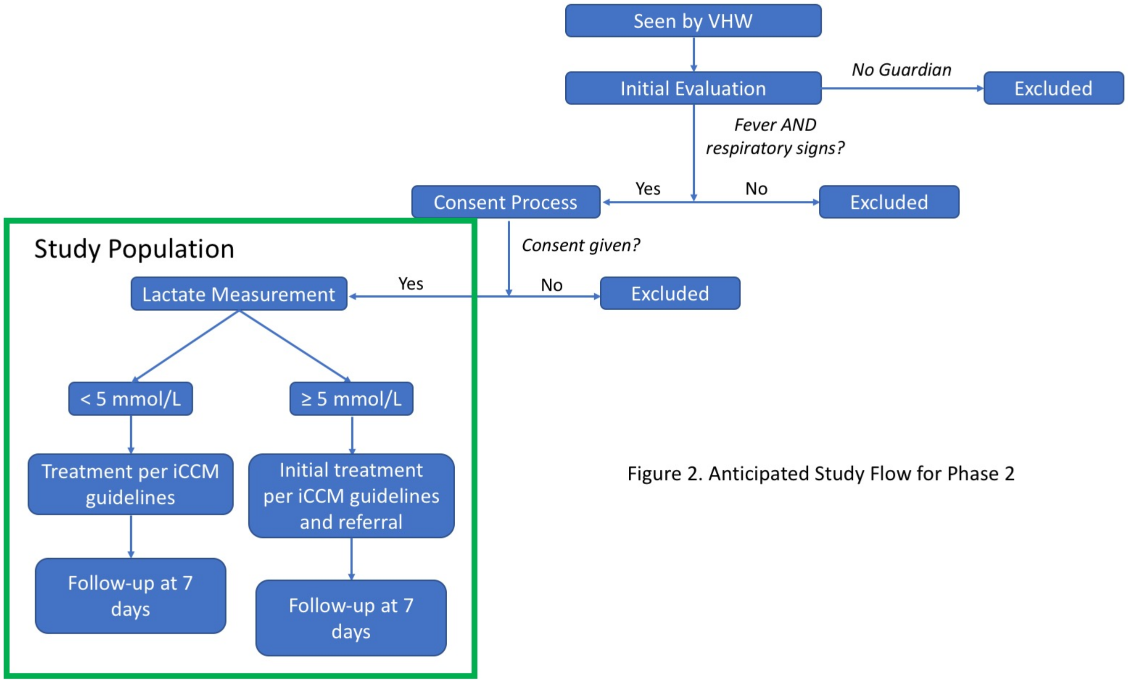

Figure 2. Anticipated Study Flow for Phase 2

Phase 3

All children aged 6 months to 5 years presenting to Bugoye Health Center with fever be eligible for participation in **Part 1 of Phase 3.** Clinicians at BHC will identify eligible children refer them to the study coordinator. The coordinator will then provide information on study objectives, protocol, and risks and benefits to the potential subjects, and informed consent will be sought from a guardian. Those individuals opting not to participate will undergo evaluation and treatment per clinic guidelines.

For **Part 2 of Phase 3**, 5 VHTs from each of 15 villages in Bugoye sub-county will be selected by the study team for participation as care providers in the trial. They will then approach all children evaluated by study VHW in the Bugoye sub-county during the study period who meet the inclusion criteria - documented (temperature > 38°C) or subjective fever in the last seven days AND respiratory signs (fast breathing defined as RR > 30, cough, and/or hypoxia defined as oxygen saturation < 90%) regarding participation in the study. Subjects will be excluded if no guardian is available to provide consent. The VHW will then provide information on study objectives, protocol, and risks and benefits to the potential subjects, and informed consent will be sought from a guardian. Those individuals opting not to participate will undergo evaluation and treatment per iCCM guidelines regardless of whether the village is in a control or intervention period.

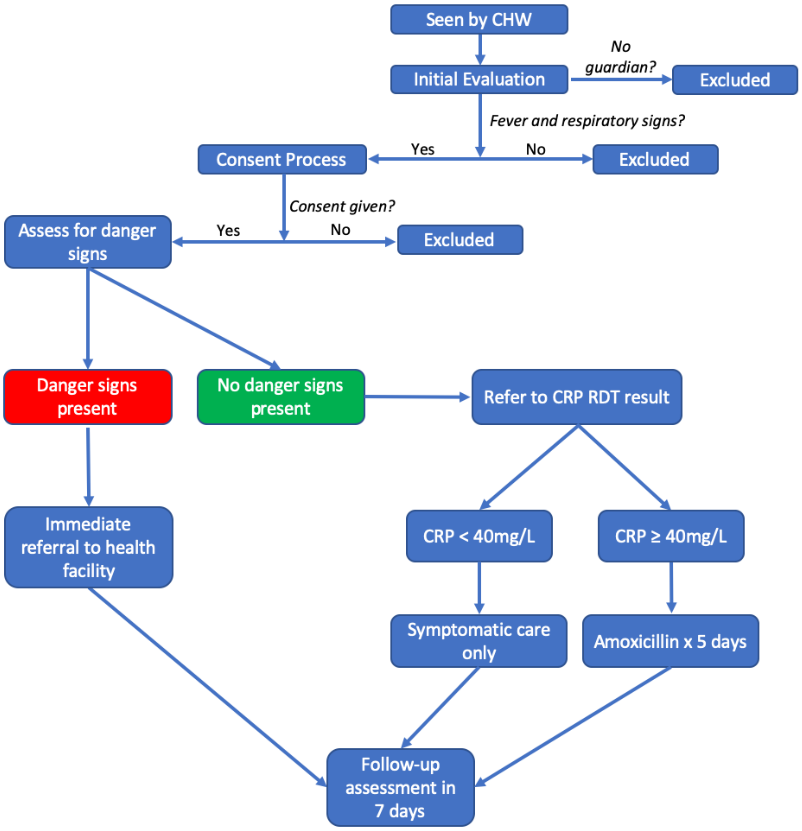

Figure 3. Anticipated Study Flow for Villages in Interventions Periods

5.0 STUDY TREATMENT OR INTERVENTION

In **Phase 1**, the study staff will perform the following activities for enrolled participants:

- Complete an encounter form documenting demographic and clinical information of each participant (**Annex 1**).

- Collect a venous blood sample (~3 mL), urine sample, and three nasopharyngeal swabs from enrolled children.

- Laboratory staff will perform the following rapid testing:
    - Blood sample: HIV, lactate, CRP, PCT
    - Urine sample: *Streptococcus pneumoniae*
    - NP swabs: RSV, influenza

- The remaining blood sample and one of the nasopharyngeal swabs will be stored for future testing, specifically next generation sequencing to identify pathogens not detected by RDTs and analysis of host gene expression in response to respiratory illness.

- Only the mRDT and HIV results will be available to the treating providers. They will administer treatments to the participants per local standards of care.

- The study coordinator will assess all participants (except the healthy controls) seven days after initial evaluation and complete a follow-up form (**Annex 2**).

In **Phase 2**, the study VHTs will perform the following activities:

- Complete an encounter form documenting demographic and clinical information of each participant (**Annex 3**).

- Evaluate participating children according to standard iCCM protocols.

- Measure a lactate level using ~50 microliters of blood obtained by finger or heel stick and document in the monthly register
    - If lactate >5mmol/L, provide initial management per iCCM and refer to nearest health facility or hospital
        - Note on encounter form if iCCM would have independently recommended referral
    - If lactate ≤ 5mmol/L, manage according to iCCM protocols

- Conduct a follow-up visit with participants seven days after initial assessment and complete a follow-up form (**Annex 2**)

In **Phase 3, Part 1**, the study staff will perform the following activities for enrolled patients:

- Complete an encounter form documenting demographic and clinical information of each participant (**Annex 5)**.

- Collect blood by finger or heel stick to perform the following study CRP tests (Actim CRP, Medix Biochemica, Finland, and Standard Q Malaria/CRP Duo, SD Biosensor, Korea (for the first 50 study participants only)) as well as the reference standard CRP test (Afinion CRP, Abbott Diagnostics, USA).

- The clinical providers will not have access to the results of any of the tests and will provide care per local guidelines

In **Phase 3, Part 2,** we will conduct a stepped wedge, cluster randomized trial as follows:

- Fifteen villages will be grouped into three strata based on village size, altitude, and proximity to Bugoye Health Centre.
    - Stratum A - low altitude, proximal, “large” villages (> 115 eligible children seen per year)
        - Bugoye, Ihani, Kanyaminigo, Muramba I, Ndugutu West
    - Stratum B - low altitude, mid-distance, “medium size” villages (90 to 140 eligible children seen per year)
        - Rwakingi 1B, Nyakabugha, Kibirizi, Katooke II, Kirongo
    - Stratum C - high altitude, distal, “small” villages (< 120 eligible children seen per year)
        - Five villages chosen from Ruboni, Mihunga, Kisamba II, Nyangonge, Bunyangoni, Mirimbo

- For the first month of the study, VHTs in ALL villages will:
    - Enroll children with fever and respiratory illness
    - Complete an encounter form documenting demographic and clinical information for each participant (**Annex 6**).
    - Evaluate and treat them according to standard iCCM protocols, including performing an mRDT.

- Each month for the next 5 months, the study team will randomly select a cluster of 3 villages, one village from each strata for transition to the intervention period of the study. Those that are not selected each month will continue study protocols as described above (Figure 4).
    - For the last month of the study, all 15 villages will be in the intervention part of the study.

Figure 4. Stepped Wedge Design

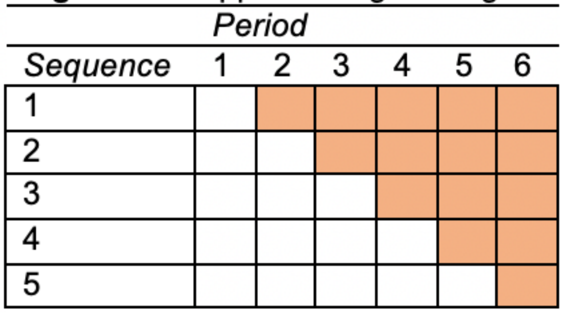

- During the intervention periods, VHTs will:
    - Enroll children with fever and respiratory illness
    - Complete an encounter form documenting demographic and clinical information for each participant (**Annex 6**).
    - Measure a CRP level using ~50 microliters of blood obtained by finger or heel stick and the best-performing CRP RDT from Part 1. Also, perform an mRDT.
    - Treat and manage enrolled children using the study algorithm adapted from standard iCCM protocols that uses the rapid CRP test result to inform antibiotic treatment decisions for those without danger signs (Figure 3).
        - If CRP > 40mg/L, prescribe amoxicillin

    - If CRP ≤ 40mg/L, manage according to iCCM guidelines, but do not prescribe amoxicillin

- For all control and intervention study periods, VHTs will assess for danger signs (severe chest in-drawing, inability to breastfeed or drink, and/or decreased level of consciousness). If present, they will refer patient to the nearest health facility and administer pre-referral management, including antibiotics, per iCCM protocols regardless of CRP result.

- Conduct a follow-up visit with participants seven days after initial assessment and complete a follow-up form (**Annex 7**)

For the **sub-study of Part 2**, we will conduct a qualitative analysis of the intervention period study algorithm as follows:

- All VHTs who participated as care providers in Part 2 will be asked to participate in surveys before the study and semi-structured interviews after the study.

- Surveys and semi-structured interviews will be conducted in the local language, Lukhonjo, by experienced, trained interviewers.

- Topics to be covered include perception of causes of ARI and antibiotic use for its treatment in children, feasibility of addition of CRP testing to clinical evaluation protocols, and perceived impact of testing results on clinical management decisions (Annexes 8, 9, and 10).

- The interviews will be audio-recorded, transcribed, and coded by a study team member for subsequent analysis.

6.0 CLINICAL AND LABORATORY INVESTIGATIONS

6.1 Clinical Investigations

Axillary temperature and pulse oximetry will be measured prior to clinical sample collection. All other clinical investigations will be per standard of care and not dictated by the study.

6.2 Laboratory Investigations

In **Phase 1,** participating children will have ~3mL of blood drawn by a laboratory technician to perform PCT (PCT-Plus, Boditech), CRP (NycoCard CRP, Abbott Diagnostics), and lactate (Lactate Plus, Sports Resource Group, Inc.) measurement as well as rapid HIV testing. Using the urine sample, laboratory technicians will conduct rapid testing for *Streptoccocus pneumoniae* antigen (BinaxNOW, Abbott Diagnostics). Finally, the collected NP swabs will be used for rapid influenza (SD Bioline, Abbott Diagnostics) and RSV (BinaxNOW, Abbott Diagnostics) antigen testing. The remaining blood will be stored in a DNA/RNA Shield Blood Tube (Zymo Diagnostics) and on dried blood spots to preserve nucleic acids for future testing as per Aim 1B.

As part of **Phase 2**, participating children will have ~50 µl of blood drawn by finger or heel stick for point-of-care lactate measurement (Lactate Plus, Sports Resource Group, Inc). The same blood sample will be used for mRDT testing if indicated per iCCM protocols.

In **Part 1 of Phase 3**, participating children will have ~100µL of blood collected from finger or heel stick in order to perform the mRDT and CRP tests (2 RDTs (Actim CRP, Medix Biochemica, Finland; CRP, BTNX, Canada)) and 1 lab-based assay (Afinion CRP, Abbot Diagnostics). Fifty of the study participants will also under to Malaria/CRP combination testing (Standard Q Malaria/CRP Duo, SD Biosensor, South Korea). It may take one to three finger or heel sticks to obtain the necessary blood sample. mRDT testing need to assess eligibility is part of routine standard of care.

In **Part 2 of Phase 3**, participating children will have ~50µL of blood collected from finger or heel stick to perform the mRDT and, if enrolled during an intervention period, the best-performing CRP RDT (as determined in Part 1).

6.3 Clinical Management

Phase 1: As this is an observational study, the clinical management of participants will be primarily dictated by the clinical providers and local standards of care. The only exceptions to that are as follows:

- If an mRDT performed as part of the study protocol is positive, treatment will be administered per local guidelines, and the child will be excluded from the remainder of the study as described above.
- If an HIV test performed as part of the study is positive, the child will be referred to the HIV clinic for further evaluation and treatment.

Phase 2: Treatment and referral plans of participating children will be primarily guided by iCCM protocols (**Annex 4**). Although lactate levels have not been prospectively evaluated in community-

based fever management programs, we will instruct VHWs to refer all children with a lactate ≥5mmol/L, even if not indicated by iCCM protocol, given the high mortality associated with such levels of lactic acidosis in previous studies. However, for the purpose of the analysis, children with lactate levels ≥5mmol/L who did not meet criteria for referral according to iCCM protocols will be classified as discharged from VHW care.

Phase 3:

- Part 1: All CRP testing done as part of this sub-study will be for research purposes only. The clinical management of the participants will be entirely determined by the clinical providers and local standards of care.
- Part 2: During the control periods, treatment and referral plans for participating children will be primarily guided by iCCM protocols (Annex 4), in particular as it relates to assessment and management of children with danger signs or symptoms. During the intervention periods, an mRDT and CRP test will be performed on all enrolled children. The VHW will evaluate all enrolled children for danger signs per iCCM protocols. If present, they will provide pre-referral treatment per iCCM protocols and immediately refer to a health centre. If no danger signs are present, the decision to administer antibiotic treatment will then be dictated by the CRP result. All children who test positive for malaria will be treated with antimalarial medication.

7.0 RISK ASSESSMENT & MITIGATION

7.1 Risks associated with study participation

There may be some minimal discomfort associated with the finger or heel stick, blood draw, and/or nasopharyngeal swab collection. In addition, with the blood draw, there is a small risk of bruising and fainting, and a very rare risk of infection.

There is also the risk of loss of privacy and/or confidentiality concerning medical history should any study documents be compromised or lost.

7.2 Risk Mitigation

To minimize adverse events related to blood draws and finger or heel sticks, all participants shall have samples drawn only by trained study or laboratory staff. Participants shall be informed of all procedures to be done ahead of time. They will be reminded that they have the right to decline any procedure at any time. If an adverse event occurs, affected participants will be immediately assisted to a level III health facility for expedited evaluation.

Universal precautions, including the provision and use of personal protective equipment (PPE), will be required of all study staff when collecting blood. All sharp equipment will be disposed of in appropriate containers. Any staff member who is exposed (example: needle stick) will undergo diagnostic testing (hepatitis, HIV) and receive prophylactic treatment, provided by the study.

To minimize the risk of loss of privacy, patients will be moved to a private area of the clinic or home during informed consent discussions. To minimize the risk of loss of confidentiality. each patient will be assigned a unique identification number on enrolment. Identifying information, specifically name, address, and phone number, will be collected to facilitate follow-up assessments, but once these are complete, the identification number will be the only link between patient information and the study testing results. Once entered into REDCap, the data will be encrypted during transmission and stored on a secure server. Only study staff will have access to the records. All information stored on paper will be scanned and destroyed upon completion of the study.

Household locations may be visually represented in publication, but these will not be linked to individual data and the scale will not be sufficient to locate individual households. Any paper documents, including consent forms, will be scanned and destroyed upon completion of the study.

All laboratory specimens, evaluation forms, reports, and other records that leave the site will be identified by coded number only to maintain participant confidentiality. All records will be kept locked. All computer entry and networking programs will be done with coded numbers only.

8.0 ADVERSE EVENT REPORTING

8.1 Definitions

An adverse event (AE) is any untoward medical occurrence in a patient or clinical investigation participant administered a pharmaceutical product and which does not necessarily have a causal relationship with this treatment. An adverse event can therefore be any unfavorable and unintended sign (including an abnormal laboratory finding), symptom, or disease temporally associated with the use of a medicinal (investigational) product, whether or not related to the medicinal (investigational) product.

An unexpected (unlisted) adverse event is an adverse reaction, the nature or severity of which is not consistent with the applicable product information.

A serious adverse event (SAE) is any untoward medical occurrence that at any dose:

- results in death;
- is life-threatening;
- requires inpatient hospitalization or prolongation of existing hospitalization;
- results in a persistent or significant disability/incapacity;

Important medical events as assessed by medical and scientific judgment may also be considered SAEs by the investigator and will be reported in an expedited fashion.

8.2 Reporting

Given the lack of any medical intervention or investigational pharmaceutical treatment, we do not anticipate any adverse events. In the case of any adverse events related to phlebotomy or NP swab collection, the survey team will immediately coordinate motorized transportation to the nearest level III health facility for expedited evaluation by a clinical officer. If further evaluation is needed, the CO will notify the In-Charge, who will maintain a small transportation fund, to facilitate transportation and onward referral to St. Paul's Level IV Health Center in Kasese or Kilembe Mines Hospital in Kilemebe.

The study coordinator will maintain a log of possible study-related adverse events and will report any unexpected or serious adverse events to the PI, who will communicate the event to the MUST IRC and UNC IRB within 24 hours.

9.0 STATISTICAL CONSIDERATIONS

9.1 General Design Issues:

The proposed work consists of three prospective studies to gain information regarding the feasibility and preliminary effectiveness of using rapid tests to improve diagnosis and management of febrile ARI among pediatric patients. The first two phases of the study, as well as part 1 of the third phase, which will lay the foundations for future interventional studies, are observational. Part 2 of Phase 3 is a stepped-wedge cluster randomized trial where the intervention is an algorithm for evaluation of ARI by VHW that incorporates CRP point-of-care testing.

9.2 Endpoints

Study participation will begin at the time of consent and conclude at the time of the follow-up assessment seven days after the initial assessment. Our outcomes of interest for both Phases 1 and 2 and Part 2 of Phase 3 include the following: frequency of antibiotic prescription at initial assessment, duration of symptoms, antibiotic failure defined as need for initiation or change in antibiotic therapy after the initial assessment, repeat evaluation at a health facility, acquisition/administration of antibiotics from another source (e.g. drug shop), hospitalization, and death.

9.3 Randomization and Stratification:

As this study is observational in nature, there will be no randomization or stratification. All patients meeting inclusion criteria will be approached regarding participation in the study until our target sample size is reached.

9.4 Sample Size and Accrual

Phase 1: Given the exploratory nature of this study, we did not conduct a formal sample size calculation. There are approximately 700 visits to KHC per month by children under 5 years of age. In our recent study, approximately 20-30% of children presenting with fever were mRDT-positive. Of those that were mRDT-negative, 62% reported cough. Assuming a participation rate of 90%, we estimate that it will take 1-2 months to enroll our target 250 participants.

Phase 2: Based on our previous experience at the site, we estimate that each VHW will assess an average of 11 patients each month. Therefore, we have elected to train 10 VHWs to use the lactate plus device, which will result in a total of approximately 330 participants over the three-month period of observation. We will assume a 10% loss to follow up rate, which should leave 300 patients for the primary analysis.

Phase 3: For **Part 1**, assuming a non-inferiority threshold of 5%, a sample size of 150 will achieve > 90% power to demonstrate non-inferiority between each of the 3 CRP only RDTs and the reference standard in regard to identifying those with a CRP ≥ 40mg/L. Our primary outcome of interest is the proportion of children evaluated by VHW for febrile ARI who are prescribed antibiotics (**Part 2**). In previous studies, approximately 80-95% of children evaluated by CHW for fast breathing received antibiotics. We anticipate that this proportion will be similar, if not

higher, among children who also have fever, although patients with fever and cough in the absence of fast breathing may be prescribed antibiotics less frequently. Therefore, we conservatively estimate that 80% of patients evaluated by CHW for fever and any respiratory sign currently receive antibiotics. The GEE power calculation method of Rochon (1998) for population-averaged models is applied to the binary outcome antibiotic use for the stepped wedge design in Figure 4. Assuming ten children recruited per village-month, the power to detect a difference in proportions of 0.20 with two-sided α=0.05 GEE Wald tests will be greater than 80% for a wide range of plausible intraclass correlations (**Table 1**). Li et al. have shown that the analytical power derived by this method agrees well with simulated power for as few as eight clusters when data are analyzed using the bias-adjusted GEE procedure reference above (21). Therefore, our goal is to enroll 10 children per month per village, or a total of 900 children.

Table 1. Statistical Power for two-sided, α=0.05 significance tests in the marginal model analysis of intervention effect in a stepped wedge cross-sectional design assuming three villages per treatment sequence.

		Number of children per village/month		
α_0	α_1	8	**10**	12
0.025	0.0125	93	**96**	98
0.05	0.025	88	**93**	96
0.10	0.05	80	**86**	89
0.15	0.075	73	**79**	82

[1]The baseline (control) rate of antibiotic prescribing is assumed to be 0.80 and the rate under the intervention condition is 0.60, corresponding to an odds ratio (e^{δ}) of 0.375; we assume a logistic model with β_j = log(0.8/0.2).

9.5 Monitoring

Because this study is observational in nature and does not include any therapeutic or pharmacologic intervention, no Data and Safety Monitoring Board (DSMB) is planned.

9.6 Analyses

Phase 1

We will use the results of the study at KHC for the following analyses:

- Estimate the proportion of cases of malaria-negative, febrile respiratory illness for which an etiology can be determined using commercially available point-of-care rapid diagnostic tests.

- Determine the proportion of children presenting with malaria-negative, febrile ARI who test positive for RSV and influenza.

- Determine the proportion of children presenting with malaria-negative, febrile ARI who pneumococcal pneumonia (based on positive *Streptococcus pneumoniae* urine antigen testing AND an elevated CRP).

- Using next-generation sequencing-based shotgun metagenomics to evaluate the nasopharyngeal sample and blood for causes of febrile ARI that are not detectable by rapid testing.

- Assess for differences in host gene expression (1) between participants diagnosed with viral versus bacterial etiology of febrile ARI, (2) between healthy controls and participants diagnosed with viral infection, and (3) between healthy controls and participants diagnosed with bacterial infection.

- Evaluate for any associations between rapid test and clinical biomarker results and our outcomes of interest: duration of symptoms, antibiotic failure defined as need for initiation or change in antibiotic therapy after the initial assessment, repeat evaluation at a health facility, acquisition/administration of antibiotics from another source (e.g. drug shop), hospitalization, and death.

- Measure the impact of rapid testing on clinic throughput by comparing the number of patients seen and visit duration before and during the study

Phase 2:

We will use the results of the study involving VHWs to perform the following analyses:

- Quantify the prevalence of lactic acidosis among children presenting to VHWs with febrile ARI in the Bugoye and Maliba sub-counties.

- Determine the sensitivity, specificity, negative predictive value, and positive predictive value of iCCM protocols for identifying children with an elevated lactate.

- Evaluate for associations between lactic acidosis and our outcomes of interest: duration of symptoms, antibiotic failure defined as need for initiation or change in antibiotic therapy after the initial assessment, repeat evaluation at a health facility, acquisition/administration of antibiotics from another source (e.g. drug shop), hospitalization, and death.

Phase 3: We will use the results of the CRP study for the following analyses

- Part 1
    - Determine the accuracy of each CRP RDT by calculating the sensitivity and specificity as compared to the gold standard, lab-based CRP assay
    - Assess feasibility of RDT use by calculating the median time to perform each test
    - Select the best-performing RDT by determining which one maximizes accuracy, feasibility and cost.

- Part 2
    - Calculate the prevalence of elevated CRP (>40mg/L) among children presenting to VHTs with fever and respiratory symptoms
    - Assess for differences in frequency of antibiotic prescription at initial VHW evaluation and symptom resolution at follow-up visit between CRP-guided and routine iCCM protocols
    - Evaluate for any associations between CRP RDT results and our outcomes of interest: duration of symptoms, antibiotic failure defined as need for initiation or change in

antibiotic therapy after the initial assessment, repeat evaluation at a health facility, acquisition/administration of antibiotics from another source (e.g. drug shop), hospitalization, and death.

- The audio recordings of the semi-structured interviews will be transcribed verbatim. Qualitative data will be analyzed following an inductive, grounded theory approach. The codes will be refined and sharpened using the data collected, with any disagreements resolved through consensus of the study team, to produce a final set of codes that will be used to re-code all the transcripts.

10.0 STUDY MANAGEMENT

10.1 Institutional Review Board (IRB) Review and Informed Consent

This protocol and the informed consent documents and any subsequent modifications (amendments) will be reviewed and approved by the Mbarara University of Science and Technology REC, the Uganda National Council of Science and Technology (UNCST), and the UNC IRB. It is expected that the IRB will have the proper representation and function in accordance with federally mandated regulations.

In obtaining and documenting informed consent, the investigator will comply with the applicable regulatory requirement(s), and should adhere to Good Clinical Practice (GCP) and to ethical principles that have their origin in the Declaration of Helsinki.

Before recruitment and enrollment onto this study, the participant will be given a full explanation of the study and will be given the opportunity to review the consent form(s). Each consent form will include all the relevant elements currently required by the local regulations, which include elements such as the purpose of the study, the procedures to be followed, and the risks and benefits of participation. Once this essential information has been provided to the participant and the investigator is assured that the participant understands the implications of participating in the study, the participant will be asked to give consent to participate in the study by signing a REC/IRB-approved consent form. During Part 2 of Phase 3, both the VHW implementing the study and the children they enroll will complete separate consent forms as both are considered research subjects.

Prior to a participant's participation in the trial, the written informed consent form(s) will be signed and personally dated by the participant or the participant's legally authorized representative (parent or guardian), and by the person who conducted the informed consent discussion.

10.2 Registration Procedures

All participants will be identified by a unique identifier and not by name. Identifying information including name, phone number, and household location will be recorded to facilitate follow-up assessments at seven days after enrollment. Once this evaluation is complete, the identification number will be the only link between patient information and the study testing results.

10.3 Participant Incentives

In Phase 1, participants, including healthy controls, will receive the following for participating in this study:

- 5,000 shillings to cover the cost of transportation to the clinic
- A bar of soap for washing
- 1kg of sugar
- A cookie and a soda for the child.

There are no incentives for participation in Phase 2; there are no additional procedures anticipated as children with fever undergo mRDT testing by finger or heel stick per iCCM protocols.

In Phase 3, the children/caregiver will receive the following for participating in the study:

- A bar of soap for washing
- A cookie and a soda for the child.

In Phase 3, Part 2, the participating VHTs will receive 20,000UGX per month for their participation and to reimburse them for the cost of transportation to bring their data collection forms to the VHW office in Bugoye (this is the current system for VHW program documents). Those who complete the end of study semi-structured interview will also receive a snack and a drink during the semi-structured interview.

10.4 Criteria for Discontinuation

If, at any time the constraints of this protocol are detrimental to the participant's health or believed not to be in the patient’s best interests by study staff, even if not previously addressed, and/or the participant no longer wishes to continue study treatment, we will remove the participant from further consideration.

Furthermore, if an adequate specimen is not obtained after three finger/heel sticks or blood draw attempts, no further attempts will be made and the individual will be excluded.

10.5 Deviations from the Protocol

The investigator may implement a deviation from, or a change of, the protocol to eliminate an immediate hazard(s) to study participants without prior REC/IRB approval/favorable opinion. As soon as possible, the implemented deviation or change, the reasons for it, and, if appropriate, the proposed protocol amendment(s) should be submitted to (i) REC/IRB for review and approval, (ii) the sponsor for agreement and, if required and (iii) the regulatory authority(ies).

10.6 Adherence to the Protocol

Except for an emergency situation in which proper care for the protection, safety, and well-being of the study participant requires alternative treatment, the study shall be conducted exactly as described in the approved protocol. Any deviation from the protocol must have prior approval by the Principal Investigator and must be recorded and explained.

10.7 Amendments to the Protocol

Should amendments to the protocol be required, the amendments will be originated and documented by the Principal and Co-investigators. The written amendment will be sent to investigators and must be submitted to the IRB at the investigator's site for approval. It should also be noted that when an amendment to the protocol substantially alters the study design or the potential risk to the participant, a revised consent form might be required.

10.8 Record & Sample Retention

Study documentation will include all source documents and regulatory documents (e.g., protocol and amendments, IRB correspondence and approval, signed participant consent forms). Source documents will include all recordings of observations or notations of clinical activities and all reports and records necessary for the evaluation and reconstruction of the clinical research study.

The proposed study does include the collection and storage of blood samples in tubes and on filter paper and NP swabs in tubes for analysis at a later date. These samples will be identified only by the coded ID. Samples will be stored for the genomic analyses mentioned above as part of Aim 1B. All samples will be stored in Uganda at Epicentre Research Base in Mbarara until testing occurs. Any additional utilization of these samples, however, will require a modification to the current REC/IRB application prior to initiation.

10.9 Participant Confidentiality

All laboratory specimens, evaluation forms, reports, and other records that leave the site will be identified by coded number only to maintain participant confidentiality. All records will be kept locked. All computer entry and networking programs will be done with coded numbers only. Clinical information will not be released without written permission of the participant.

11.0 CAPACITY BUILDING

11.1 Students & Trainees

Pending interest, we will engage a Masters student from the Department of Community Health as a research assistant for this study. Responsibilities would include assisting with supervision of enrollment, data entry and analysis, and manuscript preparation. Other projects that involve further analysis of the obtained data could also be made available to other students and trainees.

11.2 Investigators & Study Staff

The proposed study will employ electronic tablets for data collection in RedCap (www.redcap.unc.edu). Field staff will be trained to use this application and will receive certificates at the completion of the study. In addition, laboratory staff will gain new skills as it relates to conducting a broader array of rapid diagnostic tests.

11.3 Local Officials & Infrastructure

The proposed study will provide important information to leaders and policymakers at the village, sub-county, and district level. Enhanced knowledge regarding the epidemiology of acute respiratory illness in the area could inform clinical practice and policies. In addition, understanding how well iCCM protocols identify the sickest children could help shape future improvements of those guidelines.

12.0 DISSEMINATION OF RESULTS

12.1 Data Sharing

Data ownership will be jointly shared between Drs. Mulogo and Boyce. Based on the background and history of collaboration between UNC and collaborators in Uganda, we do not anticipate problems in resolving conflicts over data access or use. However, we will develop procedures for disagreements if they arise. We will first attempt to resolve the conflict within the group. This includes discussions about any issue, alternative approaches to a solution. If unsuccessful, the PIs and key personnel will escalate the problem to an agreed upon arbiter, who will be asked to adjudicate within a two-week period (or sooner if urgent).

12.2 Dissemination

The results of the study will be shared with the community upon completion, through meetings with local health partners and the district leadership. A poster, in both English and Lukhonjo, outlining the study results will be placed at Kasese and Bugoye Health Centers. Representatives from the office of the respective District Health Offices (DHO), including the District Laboratory Officers, will be updated regularly on the progress of the study. The final results will be shared with the DHOs prior to any presentation and/or publication.

From the study, we anticipate at least three publications in an international scientific journal, such as the *American Journal of Tropical Medicine and Hygiene*. The results may also be presented at various conferences pending acceptance of any abstract or poster. All participating team members will be acknowledged, and those contributing to the analysis and/or manuscript preparation will be listed as a co-author.

13.0 REFERENCES

1. Le Doare K, Bielicki J, Heath PT, Sharland M. Systematic Review of Antibiotic Resistance Rates Among Gram-Negative Bacteria in Children With Sepsis in Resource-Limited Countries. Journal of the Pediatric Infectious Diseases Society. 2015 Mar;4(1):11–20.

2. World Health Organization. Global Action Plan on Antimicrobial Resistance [Internet]. Geneva, Switzerland: World Health Organization; 2015. Available from: https://apps.who.int/iris/bitstream/handle/10665/193736/9789241509763_eng.pdf

3. D'Acremont V, Lengeler C, Genton B. Reduction in the proportion of fevers associated with Plasmodium falciparum parasitaemia in Africa: a systematic review. Malaria Journal. 2010;9(1):240.

4. Maze MJ, Bassat Q, Feasey NA, Mandomando I, Musicha P, Crump JA. The epidemiology of febrile illness in sub-Saharan Africa: implications for diagnosis and management. Clinical Microbiology and Infection. 2018 Aug 1;24(8):808–14.

5. Guillebaud J, Bernardson B, Randriambolamanantsoa TH, Randrianasolo L, Randriamampionona JL, Marino CA, et al. Study on causes of fever in primary healthcare center uncovers pathogens of public health concern in Madagascar. Bailey RL, editor. PLOS Neglected Tropical Diseases. 2018 Jul 16;12(7):e0006642.

6. D'Acremont V, Kilowoko M, Kyungu E, Philipina S, Sangu W, Kahama-Maro J, et al. Beyond Malaria — Causes of Fever in Outpatient Tanzanian Children. New England Journal of Medicine. 2014 Feb 27;370(9):809–17.

7. Elfving K, Shakely D, Andersson M, Baltzell K, Ali AS, Bachelard M, et al. Acute Uncomplicated Febrile Illness in Children Aged 2-59 months in Zanzibar – Aetiologies, Antibiotic Treatment and Outcome. Lin B, editor. PLOS ONE. 2016 Jan 28;11(1):e0146054.

8. World Health Organization. Maternal, Newborn, Child and Adolescent Health: Causes of Death Among Children [Internet]. [cited 2018 Aug 2]. Available from: http://www.who.int/maternal_child_adolescent/data/causes-death-children/en/

9. Ocan M, Aono M, Bukirwa C, Luyinda E, Ochwo C, Nsambu E, et al. Medicine use practices in management of symptoms of acute upper respiratory tract infections in children (≤12 years) in Kampala city, Uganda. BMC Public Health [Internet]. 2017 Dec [cited 2018 Aug 2];17(1). Available from: http://bmcpublichealth.biomedcentral.com/articles/10.1186/s12889-017-4770-1

10. Risk R, Naismith H, Burnett A, Moore SE, Cham M, Unger S. Rational prescribing in paediatrics in a resource-limited setting. Archives of Disease in Childhood. 2013 Jul 1;98(7):503–9.

11. Ndhlovu M, Nkhama E, Miller JM, Hamer DH. Antibiotic prescribing practices for patients with fever in the transition from presumptive treatment of malaria to 'confirm and treat' in Zambia: a cross-sectional study. Tropical Medicine & International Health. 2015 Dec;20(12):1696–706.

12. Uganda Bureau of Statistics (UBOS) and ICF International. Uganda Malaria Indicator Survey 2014-2015. Kampala, Uganda and Rockville, Maryland: UBOS and ICF International; 2015.

13. World Health Organization, Department of Maternal N Child and Adolescent Health, World Health Organization. Revised WHO classification and treatment of pneumonia in children at health facilities: evidence summaries. [Internet]. 2014 [cited 2018 Oct 17]. Available from: http://apps.who.int/iris/bitstream/10665/137319/1/9789241507813_eng.pdf?ua=1

14. Mukanga D, Konaté AT, Cousens S, Amenga-Etego L, Pagnoni F, Tibenderana JK, et al. Integrated Community Case Management of Fever in Children under Five Using Rapid Diagnostic Tests and Respiratory Rate Counting: A Multi-Country Cluster Randomized Trial. The American Journal of Tropical Medicine and Hygiene. 2012 Nov 7;87(5_Suppl):21–9.

15. Means AR, Weaver MR, Burnett SM, Mbonye MK, Naikoba S, McClelland RS. Correlates of Inappropriate Prescribing of Antibiotics to Patients with Malaria in Uganda. Diemert DJ, editor. PLoS ONE. 2014 Feb 28;9(2):e90179.

16. Gunaratnam LC, Conroy AL, Ma C, Namasopo S, Hawkes MT, Ericson A, et al. Handheld Point-of-Care Lactate Measurement at Admission Predicts Mortality in Ugandan Children Hospitalized with Pneumonia: A Prospective Cohort Study. The American Journal of Tropical Medicine and Hygiene [Internet]. 2018 Nov 5 [cited 2019 Jan 4]; Available from: http://www.ajtmh.org/content/journals/10.4269/ajtmh.18-0344

17. Keitel K, Kagoro F, Samaka J, Masimba J, Said Z, Temba H, et al. A novel electronic algorithm using host biomarker point-of-care tests for the management of febrile illnesses in Tanzanian children (e-POCT): A randomized, controlled non-inferiority trial. Tumwine JK, editor. PLOS Medicine. 2017 Oct 23;14(10):e1002411.

18. Rambaud-Althaus C, Shao AF, Kahama-Maro J, Genton B, d'Acremont V. Managing the Sick Child in the Era of Declining Malaria Transmission: Development of ALMANACH, an Electronic Algorithm for Appropriate Use of Antimicrobials. Borrmann S, editor. PLOS ONE. 2015 Jul 10;10(7):e0127674.

19. Keitel K, D'Acremont V. Electronic clinical decision algorithms for the integrated primary care management of febrile children in low-resource settings: review of existing tools. Clinical Microbiology and Infection. 2018 Aug;24(8):845–55.

20. Boyce RM. Personal Communication: Demographic and clinical characteristics of children presenting to the Kasese Health Center in May 2018. 2018.

21. Li F, Turner EL, Preisser JS. Sample size determination for GEE analyses of stepped wedge cluster randomized trials. Biom. 2018 Dec;74(4):1450–8.

MUST-UNC RESEARCH COLLABORATION

PROTOCOL TITLE: Rapid tests for the evaluation of children with malaria-negative, febrile respiratory illness in rural Uganda.

PROTOCOL VERSION: Version 3.0

PROTOCOL DATE: 1 March 2022

INSTITUTIONS: University of North Carolina at Chapel Hill (UNC)

Mbarara University of Science and Technology (MUST)

FUNDING MECHANISM: University of North Carolina at Chapel Hill
Thrasher Research Fund

PERIOD: 1 May 2019 – 31 December 2022

PRINCIPAL INVESTIGATOR(S)

Edgar Mulogo, PhD, MPH
Associate Professor
Dept. of Community Health
PO Box 1410
Mbarara, Uganda
(+256) 772-433-508
emulogo@must.ac.ug

Emily J Ciccone, MD, MHS
Infectious Diseases Fellow
Division of Infectious Diseases
CB 7030, 130 Mason Farm Rd
Chapel Hill, NC 27599
(+001) 919-966-2537
emily.ciccone@unchealth.unc.edu

Ross M Boyce MD, MSc
Assistant Professor
Division of Infectious Diseases
CB 7030, 130 Mason Farm Rd
Chapel Hill, NC 27599
(+001) 919-966-2537
roboyce@med.unc.edu

Table of Contents

LIST OF ABBREVIATIONS ..... - 3 -
PROTOCOL SUMMARY ..... - 4 -
1.0 STUDY OBJECTIVE ..... - 5 -
2.0 BACKGROUND & SIGNIFICANCE ..... - 5 -
3.0 STUDY DESIGN ..... - 8 -
4.0 SELECTION AND ENROLLMENT OF PARTICIPANTS ..... - 9 -
5.0 STUDY TREATMENT OR INTERVENTION ..... - 13 -
6.0 CLINICAL AND LABORATORY INVESTIGATIONS ..... - 16 -
7.0 RISK ASSESSMENT & MITIGATION ..... - 18 -
8.0 ADVERSE EVENT REPORTING ..... - 19 -
9.0 STATISTICAL CONSIDERATIONS ..... - 20 -
10.0 STUDY MANAGEMENT ..... - 24 -
11.0 CAPACITY BUILDING ..... - 26 -
12.0 DISSEMINATION OF RESULTS ..... - 27 -
13.0 REFERENCES ..... - 28 -

LIST OF ABBREVIATIONS

ARI	Acute respiratory illness
º C	Degrees Celsius
CRP	C-reactive protein
DBS	Dried blood spot
iCCM	Integrated community case management
IMCI	Integrated management of childhood illnesses
ml	Milliliter
mRDT	Malaria rapid diagnostic test
MUST	Mbarara University of Science and Technology
NP	Nasopharyngeal
OPD	Outpatient Department
PCR	Polymerase chain reaction
PCT	Procalcitonin
RDT	Rapid diagnostic test
RSV	Respiratory syncytial virus
UNC	University of North Carolina at Chapel Hill
UNCST	Uganda National Council on Science and Technology
VHW	Village health worker (i.e. community health worker)
WHO	World Health Organization

PROTOCOL SUMMARY

**Protocol Title**	Rapid tests for the evaluation of children with malaria-negative, febrile respiratory illness in rural Uganda.
**Study Design**	Phases 1, 2, and Part 1 of Phase 3: Prospective observational cohort studies<br>Part 2 of Phase 3: Stepped-wedge cluster randomized trial
**Study Population**	Phase 1: Children (age 1 to 10 years) in Kasese Municipality<br>Phase 2: Children ≤ 5 years in Bugoye and Maliba sub-counties of Kasese district<br>Phase 3:<br>- Part 1: Children age 6 months to 5 years in Bugoye sub-county of Kasese district<br>- Part 2: Children ≤ 5 years in Bugoye sub-county
**Number of subjects**	Phase 1: 250 children plus 25 healthy controls<br>Phase 2: 300 children<br>Phase 3: 150 children in Part 1, approximately 1,375 children and 75 village health workers in Part 2
**Study sites**	Phase 1: Kasese Municipality, Kasese District of Uganda<br>Phase 2: Bugoye and Maliba sub-counties, Kasese District of Uganda<br>Phase 3: Bugoye sub-county, Kasese District of Uganda
**Clinical Samples**	Venous blood, capillary blood by fingerstick, urine, and nasopharyngeal swabs
**Estimated Start of Enrollment**	October 2019
**Estimated Time to Completion**	30 months
**Protocol Duration**	36 months
**Stratification**	Phase 3, Part 2: villages will be stratified according to altitude, distance from Bugoye Health Centre III, and size
**Regimen or Intervention**	Phase 3, Part 2: diagnostic algorithm involving CRP testing used by VHTs to determine need for antibiotics
**Sub-Studies**	Qualitative study of the village health workers who participate in Phase 3, Part 2

1.0 STUDY OBJECTIVE

1.1 Hypothesis

- Our central hypothesis is that commercially-available, rapid, point-of-care diagnostic tests can be used to discriminate bacterial and viral etiologies of febrile acute respiratory illness (ARI), safely reduce unnecessary antibiotic prescriptions, and inform disposition decisions.

1.2 Primary Objectives

- The primary objective of the proposed project is to demonstrate the feasibility and preliminary effectiveness of employing rapid, point-of-care diagnostic tests to improve management of febrile respiratory illness.
- Phase 1 will evaluate the tests at a rural health center with regards to defining the epidemiology of non-malarial febrile ARI and guide antibiotic treatment.
- Phase 2 will evaluate the use of a rapid lactate test by village health workers (VHWs) for determining disposition of children with febrile ARI.
- Phase 3 will test several different rapid tests for C-reactive protein (CRP) against a lab-based gold standard CRP test and then evaluate the use of the best performing rapid CRP test by VHWs for determining need for antibiotic prescription among children with febrile ARI

1.3 Specific Aims

1.3.1 **Aim 1:** Define the epidemiology of non-malaria febrile respiratory illness among children in southwestern Uganda by determining the relative distribution of bacterial and viral causes of ARI.

1A. Quantify the proportion of febrile ARI cases for which an etiology can be identified using commercially-available rapid diagnostic tests for respiratory syncytial virus, influenza, and *Streptococcus pneumoniae*.

1B. Further describe the epidemiology of malaria-negative febrile ARI and host immune response to viral and bacterial etiologies using advanced molecular techniques, specifically next-generation sequencing-based shotgun metagenomics and host gene expression analyses.

1.3.2. **Aim 2:** Among children presenting with febrile ARI to KHC, assess for associations between viral testing, clinical biomarkers, and the following outcomes of interest: duration of symptoms, antibiotic failure defined as need for initiation or change in antibiotic therapy, repeat evaluation at a health facility, acquisition/administration of antibiotics from another source, hospitalization, and death. Using these results, design new or improve existing algorithms for case management of febrile ARI.

2A: Determine the impact of rapid testing on clinic throughput by comparing the number of patients seen and visit duration before and during the study.

1.3.3. **Aim 3.** Evaluate the performance of Integrated Community Case Management (iCCM) algorithms for the identification of lactic acidosis (lactate > 5mmol/L) in children <5 years of age evaluated by VHWs in the Bugoye and Maliba sub-counties.

1.3.4. **Aim 4.** Demonstrate the feasibility and preliminary effectiveness of employing a rapid, point-of-care CRP test to guide antibiotic treatment decisions by VHW for pediatric febrile ARI in rural Uganda.

- 4A: Compare the accuracy, feasibility of use, and cost of three commercially-available, point-of-care, CRP RDTs that can be performed using blood acquired by finger or heel stick.
- 4B: Though a stepped-wedge cluster randomized trial, evaluate the feasibility, preliminary effectiveness, and safety of a CRP RDT by VHW for guiding antibiotic treatment decisions among children with febrile ARI as compared to current iCCM protocols.
- 4C: Compare the frequency with which antibiotics are prescribed for children with malaria-negative, febrile ARI between CHW using a CRP RDT and those using current iCCM protocols.
- 4D: Assess perceptions of antibiotic use and addition of CRP test to iCCM protocols among VHW in Bugoye sub-county.

2.0 BACKGROUND AND SIGNIFICANCE

Global antimicrobial resistance (AMR) is increasing rapidly, posing a major threat to child health (1). A recent review of neonatal bloodstream infections found that >90% of *Klebsiella* and *Escherichia coli* isolates from Africa were resistant to ampicillin and >40% were resistant to gentamicin, both first-line antibiotics for many pediatric conditions (1). One significant driver of AMR is the inappropriate use of antibiotics (2). The World Health Organization (WHO) has prioritized optimization of antimicrobial use as a strategic objective in its Global Action Plan on Antimicrobial Resistance. The development and implementation of effective, rapid, and low-cost diagnostic tools to guide prescribing practices is a key component of achieving this goal (2).

Fever is one of the most common reasons for outpatient evaluation among pediatric patients in Uganda. The wide availability of malaria rapid diagnostic tests (mRDTs), which allow for accurate and timely diagnosis or exclusion of malaria, has increased awareness that a majority of pediatric fever episodes are due to other infectious diseases (3). These malaria-negative children are very frequently treated with antibiotics, yet limited information exists about these non-malaria causes of fever, especially in rural sub-Saharan Africa (4). The data that do exist, however, suggest that a large proportion of fever episodes are caused by self-limited viral infections (5–7). While the fraction of children presenting with febrile illness who ultimately require hospital referral or antibiotics is small, the substantial morbidity and mortality in this population means proper identification of this high-risk group is critical.

Acute respiratory illness (ARI), in particular, is the second leading cause of mortality worldwide in children under five and can be caused by a myriad of bacterial, viral, and fungal pathogens (8). It is also a frequent reason for antibiotic use (9–11). At peripheral health centers and in the home, where many patients are initially evaluated (12), evaluation is often done by non-physician providers, such as lay community health workers, with limited clinical training. Furthermore, diagnosis and management of this condition is often based on clinical symptoms alone. This approach is supported by the WHO's Integrated Management of Childhood Illnesses (IMCI) guidelines, which recommend antibiotic treatment based only on symptoms of cough and fast breathing (13). While there is no definitive gold standard for diagnosing bacterial pneumonia, it is known that clinical symptoms are not specific to a causative organism. In particular, respiratory rate, a key component of the evaluation for pneumonia, is notoriously difficult to accurately

measure and is not predictive of bacterial or radiologically-defined pneumonia. In addition, multiple studies have demonstrated that VHWs often do not appropriately refer sicker patients. This dependence on syndromic diagnosis likely leads to both (a) an overuse of antibiotics, which can cause adverse effects and drive antimicrobial resistance, and (b) an under-recognition of children at high-risk for bacterial infection (10,14,15).

Therefore, there is an urgent need to determine the relative distribution of the causes of non-malaria febrile respiratory illness and improve its case management in rural, resource-limited settings. The development of a method to accurately differentiate bacterial and viral causes is crucial to identify both patients in which antibiotic treatment can be safely withheld and those requiring expedited treatment and/or referral. There is currently limited use of available point-of-care tests for clinical biomarkers, including lactate, C-reactive protein, and procalcitonin, that can help make this distinction and determine the appropriate patient disposition. Elevated lactate has been shown to be associated with mortality among children hospitalized for pneumonia in Uganda (16). In addition, recent studies in Tanzania have demonstrated the potential of leveraging clinical decision algorithms (CDAs) that include point-of-care testing for CRP to reduce antibiotic use without increasing adverse outcomes among febrile children (17,18). However, these and other CDAs based on IMCI guidelines, including Integrated Community Case Management (iCCM), have not yet incorporated testing for specific respiratory pathogens (17–19). In addition, determining need for referral to a higher level facility is based on clinical signs alone or if any testing is included, it is only consists of an mRDT and hemoglobin level (17). To evaluate the potential utility of adding pathogen-specific assays and more extensive clinical biomarker testing to existing algorithms, more information is needed about the epidemiology of ARI and associated biomarker levels.

In our recent prospective study of patients presenting with fever to Kasese Health Center III (KHC) in western Uganda, only 18-32% were mRDT-positive (depending on the season), and 62% of mRDT-negative children presenting with fever reported cough (20). This suggests that non-malarial ARI represents a substantial proportion of visits for fever. In addition, as an exploratory aim, a subset of 73 patients in this study underwent procalcitonin testing, and only 16.4% were positive (>1 ng/mL) suggesting a bacterial etiology. However, all 73 of these patients received antibiotic treatment. Therefore, there is almost certainly a significant proportion of children in with febrile ARI in this region who have an undiagnosed viral infection in whom antibiotics could be avoided.

The overarching scientific goal of this study is to demonstrate the feasibility and preliminary effectiveness of rapid, point-of-care diagnostic tests that can be employed (1) at a rural health center for defining the epidemiology and guiding antibiotic treatment and (2) by village health workers (VHWs) for determining disposition of febrile respiratory illness in pediatric patients. Our hypothesis is that these commercially-available rapid tests can be used to discriminate bacterial and viral etiologies of febrile ARI, safely reduce unnecessary antibiotic prescriptions, and inform disposition decisions.

3.0 STUDY DESIGN

The work described herein seeks to evaluate rapid diagnostic tests (RDTs) to inform management of febrile ARI in two distinct contexts where many ill children are first evaluated – a Level III health center and in the patient's home by a VHW. To do this, the project will consist of two Phases, each consisting of a separate prospective, observational cohort study.

First, we plan conduct a prospective study of pediatric patients presenting to Kasese Health Center III with fever and respiratory symptoms (**Phase 1**). For those that test negative for malaria by mRDT, we will perform rapid testing for common causes of ARI as well as clinical biomarkers that effectively differentiate between viral and bacterial etiology of illness. As this study is observational in nature, no changes to clinical management will be made based on the results of rapid testing done for ARI. Patients will be followed up seven days after initial evaluation to determine outcomes related to their treatment and clinical course. During this phase, we plan to collect additional clinical samples for storage and future advanced molecular testing for etiologic pathogens and host responses from the study participants and a small group of healthy controls. Analysis of samples from healthy participants is necessary to differentiate colonization from true infection in the nasal mucosa and to serve as controls for the host gene expression data. Together, this information will be used to design algorithms for management of malaria-negative ARI that incorporate rapid testing and are feasible in settings with limited laboratory capacity.

In order to also assess the feasibility of equipping VHWs with rapid tests to improve the management of pediatric patients, we plan to conduct a separate prospective cohort study (**Phase 2**). For those children seen by VHWs in Bugoye and Maliba sub-counties of the Kasese District with fever and respiratory symptoms, we will perform a point-of-care lactate measurement. If markedly elevated (>5mmol/L), referral will be recommended given the risk of mortality associated with this degree of lactic acidosis. We will also record if the routine iCCM protocol would have independently recommended referral. Similar to Phase 1, children will be re-assessed at seven days after initial evaluation to determine outcomes related to their clinical course, disposition, and treatment.

Finally, in **Phase 3** of the study, we will focus in particular on the C-reactive protein (CRP) test. We will first compare three commercially-available rapid tests for CRP and one combination malaria/CRP test to a gold-standard lab-based CRP assay among children with fever presenting to Bugoye Health Center (**Part 1**). We will evaluate the tests' accuracy, cost, and ease-of-use. The best-performing test will then be added to routine iCCM protocols for VHWs in Bugoye sub-county to inform antibiotic treatment decisions, similarly to Phase 2, to create a study algorithm for evaluation of children with fever and respiratory symptoms. We will then conduct a stepped-wedge, cluster randomized trial to evaluate the safety of using the study algorithm and its impact on the frequency of antibiotic prescriptions as compared to the routine iCCM protocols (**Part 2**). Children will be re-assessed at seven days after initial evaluation as in Phases 1 and 2. As a sub-study of Part 2, we will conduct surveys and semi-structured interviews with participating VHWs to assess perceptions and feasibility of incorporating point-of-care diagnostics into clinical protocols.

4.0 SELECTION AND ENROLLMENT OF PARTICIPANTS

4.1 Inclusion Criteria

- Phase 1: Age 1-10 years, presentation to Kasese Health Center Outpatient Department (OPD) with fever and respiratory symptoms (documented (temperature > 38°C) or subjective fever in the last seven days, and fast breathing (respiratory rate > 30), cough, and/or hypoxia (oxygen saturation < 90%)).
  - We will also enroll a small group of healthy controls, specifically children age 1-10 years who are not ill (i.e. do not have fever or respiratory symptoms) and are presenting to the KHC OPD either for immunizations or minor traumatic injury.

- Phase 2: Age ≤ 5 years, evaluated by VHT in Bugoye or Maliba for febrile ARI (documented (temperature > 38°C) or subjective fever in the last seven days, and fast breathing (respiratory rate > 30) or cough)

- Phase 3:

  - Part 1: Age 6 months-5 years, presentation to Bugoye Health Center Outpatient Department with fever
  - Part 2: Age 2 months-5 years, evaluated by VHT in Bugoye for febrile ARI (documented (temperature > 38°C) or subjective fever in the last seven days, and fast breathing (respiratory rate > 30) or cough)
    - VHT sub-study: Participation as a VHW in the stepped wedge trial in Part 2

4.2 Exclusion Criteria

- Phase 1: Age <1 or ≥10 years at time of presentation, mRDT-positive

- Phase 2: Age >5 years at time of presentation

- Phase 3: Age >5 years at time of presentation

- For all phases, lack of guardian present to provide consent will also serve as an exclusion criterium.

4.3 Enrollment Procedures

Prior to the start of each phase, study staff will meet with local health partners (clinical staff and administration at KHC for Phase 1, the VHTs for Phase 2 and 3, and clinical staff and administration for Phase 3, Part 1) and community leaders about the aims and methods of the study. There will be an opportunity to ask questions, and together an appropriate timeline for each phase of the study will be determined. Prior to enrollment, all study and laboratory staff will undergo training conducted by experienced MUST and UNC personnel on the required technical skills, ethical conduct of research, and documentation standards. Topics of instruction will include (1) the consent process, (2) the use of the point-of-care tests, (3) follow-up procedures, and (4) record

keeping. The principal and co-investigator will be present during enrollment of the first study participants to audit the study procedure process.

Phase 1

On the specified date, study staff will meet with clinicians to begin study enrollment. All children 1-10 years of age presenting to KHC with febrile ARI as defined above will be screened (**Figure 1**). Patients will first undergo mRDT testing by finger or heel stick as per routine evaluation of children presenting with fever. If positive, the child will not be enrolled in the study and will be treated by clinicians according to local standard of care. If negative, the study coordinator will provide information on study objectives, protocol, and risks and benefits.

Potential healthy control participants will be identified by clinicians and study staff at time of presentation to the OPD or routine immunization clinic. They will be screened using a brief questionnaire asking about any current fever or respiratory symptoms that is included as part of the Initial Assessment Form (**Annex 1**). If found to be asymptomatic, they will be considered eligible for the study and the study coordinator will provide information on study objectives, protocol, and risks and benefits.

For all participants, informed consent will be sought from a parent or guardian. Children aged 8-10 years will also be asked to provide assent to study participation. Those individuals opting not to participate will undergo evaluation and treatment as per clinic protocols.

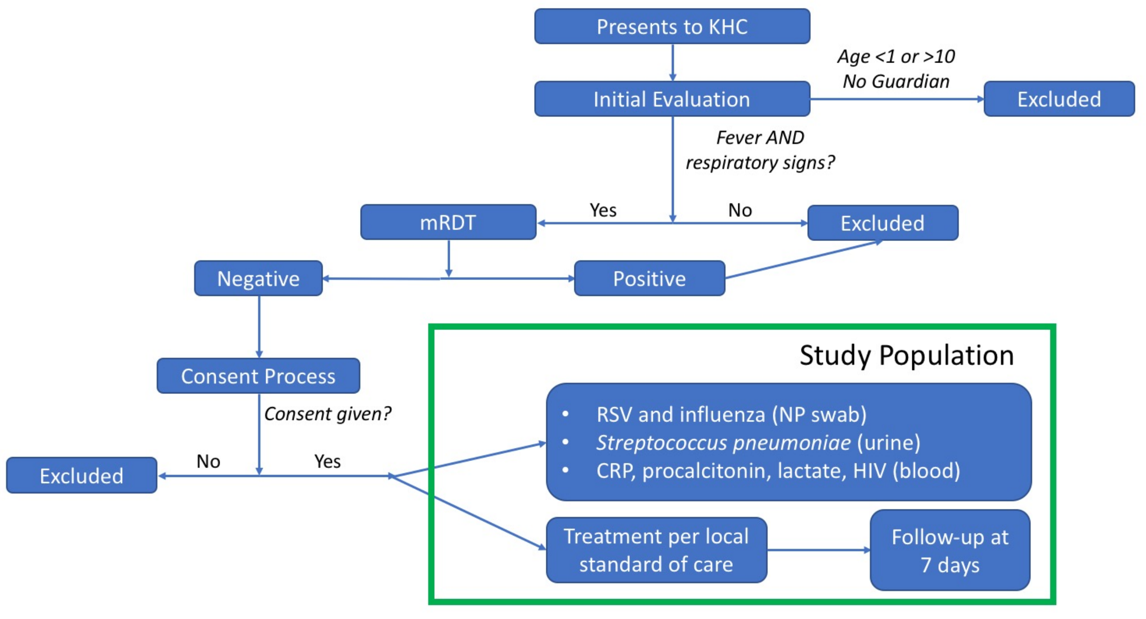

Figure 1. Anticipated Study Flow for Phase 1

Phase 2

Prior to the start of enrollment, two VHWs from each of five villages will undergo a one-week practical training at the local health facility to learn how to use the lactate meter and interpret the results. On the specified study start date, the VHWs will begin screening all children who they evaluate with febrile ARI. The VHWs will provide information on study objectives, protocol, and risks and benefits. Informed consent will be sought from a parent or guardian. Those individuals opting not to participate will undergo evaluation and treatment as per routine iCCM protocols.

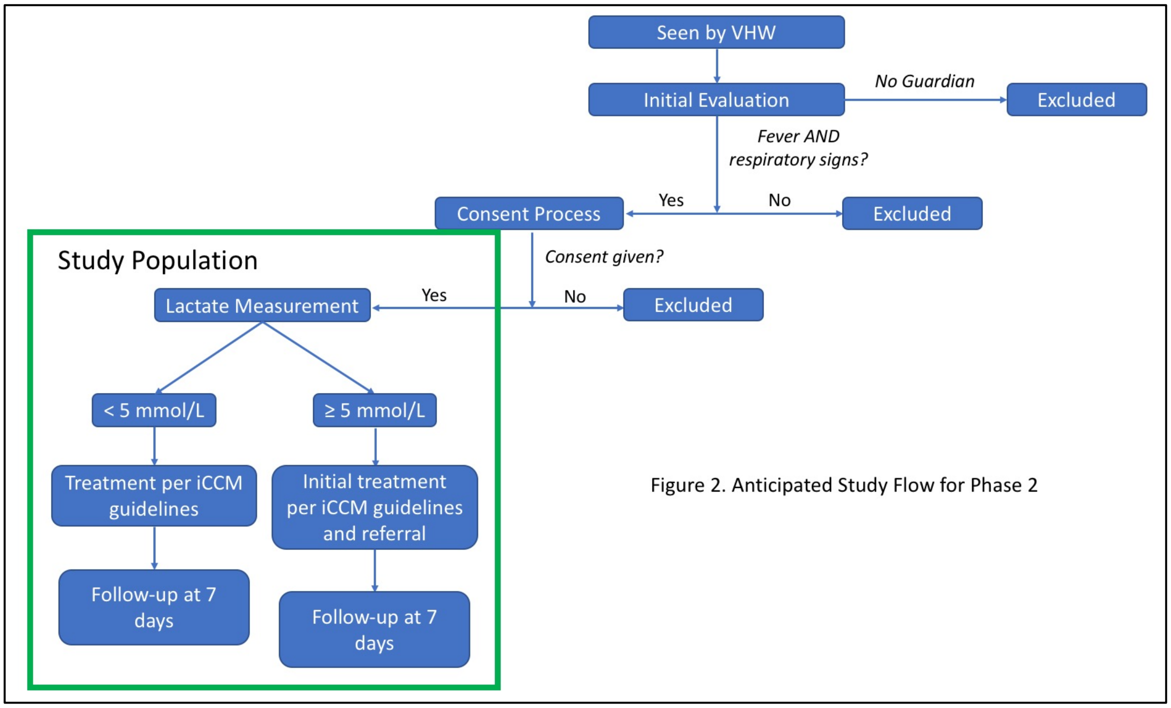

Phase 3

All children aged 6 months to 5 years presenting to Bugoye Health Center with fever be eligible for participation in **Part 1 of Phase 3.** Clinicians at BHC will identify eligible children refer them to the study coordinator. The coordinator will then provide information on study objectives, protocol, and risks and benefits to the potential subjects, and informed consent will be sought from a guardian. Those individuals opting not to participate will undergo evaluation and treatment per clinic guidelines.

For **Part 2 of Phase 3**, 5 VHTs from each of 15 villages in Bugoye sub-county will be selected by the study team for participation as care providers in the trial. They will then approach all children evaluated by study VHW in the Bugoye sub-county during the study period who meet the inclusion criteria - documented (temperature > 38°C) or subjective fever in the last seven days AND respiratory signs (fast breathing defined as RR > 30, cough, and/or hypoxia defined as oxygen saturation < 90%) regarding participation in the study. Subjects will be excluded if no guardian is available to provide consent. The VHW will then provide information on study objectives, protocol, and risks and benefits to the potential subjects, and informed consent will be sought from a guardian. Those individuals opting not to participate will undergo evaluation and treatment per iCCM guidelines regardless of whether the village is in a control or intervention period.

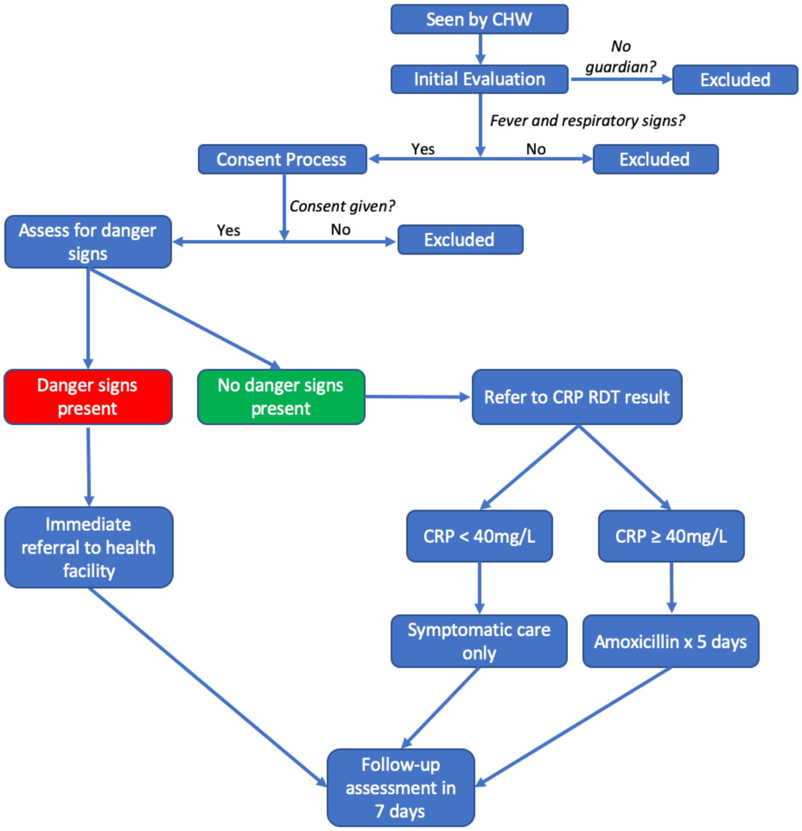

Figure 3. Anticipated Study Flow for Villages in Interventions Periods

5.0 STUDY TREATMENT OR INTERVENTION

In **Phase 1**, the study staff will perform the following activities for enrolled participants:

- Complete an encounter form documenting demographic and clinical information of each participant (**Annex 1**).

- Collect a venous blood sample (~3 mL), urine sample, and three nasopharyngeal swabs from enrolled children.

- Laboratory staff will perform the following rapid testing:
    - Blood sample: HIV, lactate, CRP, PCT
    - Urine sample: *Streptococcus pneumoniae*
    - NP swabs: RSV, influenza

- The remaining blood sample and one of the nasopharyngeal swabs will be stored for future testing, specifically next generation sequencing to identify pathogens not detected by RDTs and analysis of host gene expression in response to respiratory illness.

- Only the mRDT and HIV results will be available to the treating providers. They will administer treatments to the participants per local standards of care.

- The study coordinator will assess all participants (except the healthy controls) seven days after initial evaluation and complete a follow-up form (**Annex 2**).

In **Phase 2**, the study VHTs will perform the following activities:

- Complete an encounter form documenting demographic and clinical information of each participant (**Annex 3**).

- Evaluate participating children according to standard iCCM protocols.

- Measure a lactate level using ~50 microliters of blood obtained by finger or heel stick and document in the monthly register
    - If lactate >5mmol/L, provide initial management per iCCM and refer to nearest health facility or hospital
        - Note on encounter form if iCCM would have independently recommended referral
    - If lactate ≤ 5mmol/L, manage according to iCCM protocols

- Conduct a follow-up visit with participants seven days after initial assessment and complete a follow-up form (**Annex 2**)

In **Phase 3, Part 1**, the study staff will perform the following activities for enrolled patients:

- Complete an encounter form documenting demographic and clinical information of each participant (**Annex 5)**.

- Collect blood by finger or heel stick to perform the following study CRP tests (Actim CRP, Medix Biochemica, Finland, and Standard Q Malaria/CRP Duo, SD Biosensor, Korea (for the first 50 study participants only)) as well as the reference standard CRP test (Afinion CRP, Abbott Diagnostics, USA).

- The clinical providers will not have access to the results of any of the tests and will provide care per local guidelines

In **Phase 3, Part 2,** we will conduct a stepped wedge, cluster randomized trial as follows:

- Fifteen villages will be grouped into three strata based on village size, altitude, and proximity to Bugoye Health Centre.
    - Stratum A - low altitude, proximal, “large” villages (> 115 eligible children seen per year)
        - Bugoye, Ihani, Kanyaminigo, Muramba I, Ndugutu West
    - Stratum B - low altitude, mid-distance, “medium size” villages (90 to 140 eligible children seen per year)
        - Rwakingi 1B, Nyakabugha, Kibirizi, Katooke II, Kirongo
    - Stratum C - high altitude, distal, “small” villages (< 120 eligible children seen per year)
        - Five villages chosen from Ruboni, Mihunga, Kisamba II, Nyangonge, Bunyangoni, Mirimbo

- For the first month of the study, VHTs in ALL villages will:
    - Enroll children with fever and respiratory illness
    - Complete an encounter form documenting demographic and clinical information for each participant (**Annex 6**).
    - Evaluate and treat them according to standard iCCM protocols, including performing an mRDT.

- Each month for the next 5 months, the study team will randomly select a cluster of 3 villages, one village from each strata for transition to the intervention period of the study. Those that are not selected each month will continue study protocols as described above (Figure 4).
    - For the last month of the study, all 15 villages will be in the intervention part of the study.

Figure 4. Stepped Wedge Design

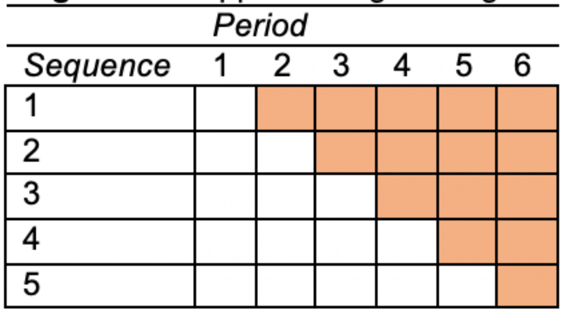

- During the intervention periods, VHTs will:
    - Enroll children with fever and respiratory illness
    - Complete an encounter form documenting demographic and clinical information for each participant (**Annex 6**).
    - Measure a CRP level using ~50 microliters of blood obtained by finger or heel stick and the best-performing CRP RDT from Part 1. Also, perform an mRDT.
    - Treat and manage enrolled children using the study algorithm adapted from standard iCCM protocols that uses the rapid CRP test result to inform antibiotic treatment decisions for those without danger signs (Figure 3).
        - If CRP > 40mg/L, prescribe amoxicillin

    - If CRP ≤ 40mg/L, manage according to iCCM guidelines, but do not prescribe amoxicillin

- For all control and intervention study periods, VHTs will assess for danger signs (severe chest in-drawing, inability to breastfeed or drink, and/or decreased level of consciousness). If present, they will refer patient to the nearest health facility and administer pre-referral management, including antibiotics, per iCCM protocols regardless of CRP result.

- Conduct a follow-up visit with participants seven days after initial assessment and complete a follow-up form (**Annex 7**)

For the **sub-study of Part 2**, we will conduct a qualitative analysis of the intervention period study algorithm as follows:

- All VHTs who participated as care providers in Part 2 will be asked to participate in surveys before the study and semi-structured interviews after the study.

- Surveys and semi-structured interviews will be conducted in the local language, Lukhonjo, by experienced, trained interviewers.

- Topics to be covered include perception of causes of ARI and antibiotic use for its treatment in children, feasibility of addition of CRP testing to clinical evaluation protocols, and perceived impact of testing results on clinical management decisions (Annexes 8, 9, and 10).

- The interviews will be audio-recorded, transcribed, and coded by a study team member for subsequent analysis.

6.0 CLINICAL AND LABORATORY INVESTIGATIONS

6.1 Clinical Investigations

Axillary temperature and pulse oximetry will be measured prior to clinical sample collection. All other clinical investigations will be per standard of care and not dictated by the study.

6.2 Laboratory Investigations

In **Phase 1,** participating children will have ~3mL of blood drawn by a laboratory technician to perform PCT (PCT-Plus, Boditech), CRP (NycoCard CRP, Abbott Diagnostics), and lactate (Lactate Plus, Sports Resource Group, Inc.) measurement as well as rapid HIV testing. Using the urine sample, laboratory technicians will conduct rapid testing for *Streptoccocus pneumoniae* antigen (BinaxNOW, Abbott Diagnostics). Finally, the collected NP swabs will be used for rapid influenza (SD Bioline, Abbott Diagnostics) and RSV (BinaxNOW, Abbott Diagnostics) antigen testing. The remaining blood will be stored in a DNA/RNA Shield Blood Tube (Zymo Diagnostics) and on dried blood spots to preserve nucleic acids for future testing as per Aim 1B.

As part of **Phase 2**, participating children will have ~50 µl of blood drawn by finger or heel stick for point-of-care lactate measurement (Lactate Plus, Sports Resource Group, Inc). The same blood sample will be used for mRDT testing if indicated per iCCM protocols.

In **Part 1 of Phase 3**, participating children will have ~100µL of blood collected from finger or heel stick in order to perform the mRDT and CRP tests (2 RDTs (Actim CRP, Medix Biochemica, Finland; CRP, BTNX, Canada)) and 1 lab-based assay (Afinion CRP, Abbot Diagnostics). Fifty of the study participants will also under to Malaria/CRP combination testing (Standard Q Malaria/CRP Duo, SD Biosensor, South Korea). It may take one to three finger or heel sticks to obtain the necessary blood sample. mRDT testing need to assess eligibility is part of routine standard of care.

In **Part 2 of Phase 3**, participating children will have ~50µL of blood collected from finger or heel stick to perform the mRDT and, if enrolled during an intervention period, the best-performing CRP RDT (as determined in Part 1).

6.3 Clinical Management

Phase 1: As this is an observational study, the clinical management of participants will be primarily dictated by the clinical providers and local standards of care. The only exceptions to that are as follows:

- If an mRDT performed as part of the study protocol is positive, treatment will be administered per local guidelines, and the child will be excluded from the remainder of the study as described above.
- If an HIV test performed as part of the study is positive, the child will be referred to the HIV clinic for further evaluation and treatment.

Phase 2: Treatment and referral plans of participating children will be primarily guided by iCCM protocols (**Annex 4**). Although lactate levels have not been prospectively evaluated in community-

based fever management programs, we will instruct VHWs to refer all children with a lactate ≥5mmol/L, even if not indicated by iCCM protocol, given the high mortality associated with such levels of lactic acidosis in previous studies. However, for the purpose of the analysis, children with lactate levels ≥5mmol/L who did not meet criteria for referral according to iCCM protocols will be classified as discharged from VHW care.

Phase 3:

- Part 1: All CRP testing done as part of this sub-study will be for research purposes only. The clinical management of the participants will be entirely determined by the clinical providers and local standards of care.
- Part 2: During the control periods, treatment and referral plans for participating children will be primarily guided by iCCM protocols (Annex 4), in particular as it relates to assessment and management of children with danger signs or symptoms. During the intervention periods, an mRDT and CRP test will be performed on all enrolled children. The VHW will evaluate all enrolled children for danger signs per iCCM protocols. If present, they will provide pre-referral treatment per iCCM protocols and immediately refer to a health centre. If no danger signs are present, the decision to administer antibiotic treatment will then be dictated by the CRP result. All children who test positive for malaria will be treated with antimalarial medication.

7.0 RISK ASSESSMENT & MITIGATION

7.1 Risks associated with study participation

There may be some minimal discomfort associated with the finger or heel stick, blood draw, and/or nasopharyngeal swab collection. In addition, with the blood draw, there is a small risk of bruising and fainting, and a very rare risk of infection.

There is also the risk of loss of privacy and/or confidentiality concerning medical history should any study documents be compromised or lost.

7.2 Risk Mitigation

To minimize adverse events related to blood draws and finger or heel sticks, all participants shall have samples drawn only by trained study or laboratory staff. Participants shall be informed of all procedures to be done ahead of time. They will be reminded that they have the right to decline any procedure at any time. If an adverse event occurs, affected participants will be immediately assisted to a level III health facility for expedited evaluation.

Universal precautions, including the provision and use of personal protective equipment (PPE), will be required of all study staff when collecting blood. All sharp equipment will be disposed of in appropriate containers. Any staff member who is exposed (example: needle stick) will undergo diagnostic testing (hepatitis, HIV) and receive prophylactic treatment, provided by the study.

To minimize the risk of loss of privacy, patients will be moved to a private area of the clinic or home during informed consent discussions. To minimize the risk of loss of confidentiality. each patient will be assigned a unique identification number on enrolment. Identifying information, specifically name, address, and phone number, will be collected to facilitate follow-up assessments, but once these are complete, the identification number will be the only link between patient information and the study testing results. Once entered into REDCap, the data will be encrypted during transmission and stored on a secure server. Only study staff will have access to the records. All information stored on paper will be scanned and destroyed upon completion of the study.

Household locations may be visually represented in publication, but these will not be linked to individual data and the scale will not be sufficient to locate individual households. Any paper documents, including consent forms, will be scanned and destroyed upon completion of the study.

All laboratory specimens, evaluation forms, reports, and other records that leave the site will be identified by coded number only to maintain participant confidentiality. All records will be kept locked. All computer entry and networking programs will be done with coded numbers only.

8.0 ADVERSE EVENT REPORTING

8.1 Definitions

An adverse event (AE) is any untoward medical occurrence in a patient or clinical investigation participant administered a pharmaceutical product and which does not necessarily have a causal relationship with this treatment. An adverse event can therefore be any unfavorable and unintended sign (including an abnormal laboratory finding), symptom, or disease temporally associated with the use of a medicinal (investigational) product, whether or not related to the medicinal (investigational) product.

An unexpected (unlisted) adverse event is an adverse reaction, the nature or severity of which is not consistent with the applicable product information.

A serious adverse event (SAE) is any untoward medical occurrence that at any dose:

- results in death;
- is life-threatening;
- requires inpatient hospitalization or prolongation of existing hospitalization;
- results in a persistent or significant disability/incapacity;

Important medical events as assessed by medical and scientific judgment may also be considered SAEs by the investigator and will be reported in an expedited fashion.

8.2 Reporting

Given the lack of any medical intervention or investigational pharmaceutical treatment, we do not anticipate any adverse events. In the case of any adverse events related to phlebotomy or NP swab collection, the survey team will immediately coordinate motorized transportation to the nearest level III health facility for expedited evaluation by a clinical officer. If further evaluation is needed, the CO will notify the In-Charge, who will maintain a small transportation fund, to facilitate transportation and onward referral to St. Paul's Level IV Health Center in Kasese or Kilembe Mines Hospital in Kilemebe.

The study coordinator will maintain a log of possible study-related adverse events and will report any unexpected or serious adverse events to the PI, who will communicate the event to the MUST IRC and UNC IRB within 24 hours.

9.0 STATISTICAL CONSIDERATIONS

9.1 General Design Issues:

The proposed work consists of three prospective studies to gain information regarding the feasibility and preliminary effectiveness of using rapid tests to improve diagnosis and management of febrile ARI among pediatric patients. The first two phases of the study, as well as part 1 of the third phase, which will lay the foundations for future interventional studies, are observational. Part 2 of Phase 3 is a stepped-wedge cluster randomized trial where the intervention is an algorithm for evaluation of ARI by VHW that incorporates CRP point-of-care testing.

9.2 Endpoints

Study participation will begin at the time of consent and conclude at the time of the follow-up assessment seven days after the initial assessment. Our outcomes of interest for both Phases 1 and 2 and Part 2 of Phase 3 include the following: frequency of antibiotic prescription at initial assessment, duration of symptoms, antibiotic failure defined as need for initiation or change in antibiotic therapy after the initial assessment, repeat evaluation at a health facility, acquisition/administration of antibiotics from another source (e.g. drug shop), hospitalization, and death.

9.3 Randomization and Stratification:

As this study is observational in nature, there will be no randomization or stratification. All patients meeting inclusion criteria will be approached regarding participation in the study until our target sample size is reached.

9.4 Sample Size and Accrual

Phase 1: Given the exploratory nature of this study, we did not conduct a formal sample size calculation. There are approximately 700 visits to KHC per month by children under 5 years of age. In our recent study, approximately 20-30% of children presenting with fever were mRDT-positive. Of those that were mRDT-negative, 62% reported cough. Assuming a participation rate of 90%, we estimate that it will take 1-2 months to enroll our target 250 participants.

Phase 2: Based on our previous experience at the site, we estimate that each VHW will assess an average of 11 patients each month. Therefore, we have elected to train 10 VHWs to use the lactate plus device, which will result in a total of approximately 330 participants over the three-month period of observation. We will assume a 10% loss to follow up rate, which should leave 300 patients for the primary analysis.

Phase 3: For **Part 1**, assuming a non-inferiority threshold of 5%, a sample size of 150 will achieve > 90% power to demonstrate non-inferiority between each of the 3 CRP only RDTs and the reference standard in regard to identifying those with a CRP ≥ 40mg/L. Our primary outcome of interest is the proportion of children evaluated by VHW for febrile ARI who are prescribed antibiotics (**Part 2**). In previous studies, approximately 80-95% of children evaluated by CHW for fast breathing received antibiotics. We anticipate that this proportion will be similar, if not

higher, among children who also have fever, although patients with fever and cough in the absence of fast breathing may be prescribed antibiotics less frequently. Therefore, we conservatively estimate that 80% of patients evaluated by CHW for fever and any respiratory sign currently receive antibiotics. The GEE power calculation method of Rochon (1998) for population-averaged models is applied to the binary outcome antibiotic use for the stepped wedge design in Figure 4. Assuming ten children recruited per village-month, the power to detect a difference in proportions of 0.20 with two-sided α=0.05 GEE Wald tests will be greater than 80% for a wide range of plausible intraclass correlations (**Table 1**). Li et al. have shown that the analytical power derived by this method agrees well with simulated power for as few as eight clusters when data are analyzed using the bias-adjusted GEE procedure reference above (21). Therefore, our goal prior to study start was to enroll 10 children per month per village, or a total of 900 children. Through the first 4 months of the study, enrollment has been more successful than anticipated, with ~15 children enrolled per village per month. Therefore, we will increase our target sample size to 1,375 children to ensure approximately the same number of children enrolled in each study period. The power to detect a difference in proportions of 0.20 with two-sided α=0.05 GEE Wald tests will then be greater than 90% for a wide range of plausible intraclass correlations.

Table 1. Statistical Power for two-sided, α=0.05 significance tests in the marginal model analysis of intervention effect in a stepped wedge cross-sectional design assuming three villages per treatment sequence.

		Number of children per village/month		
α_0	α_1	8	**10**	12
0.025	0.0125	93	**96**	98
0.05	0.025	88	**93**	96
0.10	0.05	80	**86**	89
0.15	0.075	73	**79**	82

[1]The baseline (control) rate of antibiotic prescribing is assumed to be 0.80 and the rate under the intervention condition is 0.60, corresponding to an odds ratio (e^{δ}) of 0.375; we assume a logistic model with $\beta_j = \log(0.8/0.2)$.

9.5 Monitoring

Because this study is observational in nature and does not include any therapeutic or pharmacologic intervention, no Data and Safety Monitoring Board (DSMB) is planned.

9.6 Analyses

Phase 1

We will use the results of the study at KHC for the following analyses:

- Estimate the proportion of cases of malaria-negative, febrile respiratory illness for which an etiology can be determined using commercially available point-of-care rapid diagnostic tests.

- Determine the proportion of children presenting with malaria-negative, febrile ARI who test positive for RSV and influenza.

- Determine the proportion of children presenting with malaria-negative, febrile ARI who pneumococcal pneumonia (based on positive *Streptococcus pneumoniae* urine antigen testing AND an elevated CRP).

- Using next-generation sequencing-based shotgun metagenomics to evaluate the nasopharyngeal sample and blood for causes of febrile ARI that are not detectable by rapid testing.

- Assess for differences in host gene expression (1) between participants diagnosed with viral versus bacterial etiology of febrile ARI, (2) between healthy controls and participants diagnosed with viral infection, and (3) between healthy controls and participants diagnosed with bacterial infection.

- Evaluate for any associations between rapid test and clinical biomarker results and our outcomes of interest: duration of symptoms, antibiotic failure defined as need for initiation or change in antibiotic therapy after the initial assessment, repeat evaluation at a health facility, acquisition/administration of antibiotics from another source (e.g. drug shop), hospitalization, and death.

- Measure the impact of rapid testing on clinic throughput by comparing the number of patients seen and visit duration before and during the study

Phase 2:

We will use the results of the study involving VHWs to perform the following analyses:

- Quantify the prevalence of lactic acidosis among children presenting to VHWs with febrile ARI in the Bugoye and Maliba sub-counties.

- Determine the sensitivity, specificity, negative predictive value, and positive predictive value of iCCM protocols for identifying children with an elevated lactate.

- Evaluate for associations between lactic acidosis and our outcomes of interest: duration of symptoms, antibiotic failure defined as need for initiation or change in antibiotic therapy after the initial assessment, repeat evaluation at a health facility, acquisition/administration of antibiotics from another source (e.g. drug shop), hospitalization, and death.

Phase 3: We will use the results of the CRP study for the following analyses

- Part 1
  - Determine the accuracy of each CRP RDT by calculating the sensitivity and specificity as compared to the gold standard, lab-based CRP assay
  - Assess feasibility of RDT use by calculating the median time to perform each test
  - Select the best-performing RDT by determining which one maximizes accuracy, feasibility and cost.

- Part 2
  - Calculate the prevalence of elevated CRP (>40mg/L) among children presenting to VHTs with fever and respiratory symptoms

- Assess for differences in frequency of antibiotic prescription at initial VHW evaluation and symptom resolution at follow-up visit between CRP-guided and routine iCCM protocols

- Evaluate for any associations between CRP RDT results and our outcomes of interest: duration of symptoms, antibiotic failure defined as need for initiation or change in antibiotic therapy after the initial assessment, repeat evaluation at a health facility, acquisition/administration of antibiotics from another source (e.g. drug shop), hospitalization, and death.

- The audio recordings of the semi-structured interviews will be transcribed verbatim. Qualitative data will be analyzed following an inductive, grounded theory approach. The codes will be refined and sharpened using the data collected, with any disagreements resolved through consensus of the study team, to produce a final set of codes that will be used to re-code all the transcripts.

10.0 STUDY MANAGEMENT

10.1 Institutional Review Board (IRB) Review and Informed Consent

This protocol and the informed consent documents and any subsequent modifications (amendments) will be reviewed and approved by the Mbarara University of Science and Technology REC, the Uganda National Council of Science and Technology (UNCST), and the UNC IRB. It is expected that the IRB will have the proper representation and function in accordance with federally mandated regulations.

In obtaining and documenting informed consent, the investigator will comply with the applicable regulatory requirement(s), and should adhere to Good Clinical Practice (GCP) and to ethical principles that have their origin in the Declaration of Helsinki.

Before recruitment and enrollment onto this study, the participant will be given a full explanation of the study and will be given the opportunity to review the consent form(s). Each consent form will include all the relevant elements currently required by the local regulations, which include elements such as the purpose of the study, the procedures to be followed, and the risks and benefits of participation. Once this essential information has been provided to the participant and the investigator is assured that the participant understands the implications of participating in the study, the participant will be asked to give consent to participate in the study by signing a REC/IRB-approved consent form. During Part 2 of Phase 3, both the VHW implementing the study and the children they enroll will complete separate consent forms as both are considered research subjects.

Prior to a participant's participation in the trial, the written informed consent form(s) will be signed and personally dated by the participant or the participant's legally authorized representative (parent or guardian), and by the person who conducted the informed consent discussion.

10.2 Registration Procedures

All participants will be identified by a unique identifier and not by name. Identifying information including name, phone number, and household location will be recorded to facilitate follow-up assessments at seven days after enrollment. Once this evaluation is complete, the identification number will be the only link between patient information and the study testing results.

10.3 Participant Incentives

In Phase 1, participants, including healthy controls, will receive the following for participating in this study:

- 5,000 shillings to cover the cost of transportation to the clinic
- A bar of soap for washing
- 1kg of sugar
- A cookie and a soda for the child.

There are no incentives for participation in Phase 2; there are no additional procedures anticipated as children with fever undergo mRDT testing by finger or heel stick per iCCM protocols.

In Phase 3, the children/caregiver will receive the following for participating in the study:

- A bar of soap for washing
- A cookie and a soda for the child.

In Phase 3, Part 2, the participating VHTs will receive 20,000UGX per month for their participation and to reimburse them for the cost of transportation to bring their data collection forms to the VHW office in Bugoye (this is the current system for VHW program documents). Those who complete the end of study semi-structured interview will also receive a snack and a drink during the semi-structured interview.

10.4 Criteria for Discontinuation

If, at any time the constraints of this protocol are detrimental to the participant's health or believed not to be in the patient's best interests by study staff, even if not previously addressed, and/or the participant no longer wishes to continue study treatment, we will remove the participant from further consideration.

Furthermore, if an adequate specimen is not obtained after three finger/heel sticks or blood draw attempts, no further attempts will be made and the individual will be excluded.

10.5 Deviations from the Protocol

The investigator may implement a deviation from, or a change of, the protocol to eliminate an immediate hazard(s) to study participants without prior REC/IRB approval/favorable opinion. As soon as possible, the implemented deviation or change, the reasons for it, and, if appropriate, the proposed protocol amendment(s) should be submitted to (i) REC/IRB for review and approval, (ii) the sponsor for agreement and, if required and (iii) the regulatory authority(ies).

10.6 Adherence to the Protocol

Except for an emergency situation in which proper care for the protection, safety, and well-being of the study participant requires alternative treatment, the study shall be conducted exactly as described in the approved protocol. Any deviation from the protocol must have prior approval by the Principal Investigator and must be recorded and explained.

10.7 Amendments to the Protocol

Should amendments to the protocol be required, the amendments will be originated and documented by the Principal and Co-investigators. The written amendment will be sent to investigators and must be submitted to the IRB at the investigator's site for approval. It should also be noted that when an amendment to the protocol substantially alters the study design or the potential risk to the participant, a revised consent form might be required.

10.8 Record & Sample Retention

Study documentation will include all source documents and regulatory documents (e.g., protocol and amendments, IRB correspondence and approval, signed participant consent forms). Source documents will include all recordings of observations or notations of clinical activities and all reports and records necessary for the evaluation and reconstruction of the clinical research study.

The proposed study does include the collection and storage of blood samples in tubes and on filter paper and NP swabs in tubes for analysis at a later date. These samples will be identified only by the coded ID. Samples will be stored for the genomic analyses mentioned above as part of Aim 1B. All samples will be stored in Uganda at Epicentre Research Base in Mbarara until testing occurs. Any additional utilization of these samples, however, will require a modification to the current REC/IRB application prior to initiation.

10.9 Participant Confidentiality

All laboratory specimens, evaluation forms, reports, and other records that leave the site will be identified by coded number only to maintain participant confidentiality. All records will be kept locked. All computer entry and networking programs will be done with coded numbers only. Clinical information will not be released without written permission of the participant.

11.0 CAPACITY BUILDING

11.1 Students & Trainees

Pending interest, we will engage a Masters student from the Department of Community Health as a research assistant for this study. Responsibilities would include assisting with supervision of enrollment, data entry and analysis, and manuscript preparation. Other projects that involve further analysis of the obtained data could also be made available to other students and trainees.

11.2 Investigators & Study Staff

The proposed study will employ electronic tablets for data collection in RedCap (www.redcap.unc.edu). Field staff will be trained to use this application and will receive certificates at the completion of the study. In addition, laboratory staff will gain new skills as it relates to conducting a broader array of rapid diagnostic tests.

11.3 Local Officials & Infrastructure

The proposed study will provide important information to leaders and policymakers at the village, sub-county, and district level. Enhanced knowledge regarding the epidemiology of acute respiratory illness in the area could inform clinical practice and policies. In addition, understanding how well iCCM protocols identify the sickest children could help shape future improvements of those guidelines.

12.0 DISSEMINATION OF RESULTS

12.1 Data Sharing

Data ownership will be jointly shared between Drs. Mulogo and Boyce. Based on the background and history of collaboration between UNC and collaborators in Uganda, we do not anticipate problems in resolving conflicts over data access or use. However, we will develop procedures for disagreements if they arise. We will first attempt to resolve the conflict within the group. This includes discussions about any issue, alternative approaches to a solution. If unsuccessful, the PIs and key personnel will escalate the problem to an agreed upon arbiter, who will be asked to adjudicate within a two-week period (or sooner if urgent).

12.2 Dissemination

The results of the study will be shared with the community upon completion, through meetings with local health partners and the district leadership. A poster, in both English and Lukhonjo, outlining the study results will be placed at Kasese and Bugoye Health Centers. Representatives from the office of the respective District Health Offices (DHO), including the District Laboratory Officers, will be updated regularly on the progress of the study. The final results will be shared with the DHOs prior to any presentation and/or publication.

From the study, we anticipate at least three publications in an international scientific journal, such as the *American Journal of Tropical Medicine and Hygiene*. The results may also be presented at various conferences pending acceptance of any abstract or poster. All participating team members will be acknowledged, and those contributing to the analysis and/or manuscript preparation will be listed as a co-author.

13.0 REFERENCES

1. Le Doare K, Bielicki J, Heath PT, Sharland M. Systematic Review of Antibiotic Resistance Rates Among Gram-Negative Bacteria in Children With Sepsis in Resource-Limited Countries. Journal of the Pediatric Infectious Diseases Society. 2015 Mar;4(1):11–20.

2. World Health Organization. Global Action Plan on Antimicrobial Resistance [Internet]. Geneva, Switzerland: World Health Organization; 2015. Available from: https://apps.who.int/iris/bitstream/handle/10665/193736/9789241509763_eng.pdf

3. D'Acremont V, Lengeler C, Genton B. Reduction in the proportion of fevers associated with Plasmodium falciparum parasitaemia in Africa: a systematic review. Malaria Journal. 2010;9(1):240.

4. Maze MJ, Bassat Q, Feasey NA, Mandomando I, Musicha P, Crump JA. The epidemiology of febrile illness in sub-Saharan Africa: implications for diagnosis and management. Clinical Microbiology and Infection. 2018 Aug 1;24(8):808–14.

5. Guillebaud J, Bernardson B, Randriambolamanantsoa TH, Randrianasolo L, Randriamampionona JL, Marino CA, et al. Study on causes of fever in primary healthcare center uncovers pathogens of public health concern in Madagascar. Bailey RL, editor. PLOS Neglected Tropical Diseases. 2018 Jul 16;12(7):e0006642.

6. D'Acremont V, Kilowoko M, Kyungu E, Philipina S, Sangu W, Kahama-Maro J, et al. Beyond Malaria — Causes of Fever in Outpatient Tanzanian Children. New England Journal of Medicine. 2014 Feb 27;370(9):809–17.

7. Elfving K, Shakely D, Andersson M, Baltzell K, Ali AS, Bachelard M, et al. Acute Uncomplicated Febrile Illness in Children Aged 2-59 months in Zanzibar – Aetiologies, Antibiotic Treatment and Outcome. Lin B, editor. PLOS ONE. 2016 Jan 28;11(1):e0146054.

8. World Health Organization. Maternal, Newborn, Child and Adolescent Health: Causes of Death Among Children [Internet]. [cited 2018 Aug 2]. Available from: http://www.who.int/maternal_child_adolescent/data/causes-death-children/en/

9. Ocan M, Aono M, Bukirwa C, Luyinda E, Ochwo C, Nsambu E, et al. Medicine use practices in management of symptoms of acute upper respiratory tract infections in children (≤12 years) in Kampala city, Uganda. BMC Public Health [Internet]. 2017 Dec [cited 2018 Aug 2];17(1). Available from: http://bmcpublichealth.biomedcentral.com/articles/10.1186/s12889-017-4770-1

10. Risk R, Naismith H, Burnett A, Moore SE, Cham M, Unger S. Rational prescribing in paediatrics in a resource-limited setting. Archives of Disease in Childhood. 2013 Jul 1;98(7):503–9.

11. Ndhlovu M, Nkhama E, Miller JM, Hamer DH. Antibiotic prescribing practices for patients with fever in the transition from presumptive treatment of malaria to 'confirm and treat' in Zambia: a cross-sectional study. Tropical Medicine & International Health. 2015 Dec;20(12):1696–706.

12. Uganda Bureau of Statistics (UBOS) and ICF International. Uganda Malaria Indicator Survey 2014-2015. Kampala, Uganda and Rockville, Maryland: UBOS and ICF International; 2015.

13. World Health Organization, Department of Maternal N Child and Adolescent Health, World Health Organization. Revised WHO classification and treatment of pneumonia in children at health facilities: evidence summaries. [Internet]. 2014 [cited 2018 Oct 17]. Available from: http://apps.who.int/iris/bitstream/10665/137319/1/9789241507813_eng.pdf?ua=1

14. Mukanga D, Konaté AT, Cousens S, Amenga-Etego L, Pagnoni F, Tibenderana JK, et al. Integrated Community Case Management of Fever in Children under Five Using Rapid Diagnostic Tests and Respiratory Rate Counting: A Multi-Country Cluster Randomized Trial. The American Journal of Tropical Medicine and Hygiene. 2012 Nov 7;87(5_Suppl):21–9.

15. Means AR, Weaver MR, Burnett SM, Mbonye MK, Naikoba S, McClelland RS. Correlates of Inappropriate Prescribing of Antibiotics to Patients with Malaria in Uganda. Diemert DJ, editor. PLoS ONE. 2014 Feb 28;9(2):e90179.

16. Gunaratnam LC, Conroy AL, Ma C, Namasopo S, Hawkes MT, Ericson A, et al. Handheld Point-of-Care Lactate Measurement at Admission Predicts Mortality in Ugandan Children Hospitalized with Pneumonia: A Prospective Cohort Study. The American Journal of Tropical Medicine and Hygiene [Internet]. 2018 Nov 5 [cited 2019 Jan 4]; Available from: http://www.ajtmh.org/content/journals/10.4269/ajtmh.18-0344

17. Keitel K, Kagoro F, Samaka J, Masimba J, Said Z, Temba H, et al. A novel electronic algorithm using host biomarker point-of-care tests for the management of febrile illnesses in Tanzanian children (e-POCT): A randomized, controlled non-inferiority trial. Tumwine JK, editor. PLOS Medicine. 2017 Oct 23;14(10):e1002411.

18. Rambaud-Althaus C, Shao AF, Kahama-Maro J, Genton B, d'Acremont V. Managing the Sick Child in the Era of Declining Malaria Transmission: Development of ALMANACH, an Electronic Algorithm for Appropriate Use of Antimicrobials. Borrmann S, editor. PLOS ONE. 2015 Jul 10;10(7):e0127674.

19. Keitel K, D'Acremont V. Electronic clinical decision algorithms for the integrated primary care management of febrile children in low-resource settings: review of existing tools. Clinical Microbiology and Infection. 2018 Aug;24(8):845–55.

20. Boyce RM. Personal Communication: Demographic and clinical characteristics of children presenting to the Kasese Health Center in May 2018. 2018.

21. Li F, Turner EL, Preisser JS. Sample size determination for GEE analyses of stepped wedge cluster randomized trials. Biom. 2018 Dec;74(4):1450–8.

OFFICE OF HUMAN RESEARCH ETHICS
720 Martin Luther King, Jr. Blvd.
Bldg. 385, 2nd Floor
CB #7097
Chapel Hill, NC 27599-7097
(919) 966-3113
Web site: ohre.unc.edu
Federalwide Assurance (FWA) #4801

To: Emily Ciccone and Ross Boyce
Medicine-Infectious Diseases

From: Biomedical IRB

Approval Date: 10/09/2019
Expiration Date of Approval: 4/28/2020
RE: Notice of IRB Approval by Full Board Review
Submission Type: Modification
Study #: 18-2803

Study Title: Rapid Tests for Evaluation of Malaria-negative Children with Febrile Respiratory Illness in Rural Uganda

This submission, Reference ID 251183, has been approved by the IRB for the period indicated. Unless otherwise noted, regulatory and other findings made previously for this study continue to be applicable.

Submission Description:

We have recently received additional funding from the Thrasher Research Fund to conduct a sub-study that we are adding to this application as a fourth aim. It will be very similar to Aim 3, with the exception that the test being evaluated will be a point-of-care C-reactive protein (CRP) rapid diagnostic test. It will enroll separately from Aim 3, and therefore, the forms below have been added to the application.

Documents Added:
ARIStudy_Annex5_BHCForm_2Aug: Data collection form for Part 1 of Phase 3

ARIStudy_Annex6_CHWCRPForm_2Aug: Data collection form for initial assessment during Phase 3

ARIStudy_CRP_REDCapForms: Electronic version of data collection forms for Phase 3

ARISTUDY_Annex4 added in response to board stipulation

Added a device document describing the CRP tests that will be used in the study that includes the package inserts and an Investigational Device Worksheet.

Investigator's Responsibilities:

Your approved consent forms and other documents are available online at http://apps.research.unc.edu/irb/index.cfm?event=home.dashboard.irbStudyManagement&irb_id=18-2803.

The current data security level determination is Level III. Any changes in the data security level need to be discussed with the relevant IT official. If data security level II and III, consult with your IT official to develop a data security plan. Data security is ultimately the responsibility of the Principal Investigator.

This study was reviewed in accordance with federal regulations governing human subjects research, including those found at 45 CFR 46 (Common Rule), 45 CFR 164 (HIPAA), 21 CFR 50 & 56 (FDA), and

40 CFR 26 (EPA), where applicable.

CC:
Jonathan Juliano, Medicine-Infectious Diseases

MBARARA UNIVERSITY OF SCIENCE AND TECHNOLOGY

P.O. Box 1410, Mbarara Uganda. Tel: +256 485433795; Fax: +256 4854 20782

RESEARCH ETHICS COMMITTEE

E-mail: sec.rec@must.ac.ug

Our Ref: MUREC 1/7 **Date:** June 11, 2020

Assoc Prof Edgar Mulogo
Principal Investigator

Re: **Request for amendment of approved study protocol on "Rapid tests for the evaluation of children with malaria-negative, febrile respiratory illness in rural Uganda"**

Type: [ ] Initial Application
[x] Protocol Amendment
[ ] Letter of Amendment (LOA)
[] Continuing Review
[ ] Material Transfer Agreement
[ ] Other, specify: ______________________________

MUST REC has received and reviewed your request for amendment of an approved study. The following amendments have been approved: 1) to enroll a small group of healthy controls n=25, as part of Phase 1 of the study 2) evaluation of a point-of-care C-reactive protein rapid diagnostic test 3) train local village health worker in Bugoye sub-county to use and incorporate the CRP test into standard iCCM protocols to inform antibiotic prescription decision-making

The following is the list of documents approved in the application:

**Document**	**Language**	**Version date**
ARIStudy_Annex5_BHCForm_2Aug	English	**October 2019**
ARI Study_Annex6_CHWCRPForm_2Aug	English	**October 2019**
ARI Study_Informed consent BHC_CRP LHUKONZO FINAL	Lhukonzo	**October 2019**
ARI Study_Informed Consent Form_BHCCRP_ENG_2Aug	English	**October 2019**
ARI Study_Informed Consent Form_CHW_CRP LUKONZO FINAL	Lhukonzo	**October 2019**
ARI Study_Informed Consent Form_CHWCRP_ENG_2Aug	English	**October 2019**

I wish you all the best in continuation of your study.

Dr. Francis Bajunirwe
CHAIR,
MUST RESEARCH ETHICS COMMITTEE

Uganda National Council for Science and Technology
(Established by Act of Parliament of the Republic of Uganda)

UNCST

Our Ref: HS 2631 **25th March 2021**

Assoc. Prof. Edgar Mugema Mulogo
Principal Investigator
Mbarara University of Science and Technology
Mbarara

Dear Assoc. Prof. Mulogo,

RE: RAPID TESTS FOR THE EVALUATION OF CHILDREN WITH MALARIA – NEGATIVE, FEBRILE RESPIRATORY ILLNESS IN RURAL UGANDA

This is to inform you that on **25th March 2021**, Uganda National Council for Science and Technology (UNCST) reviewed the request for renewal of the above named study and granted approval.

UNCST has granted continuing approval valid until **23rd July 2024**. If, however, it is necessary to continue with the study beyond the expiry date, a request for continuation should be made to the Executive Secretary, UNCST.

Yours sincerely,

Isaac Makhuwa
for: Executive Secretary
UGANDA NATIONAL COUNCIL FOR SCIENCE AND TECHNOLOGY

LOCATION/CORRESPONDENCE

Plot 6 Kimera Road, Ntinda
P.O.Box 6884
KAMPALA, UGANDA

COMMUNICATION

TEL: (256) 414 705500
FAX: (256) 414-234579
EMAIL: info@uncst.go.ug
WEBSITE: http://www.uncst.go.ug

THE UNIVERSITY of NORTH CAROLINA at CHAPEL HILL

OFFICE OF HUMAN RESEARCH ETHICS
104 Airport Drive
Suite 2100
CB #7097
Chapel Hill, NC 27599-7097
(919) 966-3113
Web site: ohre.unc.edu
Federalwide Assurance (FWA) #4801

To: Emily Ciccone and Ross Boyce
Medicine-Infectious Diseases

From: Biomedical IRB

Approval Date: 4/09/2021
Expiration Date of Approval: 5/10/2021
RE: Notice of IRB Approval by Expedited Review (under 45 CFR 46.110)
Submission Type: Modification
Expedited Category: Minor Change to Previously Approved Research
Study #: 18-2803

Study Title: IGHID 12110 - Rapid Tests for Evaluation of Malaria-negative Children with Febrile Respiratory Illness in Rural Uganda

This submission, Reference ID 320886, has been approved by the IRB for the period indicated. It has been determined that the risk involved in this modification is no more than minimal. Unless otherwise noted, regulatory and other findings made previously for this study continue to be applicable.

Summary of changes approved with this submission:

- Personnel update: we are adding Grace Onyebuchi, Rachel Cook, Erin Daniel and Tania Hossain regulatory associates.
- Added IGHID number to the study title for tracking purposes.
- We are changing the study design of Phase 3, Part II from a pre-post design to a stepped wedge cluster randomized trial. The community health workers in 15 villages will be part of the trial. All will start out assessing children with fever and acute respiratory illness using the usual government protocols (control). Every month, three villages will "cross-over" into the intervention part of the study when they will instead use the study algorithm that includes a point-of-care C-reactive protein test to assess the children with fever and Acute respiratory illness. Our previous plan was to use a pre/post design. However, this design could introduce bias because the relative frequency of the causes of respiratory illness may vary between the pre and post periods. This variation may confound the relationship we are trying to observe between our intervention, the study algorithm, and our primary outcome, the rate of antibiotic prescription. Logistically, it is not feasible to measure the relative frequencies of all potential causes of ARI to control for seasonality. Therefore, we propose to transition to a cluster-randomized, stepped wedge study design. This efficient trial design maximizes resources (given our budgetary and logistical constraints on number of included villages and duration of follow-up), allows all participating CHWs to learn the CRP-guided algorithm, and minimizes treatment contamination. It also allows us to control for potential confounders that may be unanticipated and/or unmeasured.
- We are adding a small qualitative sub-study to Phase 3 to assess the feasibility and acceptability of adding the CRP test to existing CHW algorithms among the CHW care providers involved in the study.

We are adding an MPH student, Sinead Walsh, to our project. She will be a research assistant helping with the qualitative sub-study approved during our last modification

Study Regulatory and other findings:

This research, which involves children, meets criteria at 45 CFR 46.404 and/or 21 CFR 50.51 (research involving no greater than minimal risk). The IRB has determined that the study-specific rationale provided by the investigator in application section A.2.A is sufficient to justify this finding. Permission of one parent or guardian is sufficient.

The IRB has determined that assent of the children younger than 8 years of age may be waived according to 45 CFR 46.408(a) and/or 21 CFR 50.55(c)(1). The capability of some or all of the children (based on age, maturity or psychological state) is so limited they cannot reasonably be consulted about their willingness to participate.

Submission Regulatory and other findings:

As a reminder, although the UNC-Chapel Hill OHRE/IRB may have approved or made a determination that this study can commence, at this time UNC-Chapel Hill in response to direction from the UNC System Office has reduced campus activity significantly due to the COVID-19 outbreak. All human subject research activities are expected to follow all institutional and UNC Health policies, including those that may limit direct contact of participants. If you need to modify or alter your study design due to COVID-19 in order to conduct your research activities, please submit a modification and advise in the “Cover page” that this is “COVID-19 Related”.

This research, which was originally approved by the Full Board, is being renewed by the IRB under Expedited Review, Category 9. The Board agreed that it involves no more than minimal risk and future reviews may be done on an expedited basis.

Investigator’s Responsibilities:

Federal regulations require that all research be reviewed at least annually. It is the Principal Investigator’s responsibility to submit for renewal and obtain approval before the expiration date. You may not continue any research activity beyond the expiration date without IRB approval. Failure to receive approval for continuation before the expiration date will result in automatic termination of the approval for this study on the expiration date.

If applicable, your approved consent forms and other documents are available online at http://apps.research.unc.edu/irb/index.cfm?event=home.dashboard.irbStudyManagement&irb_id=18-2803.

You are required to obtain IRB approval for any changes to any aspect of this study before they can be implemented. Any unanticipated problem involving risks to subjects or others (including adverse events reportable under UNC-Chapel Hill policy) should be reported to the IRB using the web portal at http://irbis.unc.edu.

The current data security level determination is Level III. Any changes in the data security level need to be discussed with the relevant IT official. If data security level II and III, consult with your IT official to develop a data security plan. Data security is ultimately the responsibility of the Principal Investigator.

This study was reviewed in accordance with federal regulations governing human subjects research, including those found at 45 CFR 46 (Common Rule), 45 CFR 164 (HIPAA), 21 CFR 50 & 56 (FDA), and 40CFR 26 (EPA), where applicable.

CC:
Jonathan Juliano, Medicine-Infectious Diseases
Grace Onyebuchi, Institute for Global Health and Infectious Diseases

MBARARA UNIVERSITY OF SCIENCE AND TECHNOLOGY
P.O. Box 1410, Mbarara Uganda. Tel: +256 485433795; Fax: +256 4854 20782
RESEARCH ETHICS COMMITTEE
E-mail: sec.rec@must.ac.ug

Our Ref: MUREC 1/7 **Date: May 26, 2021**

Assoc. Prof. Edgar Mulogo Mugema
Principal Investigator

Re: Request for amendment of approved study protocol on "Rapid Tests for the evaluation of children with malaria-negative, febrile respiratory illness in rural Uganda" 14/03-19.

Type: **[ ]** Initial Application
[√] Protocol Amendment
[ ] Letter of Amendment (LOA)
[ ] Continuing Review
[ ] Material Transfer Agreement
[ ] Other, specify: ________________________________

Your request to amend the above mentioned study protocol has been received and reviewed by MUST REC.

The following proposed amendments have been approved
1. To change the study design to a stepped –wedge cluster randomized trial
2. To add a sub study to phase 3 to assess the knowledge attitudes and practices related to antibiotic use among VHTs involved in the study and to assess the feasibility and acceptability of the addition of the CRP test to their usual treatment algorithms

The following is the list of documents approved in this amendment

**Document**	**Language**	**Version, date**
Protocol	English	Version 2.0, March 01, 2021
CRP Form	English, Lhukonzo	Version 2.0 February 01, 2021
CHW Follow up Form	English, Lhukonzo	Version 2.0 February 01, 2021
VHW Pre study survey	English	
VHW Post Study survey	English	
VHW Semi-structured interview guide	English	
Primary caregiver attestation form	English, Lhukonzo	Version 2.0 February 01, 2021
Informed consent form CHW CRP	English, Lhukonzo	Version 2.0, March 10, 2021
Informed consent form CHW Child	English, Lhukonzo	Version 2.0, March 10, 2021

I wish you all the best.

Dr. Francis Bajunirwe
CHAIR,
MUST RESEARCH ETHICS COMMITTEE

THE UNIVERSITY of NORTH CAROLINA at CHAPEL HILL

OFFICE OF HUMAN RESEARCH ETHICS
720 Martin Luther King, Jr. Blvd.
Bldg. 385, 2nd Floor
CB #7097
Chapel Hill, NC 27599-7097
(919) 966-3113
Web site: ohre.unc.edu
Federalwide Assurance (FWA) #4801

To: Emily Ciccone
Medicine-Infectious Diseases

From: Biomedical IRB

Approval Date: 3/21/2022
Expiration Date of Approval: 3/20/2023
RE: Notice of IRB Approval by Expedited Review (under 45 CFR 46.110)
Submission Type: Renewal
Expedited Category: 9.Continuing rev, min risk by full IRB
Study #: 18-2803

Study Title: IGHID 12110 - Rapid Tests for Evaluation of Malaria-negative Children with Febrile Respiratory Illness in Rural Uganda

This submission, Reference ID 353152, has been approved by the IRB for the period indicated.

Study Description:

Purpose:

- Main study: The overarching goal of this study is to determine the feasibility of using rapid, point-of-care diagnostics to (a) define the epidemiology of, (b) reduce inappropriate antibiotic use for, and (c) determine disposition for malaria-negative respiratory illness among children in rural Uganda.
- Sub-study: to determine the levels of a substance called C-reactive protein or CRP in the blood of children who are being seen by VHWs with fever and cough or fast breathing. High CRP levels tell us that a child may have a bacterial infection and need antibiotic treatment. Low CRP levels tell us that the cause of illness is more likely to be a virus, which does not require antibiotic treatment. .

Participants:

- Main study: Children aged 1-10 presenting to Kasese Health Center (Phase 1) and children 5 years or younger being evaluated by community health workers (Phase 2 and 3) for fever and respiratory illness in the Kasese District of southwestern Uganda
- Sub-study: Village health workers who participate in Phase 3, Part 2

Procedures (methods):

- Main study: The study will take place in three phases:
  - Phases 1, 2, and Part 1 of Phase 3: Prospective observational cohort studies
  - Part 2 of Phase 3: Stepped-wedge cluster randomized trial
- Sub-study: Qualitative study of the village health workers who participate in Phase 3, Part 2

Summary of changes approved with this submission:

We are planning to increase the number of children we enroll in **Phase 3, Part 2** to 1,375 children. This will substantially increase the power of our study to evaluate our question of interest - the impact of the algorithm incorporating C-reactive protein testing on antibiotic prescriptions by village health workers for children with febrile acute respiratory illness. Specifically, the power to detect a difference in proportions of 0.20 with two-sided a=0.05 GEE Wald tests will be greater than 90% for a wide range of plausible intraclass correlations

Based on our previous studies, we initially anticipated being able to enroll approximately 900 children. However, enrollment has proceeded more quickly than expected, and in the first four months of the study, we have enrolled 925 children in addition to 68 of the planned 75 VHTs (while remaining under our total approved number of subjects for this application because of under-enrollment in previous phases).

Importantly, there have been no unexpected visits during the 7 day follow-up period for any of the children enrolled thus far. As this is a stepped wedge cluster randomized trial with timed transitions between the control and intervention periods for one cluster of 3 villages each month, it is imperative that we try to enroll approximately the same number of children per month.

We have uploaded an updated protocol document (v3) reflecting these changes.

Study Regulatory and other findings:

This research, which involves children, meets criteria at 45 CFR 46.404 and/or 21 CFR 50.51 (research involving no greater than minimal risk). The IRB has determined that the study-specific rationale provided by the investigator in application section A.2.A is sufficient to justify this finding. Permission of one parent or guardian is sufficient.

The IRB has determined that assent of the children younger than 8 years of age may be waived according to 45 CFR 46.408(a) and/or 21 CFR 50.55(c)(1). The capability of some or all of the children (based on age, maturity or psychological state) is so limited they cannot reasonably be consulted about their willingness to participate.

Investigator's Responsibilities:

Federal regulations require that all research be reviewed at least annually. It is the Principal Investigator's responsibility to submit for renewal and obtain approval before the expiration date. You may not continue any research activity beyond the expiration date without IRB approval. Failure to receive approval for continuation before the expiration date will result in automatic termination of the approval for this study on the expiration date.

If applicable, your approved consent forms and other documents are available online at http://irbis.research.unc.edu/irb/index.cfm?event=home.dashboard.irbStudyManagement&irb_id=18-2803.

You are required to obtain IRB approval for any changes to any aspect of this study before they can be implemented.

Promptly Reportable Information should be reported to the IRB, in IRBIS, as per OHRE SOP 1401.

The current data security level determination is Level III. Any changes in the data security level need to be discussed with the relevant IT official. If data security level II and III, consult with your IT official to develop a data security plan. Data security is ultimately the responsibility of the Principal Investigator.

This study was reviewed in accordance with federal regulations governing human subjects research, including those found at 45 CFR 46 (Common Rule), 45 CFR 164 (HIPAA), 21 CFR 50 & 56 (FDA), and 40CFR 26 (EPA), where applicable.

CC:
Adeyemi Ayankoya, Institute for Global Health and Infectious Diseases
Ross Boyce, Medicine-Infectious Diseases
Dana Giandomenico, Institute for Global Health and Infectious Diseases
Jonathan Juliano, Medicine-Infectious Diseases

MBARARA UNIVERSITY OF SCIENCE AND TECHNOLOGY
P.O. Box 1410, Mbarara Uganda. Tel: +256 485433795; Fax: +256 4854 20782
RESEARCH ETHICS COMMITTEE
E-mail: sec.rec@must.ac.ug

Our Ref: MUREC 1/7 **Date: April 19, 2022.**

Assoc. Prof. Edgar Mulogo Mugema
Principal Investigator

Re: Request for amendment of approved study protocol on "Rapid Tests for the evaluation of children with malaria-negative, febrile respiratory illness in rural Uganda" 14/03-19.

Type: [ ] Initial Application
[√] Protocol Amendment
[ ] Letter of Amendment (LOA)
[ ] Continuing Review
[ ] Material Transfer Agreement
[ ] Other, specify: ________________________________

Your request to amend the above mentioned study protocol has been received and reviewed by MUST REC.

The following proposed amendments have been approved:

1. Increase target enrollment for children in **Phase 3, Part 2** of the application to 1,375 from 900. This will increase the total accrual ceiling to 2,175.
2. Update semi-structured interview guide for the qualitative sub-study of Phase 3, Part 2
3. Update VHW pre and post-survey for the qualitative sub-study of Phase 3, Part 2
4. Addition of a data collection form to collect information on any unexpected visits the VHW that may occur during the 7-day follow-up period during Phase 3, Part 2.

The following is the list of documents approved in this amendment:

**Document**	**Language**	**Version, date**
Protocol	English	Version 3.0, March 01, 2022
CHW Unexpected Follow up Form	English	
VHW Pre study survey	English	
VHW Post Study survey	English	
VHW Semi-structured interview guide	English	

I wish you all the best.

Dr. Francis Bajunirwe
CHAIR,
MUST RESEARCH ETHICS COMMITTEE
