# Supplementary material for: Point-of-care C-reactive protein measurement by community health workers safely reduces antimicrobial use among children with respiratory illness in rural Uganda: A stepped wedge cluster randomized trial
Source: PLoS Med. 2024 Aug 19;21(8):e1004416. doi: 10.1371/journal.pmed.1004416 (PMC11407643; doi:10.1371/journal.pmed.1004416)
Supplement: Attachment — Submitted filename: PMEDICINE-D-24-01411R1_ResponsetoReviewers.docx [file pmed.1004416.s014.docx]

**RESPONSE TO REVIEWERS**

*Note: Line and page numbers referenced are based on the Tracked Changes version of the updated manuscript*

*Reviewer #1*

*This manuscript presents the findings of a stepped wedge cluster randomised trial in 15 villages in Uganda, assessing the effect of a clinical algorithm to the standard approach to guide antibiotic treatment decisions in children with fever and cough. My comments focus on the statistical aspects of this study. The statistical analyses appear to be mostly appropriate (in particular, the use of bias-corrected standard errors to account for a small number of clusters was good to see), as does the interpretation of results. However, there are several issues that require clarification and potentially re-analysis.*

*1.      In Figure 2, please provide a separate row for each village (that is, replace Figure 2 with Figure S2). Please also indicate the length of each period in days in the Period number row.*

We have replaced Figure 2 with Figure S2 and vice versa. We have also included the length of each period in days in the new Figure 2 as requested by the reviewer.

*2.      In Figure 4, why is log OR shown instead of OR? Please display OR if possible.*

We chose to display log OR because the confidence intervals are symmetric when displayed on a log scale and many readers are accustomed to seeing symmetric intervals. When not displayed logarithmically, some readers may be confused by the asymmetry of the CI with respect to the null value. A second reason that we retain the logarithmic scale in the display is to minimize redundancy. The non-transformed ORs and their confidence intervals corresponding to Figure 4 are shown in Table S5. We have now added a reference to this in the caption for Figure 4, so it is clear to readers where to find the OR estimates and their CIs.

*3.      In the Statistical Methods section, it is stated that "Similar models for prevalence differences are also reported…" It seems that these models are more fully described in the footnote of Table 3. Why were terms for period and the stratification factor not included in this model? Please provide results where period and the stratification factor have been included in the model. Why was an independence working correlation structure assumed for this outcome? Provide repeat with a nested exchangeable correlation structure and provide estimates of within-period and between-period correlations.*

In the revision, we have provided estimates of prevalence differences that adjust for period and the stratification factor, while using a working nested exchangeable correlation structure. In the original manuscript, these were not included in the model fitting for prevalence differences owing to estimation convergence issues, which commonly occur with use of the identify link function for binary data when estimated proportions (p) for some combinations of model factors do not fall between 0 and 1, which in turn results in negative Bernoulli variances p*(1-p). In the revision, we circumvented this issue by using the constant variance function resulting in model convergence. Doing so sacrificed potential statistical efficiency while retaining the validity (i.e., consistency) of the prevalence difference and its estimated variance (through bias-corrected sandwich estimators). However, this specification of GEE will not provide valid correlation estimates, so we report in Table 3 the NE correlation estimates for the odds ratio model (i.e., logit link) only. We consider this no great loss, since the correlation parameters are the same in both logit and identity link marginal mean models (being on the proportion scale), and we obtain much better estimates for them from the logistic model (i.e., consistent estimates under model assumptions being satisfied).

*4.      Randomisation into sequences was stratified - were terms for strata included in the outcome regression models? It seems from Table S2 that the stratification factor was not included in the model. Provide estimates where these terms are included.*

The strata were not originally included. In the revision, we have provided model estimates in Tables 3 and S4 based on the inclusion of both period and strata in both logit link (odds ratios) and identity link (prevalence difference) models.

*5.      The power calculation assumed a "zero slope temporal trend" - does this mean that a term for period was not included in the power calculation?*

No. Terms for periods were included in the power calculation. Power calculations based on the SAS macro CRTFASTGEEPWR are based on the design matrix for each cluster that includes both the period effects and the intervention effect. This is the case whether or not the beta-terms for period effects are null (zero). The fast GEE power also depends on the model degrees of freedom, specifically, K-p, where K is the number of clusters (villages) and p is the number of beta-terms in the model (including those from the period effects).

*6.      In Table 1, are the Village stratum labels (small, medium, large) a proxy for altitude and distance from Bugoye? If so, please include the full labels. If not, please also include altitude and distance from Bugoye in Table 1.*

The village stratum levels in Table 1 were incomplete; we thank the reviewer for pointing this out. We have edited Table 1 to include the full labels.

*7.      Include estimates of the within-period and between-period correlations in Table 3.*

We have added these estimates for the logistic model as requested. Please see our response to #3 above as to why we don’t include them for the identity link model.

*8.      Instead of using the 2010 CONSORT checklist, please use the CONSORT checklist for stepped wedge trials (*[*https://nam12.safelinks.protection.outlook.com/?url=https%3A%2F%2Fdoi.org%2F10.1136%2Fbmj.k1614&data=05%7C02%7C%7C64db28dcf0ae49afae0408dc84b8f8cf%7C58b3d54f16c942d3af081fcabd095666%7C1%7C0%7C638531177182223534%7CUnknown%7CTWFpbGZsb3d8eyJWIjoiMC4wLjAwMDAiLCJQIjoiV2luMzIiLCJBTiI6Ik1haWwiLCJXVCI6Mn0%3D%7C0%7C%7C%7C&sdata=y0hIzvFJqA2a0wPL5bOGBGK71FdnUYOcAOWnE9TDOl0%3D&reserved=0*](https://nam12.safelinks.protection.outlook.com/?url=https%3A%2F%2Fdoi.org%2F10.1136%2Fbmj.k1614&data=05%7C02%7C%7C64db28dcf0ae49afae0408dc84b8f8cf%7C58b3d54f16c942d3af081fcabd095666%7C1%7C0%7C638531177182223534%7CUnknown%7CTWFpbGZsb3d8eyJWIjoiMC4wLjAwMDAiLCJQIjoiV2luMzIiLCJBTiI6Ik1haWwiLCJXVCI6Mn0%3D%7C0%7C%7C%7C&sdata=y0hIzvFJqA2a0wPL5bOGBGK71FdnUYOcAOWnE9TDOl0%3D&reserved=0)*). Please ensure that the CONSORT stepped wedge paper is referenced and note the inclusion of the CONSORT checklist in the Supplementary materials in the manuscript.*

The CONSORT stepped wedge checklist has been completed, added to the Supplementary Materials as Checklist S1, and referenced in the manuscript under Methods – Study Design (Page 9, Lines 199-200).

*9.      Several post-hoc power calculations have been performed here - however, these have been shown to have no value (e.g. doi: 10.1007/s12144-018-0018-1; there are other references). The provided post-hoc power calculations should be removed entirely.*

We have removed them from the manuscript and the Supplemental Methods file (now Appendix S1). We would like to mention that our calculations were not intended to be post-hoc in the sense of the cited doi. Rather, we held the prevalence difference fixed at 0.20, the a priori power value, and sought to assess the sensitivity of our power calculations to chosen values of intracluster correlations and assumptions about unequal cluster sizes. While we found value in this illustrative exercise, we agree that it is not essential for the manuscript.

*10.     Provide a version of Figure S4 with a separate row for each village, and include n/N along with %.*

We have added this version as requested as Figure S5 and referenced it in the manuscript text on Page 19, Line 495.

*Reviewer #2*

*More specific targeting of antimicrobials is an important research area. A ~20% reduction in antibiotic use for ARI in the community is a valuable result. I have relatively few comments.*

*The step wedge design is a reasonable choice for this kind of intervention. However, seasonality is an issue in step wedge studies as malaria and the predominant cause of ARI in children, RSV, vary in incidence through the year. This potentially affects signs like headache, fast breathing, hypoxia, and tachycardia (as per Table 2). Malaria seems not to have been an issue for antibiotic use - ideally this should have been tested as an interaction/effect modifier. However it is conceivable that CHWs may have altered antimalarial usage. Was there any difference in prescription of antimalarials (by mRDT result)? Potential variation in viral aetiology should be discussed.*

The reviewer raises an important point regarding the impact of seasonality of malaria and viral etiology and, in turn, presenting symptoms of ARI. To minimize the impact of possible seasonal changes in the relative distribution of ARI etiologic pathogens, we intentionally enrolled individuals during both rainy and dry seasons, and there were participants enrolled under control and intervention conditions in both seasons, although the relative numbers may have differed. To account for these differences and minimize bias, our analysis took into account the period in which the child was enrolled in multiple ways (please see the Methods – Statistical Methods section on Pages 13-14 and Appendix S1 for details). We have also added text (Page 15, Lines 380-381) specifying the seasons during which the study took place.

Regarding the reviewer comments about malaria, by stratifying our results by mRDT result in Table 3, we did assess for malaria as a potential effect modifier and found that the association between the CRP algorithm use and decreased antibiotic use was similar in mRDT-positive and mRDT-negative participants. In addition, to address the reviewer question about antimalarial use, we compared antimalarial use between mRDT-positive and mRDT-negative participants, and as expected, it was much higher in the mRDT-positive group (94.4% v 2.0%). The relative use of antimalarials did not appear to be different in control and intervention periods. As this analysis was suggested by both Reviewers 2 and 3, we have added it as Table S3 and referenced it on Page 17, Lines 477-478**.**

*Power for the safety outcomes (e.g. hospitalisation) is very low, this should also be mentioned in the discussion. This could be monitored during a larger roll out.*

We mention the fact that the study was not designed to assess non-inferiority in safety outcomes in the 4^th^ paragraph of the Discussion (Page 25-26, Lines 637-645). We have added further text to specify that the study was not powered to detect differences in clinical failure or the other safety outcomes to make this point clearer in this same location, and also added it as a limitation (Page 28, Lines 701-703).

*The inclusion of  assay cost data is helpful. Given that symptoms of ARI are very common, this may represent a prohibitively high cost to a national programme. Discussion of whether this cost can be reduced either by alternative manufacturing or by using clinical features to select a group with a higher pre-test probability would be useful.*

We have added a paragraph to the Discussion regarding cost considerations (Page 26-27, Lines 655-675), while being careful not to overstate findings as we did not perform a formal cost-effectiveness analysis (a concern noted below by Reviewer #3).

*Reviewer #3:*

*This is a great manuscript presenting the results of a well-designed and executed clinical trial of the added benefit of introducing a PoC CRP measurement in the decision of prescribing (by CHW) antibiotics to children with acute respiratory infections. It is now well known that clinicians in resource constrained settings, in the absence of supportive diagnostic tools, tend to overprescribe antibiotics guided buy highly sensitive clinical algorithms that prime the use of therapeutics in the context of clinical syndromes of potentially life-threatening infections. This is particularly problematic in the field of pneumonia, where it has previously also been observed that even in the presence of moderate severity, antibiotics do not necessarily improve outcomes in comparison to placebo (possibly due to the fact that many of the moderately severe infections are not responsive to antibiotics, as they tend to be originally viral). Thus, this trial provides important data that*
*could influence policy making in terms of the added value of adding a PoC CRP evaluation at the patient's side to guide management decisions. The results are also important as they suggest the generalized use of such a PoC device could substantially decrease the use of antibiotics WITHOUT WORSENING OUTCOMES, with all the added benefit that ensues, particularly in terms of the growing threat of antimicrobial resistance. I believe the manuscript is well written, clear, and that provides a good summary of the status quo and the context on which the results of the trial could be applied. I have only some minor concerns that I would like to provide, with the hope that the manuscript is strengthened.*

**       In the differential diagnosis of the clinical syndrome of pneumonia, one needs to also consider malaria in areas where this disease is endemic. It has been shown also that CRP is often similarly elevated in response to acute malaria that in response to bacterial infections. Did authors screen for malaria in the setting where the trial was conducted (rural Uganda, where presumably there is lots of malaria), and where they able to analyse how many mixed infections there were, and how well did the CRP evaluation perform in those cases where malaria infections where confirmed? If this was not done, perhaps this should be acknowledged in the discussion. In this context, and given what CHW are trained to do, where all cases suspected of pneumonia which ALSO had a fever tested for malaria?*

The reviewer makes an important point about the overlap in the clinical presentation of malaria and pneumonia. In this study, subjective or documented fever was one of the inclusion criteria, so all participants had this symptom and per CHW protocols were tested for malaria by mRDT (results are missing for 2 participants in the intervention group as shown in Table 2). We noted a surprisingly high rate of mRDT positivity (~50% in both control and intervention groups), whereas our previous studies of febrile illness and ARI in the region have noted closer to 20-30% positivity.(1,2) In Table 3, we have reported the differences in antibiotic use by mRDT result and the magnitude of the difference in antibiotic use between control and intervention is similar in mRDT-positive and negative individuals. We have also added a panel to Figure 5 that shows the distribution of CRP results stratified by mRDT result. Overall, the distribution was surprisingly similar, although the proportion of individuals with a CRP result of <10mg/L was lower in mRDT-positive as compared to mRDT-negative children. This has also been referenced in the Results – CRP Test Results section (Page 22, Lines 560-562).

**       Methods: What kind of amoxicillin was used? Did the trial use conventional or dispersible?*

The CHW dispensed dispersible tables. This detail was added to the Methods – Study Intervention section (Page 10, Line 236). At the time of prescription, the CHW demonstrates how to dissolve the tablet on a spoon with water for feeding to the child and administer the first dose.

**       Method: please specify whether pulsioximetry was used in every recruited patient, and if so whether there were differences in outcomes and performance of CRP according to the presence or absence of hypoxemia. I guess it wasn't used, but then it should be specifically mentioned so that the reader understands this is not routine care by CHWs*

Only one CHW per village was provided with a pulse oximeter due to limited funding. This is described in the Methods – Clinical Definitions, which we have edited to clarify what was part of the CHW program and what was additionally measured as part of the study (Page 12, Lines 286-287). In response to this comment, we conducted a descriptive analysis quantifying antibiotic use in control versus intervention groups stratified by hypoxia, and results are show in the table below. As only one CHW per village was given a pulse oximeter, this analysis includes only 257 individuals. It should be noted that, due to funding limitations, the portable pulse oximeters used in this study were not designed for pediatric patients, and therefore, may not be accurate especially in children under 2 years. We have added this as a limitation in the Discussion (Page 28, Line 710-712) and elected not to add the data below to the paper. However, should the reviewers and editors think it should be included, we are happy to add it as a Supplemental Table.

|  | **Hypoxic** | **Not Hypoxic** |
| --- | --- | --- |
| ***Intervention*** | | |
| Antibiotic given | 76.9% | 68.1% |
| Antibiotic NOT given | 23.1% | 31.9% |
| ***Control*** | | |
| Antibiotic given | 95.3% | 86.6% |
| Antibiotic NOT given | 4.7% | 13.4% |

**       I believe iCCM protocols regarding pneumonia, danger signs, and management recommendations should be more explicitly explained. Perhaps this can be presented as a supplementary table or panel*

We feel the relevant components of the routine iCCM protocols as they relate to management of respiratory illness are sufficiently explained in the Methods – Study Intervention section (Page 10-11). However, in response to this comment, we have added references to a manuscript and the Ministry of Health website where the protocols are fully described and a copy of the iCCM Sick Child Job Aid is available for download (Page 10, Lines 237-239).

**       In the methods section please define: what is the HIV prevalence rate estimated in the area.*

The prevalence of HIV among children age 0-14 in Uganda as a whole is estimated at 0.5% (95% CI 0.3-0.8).(3) This has been added to the Methods in the Study Setting section (Page 8, Line 189).

**       What are the routine vaccines against pneumonia currently in use in the area. Where there changes or additions of new vaccines or different ones throughout the development of the trial?*

The routine vaccination schedule includes 3 doses of Haemophilus influenzae type B vaccine and 3 doses of pneumococcal conjugate vaccine (10-valent) prior to 4 months of age. This information has been added to the Methods – Study Setting section (Page 8, Lines 186-188). There were no changes or additions during the study period.

**       Inclusion and exclusion criteria: "if the previous illness had completely resolved, and they were re-presenting for a new episode of ARI". Was there a lag period considered for this re-inclusion? In other words, if a child presented with pneumonia, and somehow resolution of the symptoms, was there a minimum number of days that needed to pass before considering the child again eligible?*

We did not specify a minimum number of days in the study protocol. Determination of resolution of symptoms was left to the discretion of the CHW. We have added this information to the Methods – Study Participants section (Page 12, Line 280-281).

**       I believe what the pulsioximeter defines is hypoxemia rather than hypoxia. Please verify*

These terms are often used interchangeably even though they mean slightly different things. Hypoxemia refers to low levels of oxygen in the arterial blood, which is usually assessed through measurement of the arterial oxygen tension or PaO2 using an arterial blood gas. We did not measure this due to its invasiveness and the level of training of the CHW implementing the study. Therefore, hypoxia is the more accurate term as pulse oximetry measurements can be affected by factors other than PaO2.

**       Was weight measured? If yes, wouldn't it be better to define malnutrition to use weight rather than MUAC?*

We do not have weight measurements for the participants. The CHWs do not have a scale provided to them through the program, and a full nutritional assessment including weight and length/height is not routinely performed by the CHW as part of their evaluation of sick children. In determining what additional components to add to the CHW evaluation (and, in turn, their documentation requirements) as part of the study, we were cognizant of not over-taxing them. MUAC is routinely used locally as a quick and cost-effective way of assessing nutritional status in children. We therefore opted for this method of nutritional assessment instead of weight and height.

**       I believe a brief description of the CHWs may be useful to understand whether there could be important differences among them in terms of training and expertise, that could somehow have confounded the results*

We have added basic socioeconomic data about the CHW in what is now Table S2, referencing it in the Results – Baseline Characteristics section (Page 16, Line 453-454). It is important to note that the same CHW enrolled children in both control and intervention periods, so we do not expect differences in training and expertise to significantly confound the results.

**       Regarding CRP thresholds and values, as obtained at the PoC. What were the recommendations (and training) given to the CHW in terms of how should they interpret CRP results?*

Prior to study start, all participating CHW underwent a hands-on training on the performance and interpretation of the Actim CRP test by laboratory personnel from Bugoye Health Centre III as well as the study staff and principal investigator, all of whom had experience using the Actim CRP test. The CHW were also given written instructions including pictures to refer to if needed during study implementation and participated in refresher trainings between each period. This information has been added to the Methods – Study Intervention section (Page 11, Lines 255-259). The Actim CRP is a lateral flow-based test, similar to the malaria rapid diagnostic tests with which the CHW were already very familiar. The interpretation of the result requires only counting of lines on a strip. More details on the test itself are available at this website: <https://www.actimtest.com/actim-crp/>

**       As one of the secondary endpoints was death, you should state whether there were any deaths in the trial and in each of the groups*.

There were no deaths in either group during the trial. This is mentioned in the Results – Clinical Failure and Adverse Events section (Page 21, Lines 535-536).

**       I believe the small paragraph on costs and estimated costs averted is useful, but either you should include a bit more detailed info or tone down the conclusions, given that the study was not really designed to assess cost effectiveness*

Unfortunately, we do not have more detailed information regarding cost to share as this was not a main focus of the study. However, given the importance of cost on potential uptake of the CRP test, we felt it was important to include even if briefly. Reviewer #2 suggested additional discussion of the impact of cost – this has been added to the Discussion as described above. We were cautious in our conclusions and attempted to make it clear that our analysis was crude and not intended to be a formal cost-effective analysis.

**       The manuscript does not discuss one of the hot topics of risk stratification and triage issues more relevant to work in settings with scarcity of resources, which is the idea of prognosis vs. diagnosis. Given that your intervention (PoC CRP test) is essentially a guide to ascertain whether the underlying cause of pneumonia is of likely bacterial or non bacterial origin), your endpoint is rightly designed as use of antibiotics. It could also very well be that CRP tells you, in advance, which children are more likely to go well (or unwell, and have an adverse outcome) with prognosis becoming more important. I would personally appreciate if the authors could reflect a bit on these implications, and about the different risk stratification approaches to improve pneumonia outcomes.*

We agree with the reviewer that this is a very interesting discussion. Given that it is known that the sensitivity and specificity of CRP is moderate for identification of bacterial infection, it is certainly possible that in fact what a high CRP represents is severe infection and risk of poor outcomes. As we work to optimize clinical decision algorithms for pneumonia that can be successfully employed by providers with limited medical training in LMIC, it is crucial to have both improved prognostication and reduction of inappropriate antibiotic use as goals. We have added mention of this to the Discussion on Page 26, Lines 645-654.

**       Table 1: besides guardian's occupation, do you have info on guardian's level of education? This may be more relevant in relation to outcomes than occupation*

We did not collect information on the guardian’s level of education.

**       Regarding the analysis stratified by malaria RDT, do you have also data on those who received (or did not) an antimalarial? Could you also stratify by reception (or not) of antimalarials?*

We do have these data. Please see response to Reviewer #2 above.

**       Figure 5 could benefit from adding a vertical line in the value considered threshold for recommendation of antibiotic use*

This has been added.

*Comments from the Academic Editor:*

*1) The authors have not provided a rational for why the step-wedge design was needed. My feeling here is that a parallel group cluster randomised trial would have been a better choice, and would be be at lower risk of bias. One justification for step-wedge trials is that the intervention is going to be introduced into routine practice, and so this design allows evaluation during implementation. If the authors want to make this argument, it should be justified by strong evidence showing that point of care CRP is now widely implemented and maintained by the Ministry of Health in this setting. Please justify the selection of step-wedge design in terms of scientific rationale to address their hypothesis (rather than logistics).*

We chose a stepped wedge design for several reasons. First, as discussed in the manuscript, several studies conducted in outpatient health care facilities have demonstrated that incorporation of C-reactive protein (CRP) testing into the evaluation of children presenting with febrile respiratory illness can significantly reduce antibiotic use without increasing adverse outcomes, but CRP measurement by non-medically trained providers like CHW had not yet been evaluated. Therefore, the effectiveness goal (as opposed to efficacy) of our intervention was well suited to a pragmatic cluster randomized trial design. Second, the demonstrated efficacy of the intervention in other settings supported implementing it broadly in our study setting. Third, the Ugandan co-investigators felt the study would be best received by the community and specifically the CHW program should all participating villages have the opportunity to receive the intervention; in other words, stepped wedge design facilitated cluster recruitment and enhanced acceptability of the study. Finally, this was a comparative effectiveness trial of limited scope in a low resource setting primarily funded by a small early career award; the stepped wedge design allowed us to conduct a randomized evaluation within our resource constraints, engaging 65 CHW in 15 villages. To this latter point, logistical factors are among the potentially appropriate justifications for choosing a stepped wedge design outlined by Hemming and Taljaard, 2020,(4) (who also contributed to the CONSORT reporting guidelines for stepped wedge trials (5)) even recognizing the potential biases due to temporal trends intrinsic to the stepped wedge study design. We followed best practice in the statistical analysis of our primary outcome with the inclusion of categorical period effects in our models for both the intervention odds ratio and prevalence difference. This is the recommended and least restrictive modeling approach that adjusts for temporal trends.

Another potential justification discussed by Hemming and Taljaard is that a stepped wedge design could increase statistical power over other study designs.(4) We did not explicitly compare the power associated with a parallel versus a stepped wedge design during the study planning phase, but as we report in the paper, we had excellent power with our stepped wedge design.

A brief sentence regarding rationale for the stepped wedge design has been added to the Introduction as per CONSORT reporting of stepped wedge trial recommendations (Page 7, Lines 156-159).

*2) Statistical analysis: mostly rigorously done. However, given the stratified randomisation schedule, the authors should have probably adjusted regression model estimates of effect for stratification variables - not doing so usually leads to inflated effect sizes (*[*https://nam12.safelinks.protection.outlook.com/?url=https%3A%2F%2Ftrialsjournal.biomedcentral.com%2Farticles%2F10.1186%2Fs13063-020-04850-w&data=05%7C02%7C%7C64db28dcf0ae49afae0408dc84b8f8cf%7C58b3d54f16c942d3af081fcabd095666%7C1%7C0%7C638531177182196282%7CUnknown%7CTWFpbGZsb3d8eyJWIjoiMC4wLjAwMDAiLCJQIjoiV2luMzIiLCJBTiI6Ik1haWwiLCJXVCI6Mn0%3D%7C0%7C%7C%7C&sdata=1G87gY0MH1RJ%2FdZ1BBy5r9JIGrMpQTmhUhreDFgqAeI%3D&reserved=0*](https://nam12.safelinks.protection.outlook.com/?url=https%3A%2F%2Ftrialsjournal.biomedcentral.com%2Farticles%2F10.1186%2Fs13063-020-04850-w&data=05%7C02%7C%7C64db28dcf0ae49afae0408dc84b8f8cf%7C58b3d54f16c942d3af081fcabd095666%7C1%7C0%7C638531177182196282%7CUnknown%7CTWFpbGZsb3d8eyJWIjoiMC4wLjAwMDAiLCJQIjoiV2luMzIiLCJBTiI6Ik1haWwiLCJXVCI6Mn0%3D%7C0%7C%7C%7C&sdata=1G87gY0MH1RJ%2FdZ1BBy5r9JIGrMpQTmhUhreDFgqAeI%3D&reserved=0)*). This point was also raised by the statistical reviewer.*

In the revised manuscript, we adjusted models for the stratification factors. Please see our reply to Reviewer #1.

*3) Whilst the author's interpretation of the primary outcome is probably justified, the secondary outcomes are very imprecisely estimated due to small numbers of events. I am not convinced that this trial provides high quality evidence that the intervention did not worse adverse outcomes - it is really very difficult to say one way or the other here. (e.g. main conclusion statement is probably overstated: "“As compared to the standard iCCM algorithm, the use of the STAR SCJA was associated with a significant decrease in antibiotic use without a concomitant increase in adverse outcomes.”*

We agree, so we have edited the quoted text in the first paragraph of the Discussion section as follows: “As compared to the standard iCCM algorithm, the use of the STAR SCJA was associated with a significant decrease in antibiotic use without a statistically significant concomitant increase in adverse outcomes.” We provide comment on the small number of events in the expanded limitations section and elsewhere in the discussion. We have also edited the Conclusions section of the abstract to acknowledge the small number of adverse events (Lines 56-57)

*4) Manuscript needs to be reported in accordance with CONSORT statement for stepped wedge cluster-randomized trial (also see point of the statistical reviewer)*

This has been done. Please see response to Reviewer #1.

*6) Expand limitations. Limitations section is a little brief, and perhaps overly optimistic. Would like greater reflection on risk of bias.*

We have expanded the limitations section. Specifically, we added: “Because many of the secondary dichotomous outcomes have low rates of events, our study provided relatively poor precision to compare the rates between control and intervention treatment conditions resulting in wide confidence intervals.” In addition, we added discussion of several potential biases associated with stepped wedge trials and efforts we took to mitigate those risks. (Page 18, Line 672-677).


*-----------------------------------------------------------*
*Requests from the editors:*

*1) Please include a short summary of protocol approvals and why the registration was late in the method section. We appreciate you have added a short explanation in the manuscript (Methods), but please expand a bit so that the reader has a short summary of the events without the need to consult the SI. Please keep the full details as SI. Please also acknowledge possible investigator bias this may have introduced.*

We have added this short summary and acknowledged possible investigator bias in the Methods (Page 15, Lines 375-376) as well as where it was already acknowledged in the Discussion as requested. Please let us know if more detail should be included.

*2) Reporting guidance*
 
*Please use the 'Reporting of stepped wedge cluster randomised trials' CONSORT checklist (apologies for not having made that clear in my previous request)*

This has been done. Please see response to Reviewer #1.

*3) Data Availability* 

*PLOS Medicine requires that the de-identified data underlying the specific results in a published article be made available, without restrictions on access, in a public repository or as Supporting Information at the time of article publication, provided it is legal and ethical to do so. Please see the policy at* [*https://nam12.safelinks.protection.outlook.com/?url=http%3A%2F%2Fjournals.plos.org%2Fplosmedicine%2Fs%2Fdata-availability&data=05%7C02%7C%7C64db28dcf0ae49afae0408dc84b8f8cf%7C58b3d54f16c942d3af081fcabd095666%7C1%7C0%7C638531177182200914%7CUnknown%7CTWFpbGZsb3d8eyJWIjoiMC4wLjAwMDAiLCJQIjoiV2luMzIiLCJBTiI6Ik1haWwiLCJXVCI6Mn0%3D%7C0%7C%7C%7C&sdata=sBCuQ1ivTs2%2BszsXiBmY6qE2PGiPHSX8OP0inqzlc0c%3D&reserved=0*](https://nam12.safelinks.protection.outlook.com/?url=http%3A%2F%2Fjournals.plos.org%2Fplosmedicine%2Fs%2Fdata-availability&data=05%7C02%7C%7C64db28dcf0ae49afae0408dc84b8f8cf%7C58b3d54f16c942d3af081fcabd095666%7C1%7C0%7C638531177182200914%7CUnknown%7CTWFpbGZsb3d8eyJWIjoiMC4wLjAwMDAiLCJQIjoiV2luMzIiLCJBTiI6Ik1haWwiLCJXVCI6Mn0%3D%7C0%7C%7C%7C&sdata=sBCuQ1ivTs2%2BszsXiBmY6qE2PGiPHSX8OP0inqzlc0c%3D&reserved=0)  
*and FAQs at*  
[*https://nam12.safelinks.protection.outlook.com/?url=http%3A%2F%2Fjournals.plos.org%2Fplosmedicine%2Fs%2Fdata-availability%23loc-faqs-for-data-policy&data=05%7C02%7C%7C64db28dcf0ae49afae0408dc84b8f8cf%7C58b3d54f16c942d3af081fcabd095666%7C1%7C0%7C638531177182205546%7CUnknown%7CTWFpbGZsb3d8eyJWIjoiMC4wLjAwMDAiLCJQIjoiV2luMzIiLCJBTiI6Ik1haWwiLCJXVCI6Mn0%3D%7C0%7C%7C%7C&sdata=LIUSDwbYvXu2Is6Y%2BQSmj5OlLhvlTI6tlqb0Hbeg6sw%3D&reserved=0*](https://nam12.safelinks.protection.outlook.com/?url=http%3A%2F%2Fjournals.plos.org%2Fplosmedicine%2Fs%2Fdata-availability%23loc-faqs-for-data-policy&data=05%7C02%7C%7C64db28dcf0ae49afae0408dc84b8f8cf%7C58b3d54f16c942d3af081fcabd095666%7C1%7C0%7C638531177182205546%7CUnknown%7CTWFpbGZsb3d8eyJWIjoiMC4wLjAwMDAiLCJQIjoiV2luMzIiLCJBTiI6Ik1haWwiLCJXVCI6Mn0%3D%7C0%7C%7C%7C&sdata=LIUSDwbYvXu2Is6Y%2BQSmj5OlLhvlTI6tlqb0Hbeg6sw%3D&reserved=0)   


*The Data Availability Statement (DAS) requires revision. For each data source used in your study:*  

*a) If the data are freely or publicly available, note this and state the location of the data: within the paper, in Supporting Information files, or in a public repository (include the DOI or accession number).* 

*b) If the data are owned by a third party but freely available upon request, please note this and state the owner of the data set and contact information for data requests (web or email address). Note that a study author cannot be the contact person for the data.* 

*c) If the data are not freely available, please describe briefly the ethical, legal, or contractual restriction that prevents you from sharing it. Please also include an appropriate contact (web or email address) for inquiries (again, this cannot be a study author).* 
The data availability statement has been updated in concordance with the journal requirements.

*Statistical reporting* 

*Were relevant, please quantify the main results with 95% CIs and p values.*

We have reviewed all the data in the manuscript and ensured this information is present where applicable.

*When reporting p values please report as <0.001 and where higher as p=0.002, for example. When reporting 95% CIs please separate upper and lower bounds with commas instead of hyphens as the latter can be confused with reporting of negative values. Please include any important dependent variables that are adjusted for in the analyses.* 

Done.

*Abstract layout* 
*Please ensure your report your abstract according to CONSORT for abstracts, following the PLOS Medicine abstract structure (Background, Methods and Findings, Conclusions)* [*https://nam12.safelinks.protection.outlook.com/?url=https%3A%2F%2Fwww.equator-network.org%2Freporting-guidelines%2Fconsort-abstracts%2F&data=05%7C02%7C%7C64db28dcf0ae49afae0408dc84b8f8cf%7C58b3d54f16c942d3af081fcabd095666%7C1%7C0%7C638531177182210083%7CUnknown%7CTWFpbGZsb3d8eyJWIjoiMC4wLjAwMDAiLCJQIjoiV2luMzIiLCJBTiI6Ik1haWwiLCJXVCI6Mn0%3D%7C0%7C%7C%7C&sdata=DSdhxVvUc2RRHzKY9qEeaPlFZAxwBqRSai6gTFv3M7U%3D&reserved=0*](https://nam12.safelinks.protection.outlook.com/?url=https%3A%2F%2Fwww.equator-network.org%2Freporting-guidelines%2Fconsort-abstracts%2F&data=05%7C02%7C%7C64db28dcf0ae49afae0408dc84b8f8cf%7C58b3d54f16c942d3af081fcabd095666%7C1%7C0%7C638531177182210083%7CUnknown%7CTWFpbGZsb3d8eyJWIjoiMC4wLjAwMDAiLCJQIjoiV2luMzIiLCJBTiI6Ik1haWwiLCJXVCI6Mn0%3D%7C0%7C%7C%7C&sdata=DSdhxVvUc2RRHzKY9qEeaPlFZAxwBqRSai6gTFv3M7U%3D&reserved=0)

We have reviewed the SW-CRT CONSORT checklist for abstracts and made minor edits to the abstract in the manuscript to ensure all elements were clearly reported (Page 2-3).

*Author summary* 

*At this stage, we ask that you include a short, non-technical Author Summary of your research to make findings accessible to a wide audience that includes both scientists and non-scientists. The authors summary should consist of 2-3 succinct bullet points under each of the following headings:* 

*• Why Was This Study Done? Authors should reflect on what was known about the topic before the research was published and why the research was needed.* 

*• What Did the Researchers Do and Find? Authors should briefly describe the study design that was used and the study’s major findings. Do include the headline numbers from the study, such as the sample size and key findings.*     

*• What Do These Findings Mean? Authors should reflect on the new knowledge generated by the research and the implications for practice, research, policy, or public health. Authors should also consider how the interpretation of the study’s findings may be affected by the study limitations. In the final bullet point of ‘What Do These Findings Mean?’, please describe the main limitations of the study in non-technical language.* 

*The Author Summary should immediately follow the Abstract in your revised manuscript. This text is subject to editorial change and should be distinct from the scientific abstract. Please see our author guidelines for more information:* [*https://nam12.safelinks.protection.outlook.com/?url=https%3A%2F%2Fjournals.plos.org%2Fplosmedicine%2Fs%2Frevising-your-manuscript%23loc-author-summary&data=05%7C02%7C%7C64db28dcf0ae49afae0408dc84b8f8cf%7C58b3d54f16c942d3af081fcabd095666%7C1%7C0%7C638531177182214539%7CUnknown%7CTWFpbGZsb3d8eyJWIjoiMC4wLjAwMDAiLCJQIjoiV2luMzIiLCJBTiI6Ik1haWwiLCJXVCI6Mn0%3D%7C0%7C%7C%7C&sdata=VczM2q7r7bPaCKPEHjMHMvnN6Vx%2FI1gHyLc67a6BELM%3D&reserved=0*](https://nam12.safelinks.protection.outlook.com/?url=https%3A%2F%2Fjournals.plos.org%2Fplosmedicine%2Fs%2Frevising-your-manuscript%23loc-author-summary&data=05%7C02%7C%7C64db28dcf0ae49afae0408dc84b8f8cf%7C58b3d54f16c942d3af081fcabd095666%7C1%7C0%7C638531177182214539%7CUnknown%7CTWFpbGZsb3d8eyJWIjoiMC4wLjAwMDAiLCJQIjoiV2luMzIiLCJBTiI6Ik1haWwiLCJXVCI6Mn0%3D%7C0%7C%7C%7C&sdata=VczM2q7r7bPaCKPEHjMHMvnN6Vx%2FI1gHyLc67a6BELM%3D&reserved=0) 

This has been added on Pages 4-5.

*Introduction layout* 

*Please address past research and explain the need for and potential importance of your study. Indicate whether your study is novel and how you determined that. If there has been a systematic review of the evidence related to your study (or you have conducted one), please refer to and reference that review and indicate whether it supports the need for your study.* 

Done. 

*Discussion layout* 

*Please present and organize the Discussion as follows: a short, clear summary of the article's findings; what the study adds to existing research and where and why the results may differ from previous research; strengths and limitations of the study; implications and next steps for research, clinical practice, and/or public policy; one-paragraph conclusion.* 

Done.

*Supplementary materials*  

*Please note that supplementary materials are not checked and will be posted as supplied by the authors. Therefore, please double check. Please cite your Supporting Information as outlined here:* [*https://nam12.safelinks.protection.outlook.com/?url=https%3A%2F%2Fjournals.plos.org%2Fplosmedicine%2Fs%2Fsupporting-information&data=05%7C02%7C%7C64db28dcf0ae49afae0408dc84b8f8cf%7C58b3d54f16c942d3af081fcabd095666%7C1%7C0%7C638531177182219054%7CUnknown%7CTWFpbGZsb3d8eyJWIjoiMC4wLjAwMDAiLCJQIjoiV2luMzIiLCJBTiI6Ik1haWwiLCJXVCI6Mn0%3D%7C0%7C%7C%7C&sdata=zhA2RlW%2Bf52zNif24zU1FMpE6DJd20PxiPcu8%2FR%2BGyw%3D&reserved=0*](https://nam12.safelinks.protection.outlook.com/?url=https%3A%2F%2Fjournals.plos.org%2Fplosmedicine%2Fs%2Fsupporting-information&data=05%7C02%7C%7C64db28dcf0ae49afae0408dc84b8f8cf%7C58b3d54f16c942d3af081fcabd095666%7C1%7C0%7C638531177182219054%7CUnknown%7CTWFpbGZsb3d8eyJWIjoiMC4wLjAwMDAiLCJQIjoiV2luMzIiLCJBTiI6Ik1haWwiLCJXVCI6Mn0%3D%7C0%7C%7C%7C&sdata=zhA2RlW%2Bf52zNif24zU1FMpE6DJd20PxiPcu8%2FR%2BGyw%3D&reserved=0) *- Please note you may use almost any description as the item name of your supporting information as long as it contains an "S" and number. For example, “S1 Appendix” and “S2 Appendix,” “S1 Table” and “S2 Table. Please ensure each supplementary material has a call out (link) from your main manuscript.*

Each supplemental table and figure has been uploaded as a separate file, and the supplemental methods and regulatory materials files have been uploaded as Appendices S1 and S2.

**References cited in this Response Letter**

1.	Boyce RM, Collins M, Muhindo R, Nakakande R, Ciccone EJ, Grounds S, et al. Dengue in Western Uganda: a prospective cohort of children presenting with undifferentiated febrile illness. BMC Infect Dis. 2020 Nov 11;20(1):835.

2.	Ciccone EJ, Kabugho L, Baguma E, Muhindo R, Juliano JJ, Mulogo E, et al. Rapid Diagnostic Tests to Guide Case Management of and Improve Antibiotic Stewardship for Pediatric Acute Respiratory Illnesses in Resource-Constrained Settings: a Prospective Cohort Study in Southwestern Uganda. Microbiol Spectr. 2021 Dec 22;9(3):e0169421.

3.	Ministry of Health, Uganda. Uganda Population-based HIV Impact Assessment (UPHIA) 2016-2017: Final Report. [Internet]. Kampala, Uganda; 2019 Jul [cited 2024 Jun 6]. Available from: https://phia.icap.columbia.edu/uganda-phia-final-report/, https://phia.icap.columbia.edu/uganda-phia-final-report/

4.	Hemming K, Taljaard M. Reflection on modern methods: when is a stepped-wedge cluster randomized trial a good study design choice? Int J Epidemiol. 2020 Jun 1;49(3):1043–52.

5.	Hemming K, Taljaard M, McKenzie JE, Hooper R, Copas A, Thompson JA, et al. Reporting of stepped wedge cluster randomised trials: extension of the CONSORT 2010 statement with explanation and elaboration. BMJ. 2018 Nov 9;k1614.
