# Supplementary material for: Point-of-care C-reactive protein measurement by community health workers safely reduces antimicrobial use among children with respiratory illness in rural Uganda: A stepped wedge cluster randomized trial
Source: PLoS Med. 2024 Aug 19;21(8):e1004416. doi: 10.1371/journal.pmed.1004416 (PMC11407643; doi:10.1371/journal.pmed.1004416)
Supplement: Attachment — Submitted filename: PMEDICINE-D-24-01411R2_ResponsetoEditors.docx [file pmed.1004416.s015.docx]

**RESPONSE TO EDITORS’ REQUESTS**

*Note: Line and page numbers referenced are based on the Tracked Changes version of the updated manuscript*

*Thank you for your detailed response to the editors' and reviewers' comments. I have discussed the paper with my colleagues and the academic editor, and it has also been seen again by the original reviewers. The changes made to the paper were satisfactory to the reviewers and editors alike.*

*We only have a few minor comments for you to address.*

*1. Regarding the choice of CRP>40, we appreciate there is some discussion of this in the Discussions section already, but a sentence on this in the Background section would be helpful for the readers.*

This information has been added on Page 7, Lines 120-122 and 124-126.

*2. Consent: Please provide additional details regarding participant consent. Your study included minors, and you state that you obtained consent from parents or guardians. In the ethics statement in the Methods and online submission information, please ensure that you have specified what type you obtained (for instance, written or verbal, and if verbal, how it was documented and witnessed).*

This information has been added to the Research Ethics Review and Trial Registration Section on Page 15, Line 319.

*3. In Table 1. Demographics of the study population, one of the occupations listed is 'peasant'. I wondered if it would be better to label this to 'agricultural laborer' or something alike, given the negative connotations associated with the word 'peasant'.*

Thank you for pointing out this concern. While the term “peasant” is what is used locally in Uganda, we agree that it has negative connotations. We have replaced it with “subsistence farmer,” which more accurately describes the participant’s caregivers’ occupation.

*4. References: For in-text reference, citations are placed within square parentheses [ ] and should precede punctuation. Please amend.*

The citation formatting has been corrected as requested above.

*PLOS uses the numbered citation (citation-sequence) method and first six authors, et al.*

*Please ensure that journal name abbreviations match those found in the National Center for Biotechnology Information (NCBI) databases (https://nam12.safelinks.protection.outlook.com/?url=http%3A%2F%2Fwww.ncbi.nlm.nih.gov%2Fnlmcatalog%2Fjournals&data=05%7C02%7C%7C14a7e41771f64d16a73008dca64f414b%7C58b3d54f16c942d3af081fcabd095666%7C1%7C0%7C638568106535650541%7CUnknown%7CTWFpbGZsb3d8eyJWIjoiMC4wLjAwMDAiLCJQIjoiV2luMzIiLCJBTiI6Ik1haWwiLCJXVCI6Mn0%3D%7C0%7C%7C%7C&sdata=FNrGBVEhkgzTKb94%2FLvWLRmvO6XjQ7IeenvAf1auItU%3D&reserved=0), and are appropriately formatted and capitalised.*

*For e.g reference 12, 37, 38, 39, etc : Where website addresses are cited, please specify the date of access (e.g. add [accessed: 16/09/2023] - instead of [cited]). Please amend.*

The website references have been amended as requested above. All references have been reviewed and confirmed as correct. Of note, current reference #3 was moved earlier in the same sentence to more clearly match it with the text to which it referred, and reference #21 was added to provide further evidence to support the sentence on page 6, lines 116-118. Finally, we corrected an unlinked reference on page 17, line 307.

*Please see https://nam12.safelinks.protection.outlook.com/?url=https%3A%2F%2Fjournals.plos.org%2Fplosmedicine%2Fs%2Fsubmission-guidelines%23loc-references&data=05%7C02%7C%7C14a7e41771f64d16a73008dca64f414b%7C58b3d54f16c942d3af081fcabd095666%7C1%7C0%7C638568106535655452%7CUnknown%7CTWFpbGZsb3d8eyJWIjoiMC4wLjAwMDAiLCJQIjoiV2luMzIiLCJBTiI6Ik1haWwiLCJXVCI6Mn0%3D%7C0%7C%7C%7C&sdata=icqSVI%2BJMU8CJ3lCu8DTZ%2B%2FMSc5feWSQl2H7ZeT8vUI%3D&reserved=0 for further details on reference formatting.*

*5. ACKNOWLEDGMENTS/ DECLARATIONS*

*Please remove all statements apart from acknowledgements, author contributions and abbreviations from the end of the main manuscript and include these only in the relevant parts of the manuscript submission form. Funding, competing interest, and data availability will be compiled as metadata.*

The funding information has been removed from the manuscript file.

*General comment:*

*This is a kind reminder that supplementary materials are not checked and will be posted as supplied by the authors. Therefore, please double check. Please cite your Supporting Information as outlined here: https://nam12.safelinks.protection.outlook.com/?url=https%3A%2F%2Fjournals.plos.org%2Fplosmedicine%2Fs%2Fsupporting-information&data=05%7C02%7C%7C14a7e41771f64d16a73008dca64f414b%7C58b3d54f16c942d3af081fcabd095666%7C1%7C0%7C638568106535660414%7CUnknown%7CTWFpbGZsb3d8eyJWIjoiMC4wLjAwMDAiLCJQIjoiV2luMzIiLCJBTiI6Ik1haWwiLCJXVCI6Mn0%3D%7C0%7C%7C%7C&sdata=zAzmoMmGeQ%2FV2Eetw%2B7jKDwW8cpu011I2GIbTSTl6Ww%3D&reserved=0 - Please note you may use almost any description as the item name of your supporting information as long as it contains an "S" and number. For example, “S1 Appendix” and “S2 Appendix,” “S1 Table” and “S2 Table.*

*Please also ensure each supplementary material has a call out (link) from your main manuscript.*

The supplementary materials have been reviewed. The following edits were made:

- Table S1: added sentence to caption clarifying that proportions of antibiotic use shown are unadjusted.
- Appendix S2: Protocol v3.0 was inadvertently omitted from the version submitted previously. It has now been added following Protocol v2.0.
